# Hydration channel engineering in Tröger’s base-POP membranes for mono/multivalent cation and anion discrimination

**DOI:** 10.1126/sciadv.aef9368

**Published:** 2026-10-09

**Authors:** Haopan Sun, Ning Gan, Baolong Wu, Chenglin Liu, Yan Jin, Yuzhu Sun, Jianguo Yu, Zhaoliang Cui, Yuqing Lin, Hideto Matsuyama, Young Moo Lee

**Affiliations:** ^1^ National Engineering Research Center for Comprehensive Utilization of Salt Lake Resources, School of Chemical Engineering, East China University of Science and Technology, Shanghai 200237, China.; ^2^ School of Chemical Engineering, Nanjing Tech University, Nanjing 211816, China.; ^3^ Research Center for Membrane and Film Technology, Department of Chemical Science and Engineering, Kobe University, Kobe 657-8501, Japan.; ^4^ Department of Energy Engineering, College of Engineering, Hanyang University, Seoul 04763, Republic of Korea.

## Abstract

Sustainable recovery of critical resources from complex aqueous streams is constrained by membranes that fail to combine rapid ion transport with precise mono/multivalent discrimination. Here, we report Tröger’s base-derived porous organic polymer (POP) membranes with rigid, covalently crosslinked microporous frameworks that stabilize angstrom-scale transport channels while suppressing hydration-induced swelling. These membranes render rapid monovalent-ion transport with near-complete exclusion of multivalent cations and anions, including Mg^2+^, Al^3+^, SO_4_^2−^, and PO_4_^3−^. Experiments and simulations show that steric confinement, dehydration penalties, and electrostatic interactions cooperatively raise migration barriers for multivalent ions in confined channels. The membranes achieve comprehensive mono/multivalent discrimination across both cationic and anionic systems. In multistage electrodialysis, they enable stage-amplified lithium enrichment from high-Mg brines with stable long-term operation and downstream recovery of crystalline Li_2_CO_3_. They also facilitate high-purity NaCl enrichment from simulated seawater. Overall, this platform offers a durable and energy-efficient route to selective resource recovery from complex aqueous streams.

## INTRODUCTION

The sustainable supply of critical elements underpins the global transition to low-carbon energy systems (*1*). This challenge is especially evident in high-salinity brines relevant to lithium recovery, as well as in seawater-derived and other chemically complex aqueous systems, where high concentrations of coexisting ions with different valences and hydration characteristics render selective transport intrinsically difficult (*2*). Membrane-based separations that combine high ionic throughput with precise ion discrimination are therefore central to advancing sustainable resource recovery and electrochemical separation processes, particularly in water-constrained and chemically complex environments (*3*). Ion migration through confined pore channels is governed by the interplay between pore free volume and ion-pore interactions, which determine the energetic barriers associated with ion partitioning, dehydration, and diffusion under confinement (*4*). Guided by these principles, substantial efforts have been devoted to engineering synthetic membranes with tailored ion-transport pathways (*5*, *6*). However, achieving high conductivity while effectively excluding multivalent ions under realistic aqueous conditions remains a persistent materials challenge. In particular, the development of transport channels capable of efficient mono/multivalent ion discrimination across both cationic and anionic systems remains an unmet need for next-generation electrochemical separation membranes (*7*, *8*).

Conventional ion exchange membranes, such as perfluorinated Nafion and hydrocarbon-based analogs, typically rely on microphase separation of hydrophilic ionic domains within a hydrophobic matrix to achieve mechanical integrity and dimensional stability (*9*, *10*). While these architectures support efficient ion conduction, the resulting transport channels are typically broad and poorly defined. Upon hydration, their domains often swell to several nanometers, facilitating rapid ion diffusion but severely compromising selectivity (*11*, *12*). As a result, most conventional membranes struggle to deliver simultaneous high conductivity and comprehensive mono/divalent discrimination, limiting their effectiveness in complex, multicomponent aqueous systems (*13*, *14*). To overcome these limitations, porous materials such as porous organic polymers (POPs), metal-organic frameworks (MOFs), covalent organic frameworks (COFs), and porous organic cages have been explored as platforms for angstrom-scale confinement (*15*–*17*). Although angstrom-scale porous materials offer molecular precision, their limited processability, scalable integration, and operational robustness have constrained their sustainability-relevant deployment. Bridging the gap between molecular precision and process-level robustness, therefore, remains a key unsolved problem for sustainable membrane technologies.

Polymers of intrinsic microporosity (PIMs) offer a conceptually distinct strategy for mitigating the long-standing conductivity-selectivity trade-off (*18*, *19*). Unlike phase-separated ion exchange membranes, PIMs have inherent angstrom-scale channels arising from the inefficient packing of rigid, contorted backbones, such as ladder-type Tröger’s base and spirocyclic motifs (*20*, *21*). This architecture combines high free volume with well-defined constrictions, underpinning the widespread use of PIMs in gas separation membranes and, more recently, in proton-conducting separators for electrochemical devices requiring ultrafine pores and precise molecular discrimination (*22*–*24*). However, in aqueous ion separations, these narrowly confined channels often impose excessive transport resistance on hydrated monovalent ions, leading to large transport resistances, limited salt fluxes, and modest process efficiency in desalination and brine treatment (*25*, *26*). Moreover, introducing the high density of fixed charges required for multivalent-ion exclusion often increases water uptake and swelling in linear or semirigid PIMs, dilating pore constrictions and progressively undermining mono- and divalent selectivity under realistic operating conditions (*27*, *28*). Therefore, a viable PIM-derived membrane for stringent aqueous ion separations cannot rely on intrinsic microporosity alone; it must also preserve pore size stability under hydration, maintain continuous ion conduction pathways, and encode robust ion-pore interactions within a mechanically rigid framework (*29*). This need motivates the extension of the intrinsic microporosity concept to covalently crosslinked porous polymer networks with enhanced structural rigidity and programmable ionic microenvironments.

Here, we address a central sustainability bottleneck in electrochemical separations: the lack of membrane platforms that simultaneously deliver high ionic flux, sharp mono/divalent selectivity, and long-term stability under realistic aqueous conditions relevant to resource recovery. In particular, for lithium extraction from brines and NaCl separation from seawater, pressure-driven nanofiltration membranes are among the most widely studied and practically relevant technologies (*30*). However, recent assessments have emphasized that nanofiltration-based lithium extraction, while effective for Li^+^/Mg^2+^ separation, may face limitations in simultaneously reducing the Mg^2+^/Li^+^ ratio and achieving substantial Li^+^ enrichment under high-salinity conditions. By contrast, electrodialysis can, in principle, couple electrically driven ion separation with ion concentration, although its performance depends strongly on membrane permselectivity, current efficiency, and system configuration (*31*, *32*). To this end, we report a new class of Tröger’s base (TB)–derived POP membranes that are fundamentally distinct from conventional ion-exchange membranes designed for strict cation-only or anion-only permselectivity. Instead of relying primarily on ion-exchange domains in a dense polymer matrix, these membranes integrate the continuous microporosity characteristic of PIMs with the structural rigidity and pore uniformity of covalently crosslinked networks, thereby creating a hydration channel–regulated platform for mono/multivalent ion discrimination across both cationic and anionic species.

These membranes are synthesized through acid-promoted copolymerization of twisted 3,3′-dimethylbiphenyl-4,4′-diamine (DMBP) units and triamine 1,3,5-tris(4-aminophenyl) benzene (TPB) units, yielding a three-dimensional microporous framework with tunable topologies (Fig. 1, A to C). The subsequent quaternization of the ladder-like Tröger’s base bridges generates well-interconnected angstrom-scale ion channels in which pore architecture and local chemistries are simultaneously engineered (Fig. 1, D to G). This molecular design imparts three key features: (i) a rigid, covalently crosslinked backbone that suppresses hydration-induced swelling (with a swelling ratio 1.52%) and maintains long-term dimensional stability; (ii) conformationally engineered channels that assemble into continuous hydration pathways, enabling water molecules to diffuse with effective mobilities (4.35 × 10^−10^ m^2^·s^−1^), and thereby boosting monovalent-ion conduction; and (iii) near-complete exclusion of comprehensive multivalent ions—including Mg^2+^, Al^3+^, SO_4_^2−^, and PO_4_^3−^—through the synergistic effects of steric sieving, Donnan exclusion, and electrostatic trapping (*33*, *34*) (Fig. 1, D and G). As a result, exceptional selectivities are achieved, reaching 43 for K^+^/Mg^2+^ (304 for K^+^/Al^3+^) and 151 for Cl^−^/SO_4_^2−^ (833 for Cl^−^/PO_4_^3−^) under multi-ion electrodialysis conditions. By translating molecular-scale pore and charge engineering into robust device-level performance, these membranes enable direct lithium enrichment from high-Mg brines and direct NaCl extraction from seawater. In a three-stage electrodialysis process, ∼500-fold lithium enrichment was achieved without conventional Mg/Ca preprecipitation, and the resulting Li-enriched stream was subsequently converted into crystalline Li_2_CO_3_ with 99.5% purity. Meanwhile, the membrane facilitated the direct extraction of NaCl from seawater with 99.4% purity. Overall, this work demonstrates how membrane molecular design can deliver system-level sustainability gains, including reduced energy demand, lower chemical inputs, and improved material durability in critical resource-recovery processes.

**Fig. 1.**
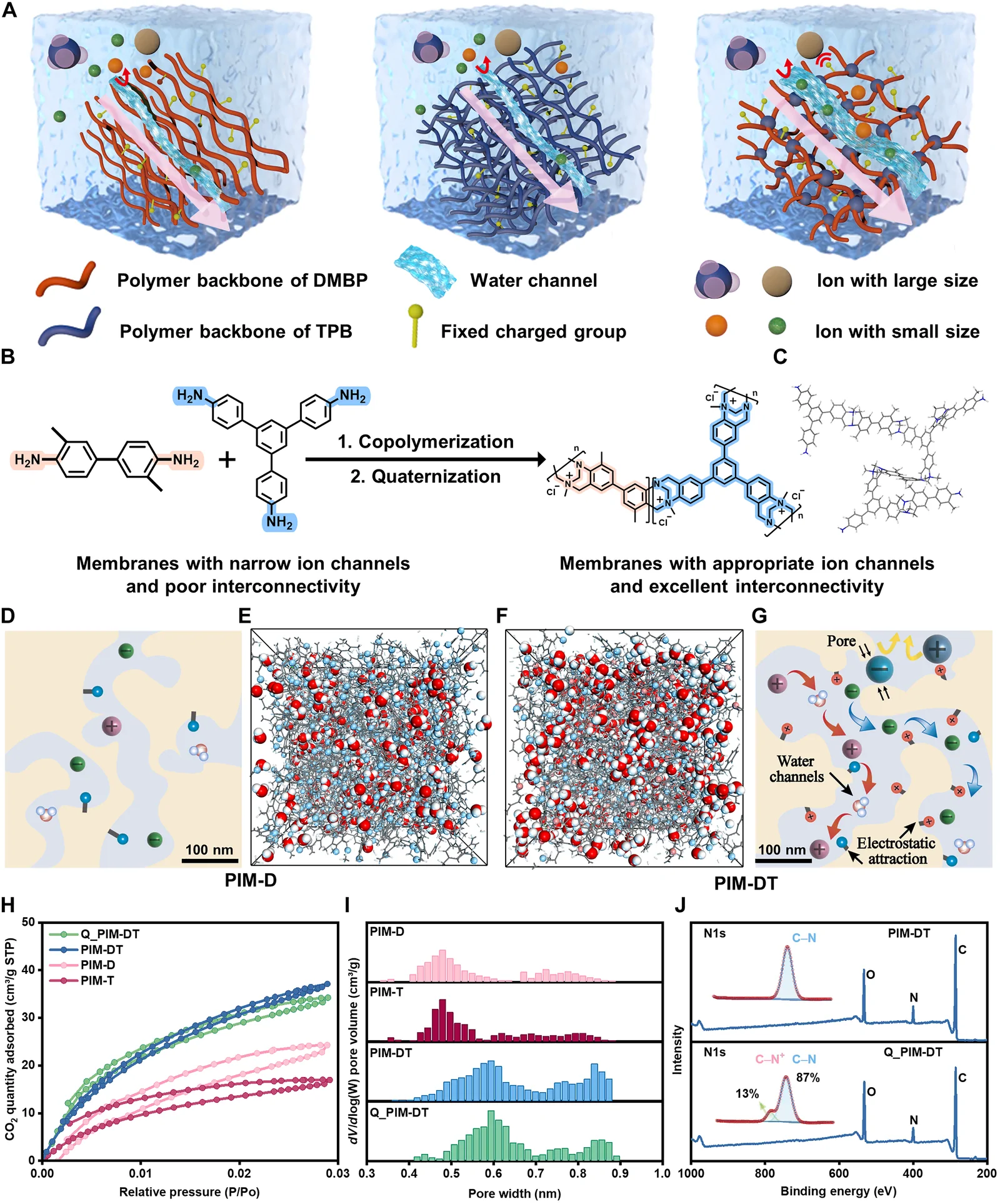
Design, structure, and characterization of TB-based POP membranes. (**A**) Schematic comparison of uncontrolled, overly narrow ion channels formed by molecular chains of PIM-D (left) and PIM-T (middle) versus size-tailored microporous ion channels in the PIM-DT network (right). (**B**) Synthetic route for PIM-DT via acid-promoted condensation-cyclization reaction. (**C**) Representative polymer chain segments of the PIM-DT membranes. (**D**) Schematic illustration of the ion-channel morphology in pristine PIM-D membranes. (**E** and **F**) Simulated amorphous cells of PIM-D (E) and PIM-DT (F), showing intrinsic free volume distributions; water molecules are shown as spheres (oxygen, red; hydrogen, white), nitrogen atoms in light blue, and quaternized nitrogen (N^+^) species in pink. (**G**) Schematic representation of abundant, interconnected angstrom-scale ion channels in Q_PIM-DT membranes, enabling rapid and selective ion transport. (**H**) CO_2_ adsorption isotherms at 273 K for PIM-D, PIM-T, PIM-DT, and Q_PIM-DT. (**I**) Pore size distributions of PIM-DT membranes derived from CO_2_ sorption isotherms using DFT calculations. (**J**) Full XPS spectra of PIM-DT and Q_PIM-DT and corresponding N_1s_ core-level spectra before and after quaternization treatment.

## RESULTS

PIM chemistry provides access to a wide range of microporous materials by exploiting the structural diversity of rigid, contorted monomers, enabling the construction of tailored pore environments and POP-like crosslinked networks. In this work, TB-based POP membranes were synthesized through a solution-processable, acid-promoted condensation-cyclization reaction. The sterically hindered triamine 1,3,5-tris(4-aminophenyl) benzene (TPB) serves as a multifunctional crosslinking site, while the linear diamine DMBP controls the spacing between junctions, together acting as complementary building blocks for network formation (*35*). This molecular design produces permanent microporosity, with the framework topology systematically tunable through the triamine-diamine composition. By varying the DMBP/TPB monomer ratios, a series of network POP membranes—denoted as PIM-DT_X (X = 5, 10, 25, and 50) —was prepared, where X represents the molar fraction of TPB incorporated into the polymer backbone (fig. S2). To investigate the influence of ionic functionalities on ion separation, the tertiary amine bridges were subsequently quaternized via methylation using CH_3_I, resulting in quaternized membranes (Q_PIM-DT; fig. S3). Notably, both DMBP and TPB are low–molecular weight monomers (*MW* < 500 Da), with excellent solubility in common polar aprotic solvents such as dimethyl sulfoxide (DMSO). This high solubility facilitates efficient solution-phase copolymerization into extended POP networks, enabling straightforward fabrication of defect-free, self-standing membranes by conventional solvent-casting techniques. Mechanical testing (fig. S4) demonstrates that the Q_PIM-DT membranes have robust mechanical properties, achieving a tensile strength of 40.8 MPa and an elongation at break of 14.6%. Thermogravimetric analysis (fig. S5) further confirms their thermal stability, with primary decomposition temperatures starting above 300°C, indicating suitability for electrochemical operation under demanding conditions.

The intrinsic free volume of the TB-based POP membranes plays a crucial role in defining pore channel formation and ion-selective transport. To characterize the microporous architecture, CO_2_ and N_2_ adsorption measurements were conducted (Fig. 1H and fig. S6). CO_2_ sorption isotherms reveal that uptake capacities of 8.61, 11.43, 10.52, and 7.18 cm^3^·g^−1^ STP for pristine DMBP, PIM-DT, Q_PIM-DT, and pristine TPB, respectively. Notably, both PIM-DT and Q_PIM-DT exhibit nearly identical pore size distributions centered in the range of 0.5 to 0.7 nm (Fig. 1I), which are larger than those of the pristine DMBP and TPB membranes, as derived from density functional theory (DFT) analysis of CO_2_ adsorption isotherms. The enhanced CO_2_ and N_2_ uptake observed in the copolymerized membranes reflects an increase in internal free volume, indicating that the PIM-DT copolymerization reorganizes the polymer framework into a more open and interconnected microporous network. These enlarged, uniformly distributed angstrom-scale voids provide a solid structural basis for creating continuous ion-transport channels essential for selective monovalent ion conduction.

The chemical structures of the polymers were confirmed using Fourier transform infrared (FTIR) spectroscopy (fig. S7) and x-ray photoelectron spectroscopy (XPS; fig. S8) (*36*, *37*). The ion-exchange capacity (IEC), which reflects the number of charged functional groups (quaternary ammonium groups) in the Q_PIM-DT membrane, was measured at 1.21 mmol·g^−1^—considerably higher than that of the unquaternized PIM-DT precursor (0.98 mmol·g^−1^). XPS analysis (Fig. 1J) further indicates that ∼13% of the accessible tertiary amine sites were successfully converted to quaternary ammonium groups during methylation. In addition, the IEC increases systematically with higher TPB incorporation (fig. S9), attributed to the higher density of amine functionalities introduced by the triamine nodes, in agreement with trends observed in the zeta potential measurements (fig. S10). Morphological studies using scanning electron microscopy (SEM) and atomic force microscopy (AFM) confirm that the membranes are dense, defect-free films (fig. S11) and have relatively smooth surface topographies (fig. S12). The membrane thickness ranges from 80 to 90 μm (fig. S13). Overall, these results demonstrate that solution-processable copolymerization, followed by controlled quaternization preserves the structural integrity of the TB-based POP membranes while introducing well-dispersed ionic clusters and a highly interconnected microporous architecture.

To elucidate the ion-screening mechanism and clarify the role of copolymerization in TB-based POP membranes, current-voltage (*I*-*V*) measurements were conducted using mixed electrolyte systems (NaCl/Na_2_SO_4_ and K_2_SO_4_/MgSO_4_), enabling direct comparison of ion transport for anion and cation species with different valences (figs. S15 and S16). The resulting *I*-*V* curves reveal a pronounced composition-dependent transport behavior for monovalent ions. Specifically, the effective conductivities of K^+^ and Cl^−^ increase markedly with TPB incorporation, reaching maxima of 2.53 mS cm^−1^ (K^+^) and 3.85 mS cm^−1^ (Cl^−^) at Q_PIM-DT. In contrast, the conductivities of multivalent ions remain consistently low and largely insensitive to copolymer composition, stabilizing ∼0.83 mS cm^−1^ for Mg^2+^ and 0.95 mS cm^−1^ for SO_4_^2−^ across all membranes tested. Temperature-dependent *I*-*V* measurements further support this selective transport behavior (figs. S17 and S18). The activation energies for monovalent ion transport are minimized at intermediate TPB content, with values of 18.71 kJ mol^−1^ for K^+^ and 17.12 kJ mol^−1^ for Cl^−^ at ∼10 mol % TPB incorporation. These values indicate that moderate copolymerization optimally tunes the effective pore dimensions to closely match the hydrated diameters of K^+^ and Cl^−^ (6.62 and 6.64 Å, respectively) (*38*). In contrast, Mg^2+^ and SO_4_^2−^ exhibited persistently high activation energies regardless of TPB content, reflecting their restricted movement within subnanometer channels. Notably, quaternized Q_PIM-DT membranes exhibited nearly identical composition-dependent trends, differing only in absolute conductivity values, indicating that backbone topology—rather than cation functionalization—dominates size-selective ion transport.

To further correlate polymer topology with ion-transport behavior, molecular dynamics (MD) simulations were used to analyze the effect of ternary-site copolymerization on free volume characteristics. The fractional free volume (FFV) exhibits a clear nonmonotonic dependence on TPB content, reaching a maximum at PIM-DT (Fig. 2C). This behavior indicates that moderate incorporation of the triamine node disrupts dense chain packing and generates a higher density of interconnected micropores. In contrast, pristine PIM-D and PIM-T membranes exhibit more compact chain packing, resulting in smaller voids and reduced porosity (Fig. 2, A and B). Pore size distribution analysis further confirmed this conclusion: PIM-DT contains the highest fraction of angstrom-scale pores in the 6 ∼ 7 Å diameter range, corresponding to cavity regions with radii of ∼3 Å (fig. S19), consistent with its experimentally observed high conductivity and low activation energies (fig. S18). In comparison, pristine PIM-D and PIM-T mainly feature sub-5 Å pores, which impose substantial steric constraints even for hydrated monovalent ions. To further evaluate pore accessibility and connectivity, void percolation was measured using spherical probes with radii of 0.8, 0.9, 1.0, and 1.2 Å (Fig. 2, D to F). In both PIM-T and PIM-D (Fig. 2, D and E), only the smallest probe (0.8 Å) yields a minimally continuous void network, while connectivity deteriorates sharply as probe size increases. At a probe radius of 1.0 Å, voids remain present but no longer form a continuous percolated network across the simulation cell. In striking contrast, PIM-DT maintains interconnected void pathways even for the larger probes (Fig. 2F and fig. S20), demonstrating that moderate PIM-DT copolymerization significantly enhances three-dimensional pore connectivity. Together, the connectivity and pore size distribution results suggest a structural feature comprising relatively larger cavity domains (∼3 Å in radius) interconnected by much narrower bottlenecks on the order of ∼1 Å. Such a confined topology favors monovalent ion transport by enabling partial dehydration at the bottlenecks and rehydration within the larger cavity domains.

**Fig. 2.**
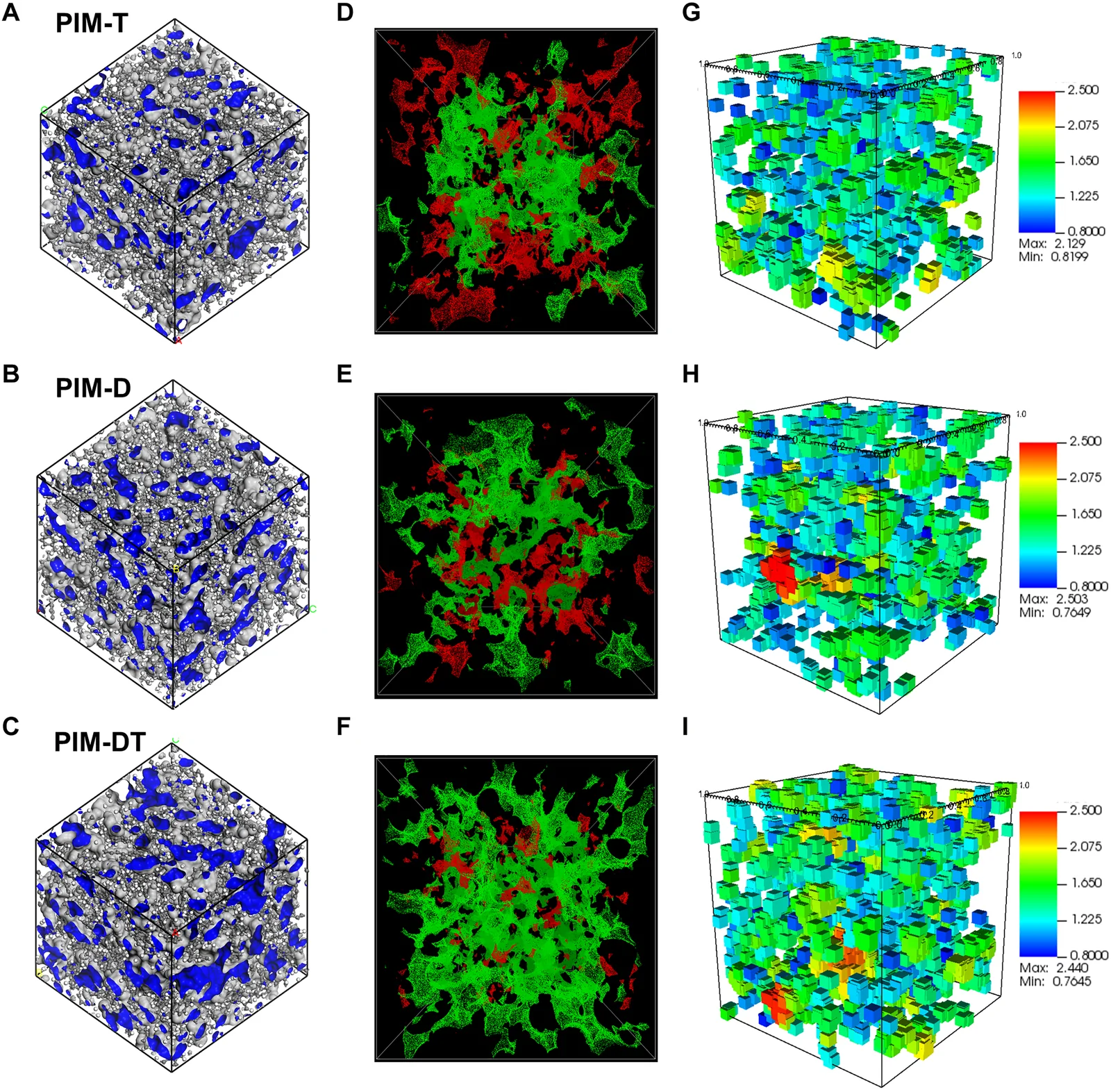
Structural analysis of TB-based POP network models. (**A** to **C**) Simulated 3D amorphous cells of the PIM-T (A), PIM-D (B), and PIM-DT (C) networks. Blue and gray regions represent intrinsic voids within the polymer skeleton. (**D** to **F**) Interconnected (green) and disconnected (red) void networks identified using a spherical probe with a radius of 0.9 Å in PIM-T (D), PIM-D (E), and PIM-DT (F). (**G** to **I**) Void distributions color-coded by pore diameter for PIM-T (G), PIM-D (H), and PIM-DT (I), highlighting the enhanced pore size and connectivity in the copolymerized network.

The visualization of the void networks further corroborates these findings (Fig. 2, G to I). When available voids are mapped according to the maximum probe diameter they can accommodate, PIM-DT exhibits larger and more continuous pore domains (Fig. 2I) than either pristine component (Fig. 2, G to H). The quaternized Q_PIM-DT displays nearly identical FFV values, pore size distributions, and connectivity profiles to its unquaternized PIM-DT counterpart (fig. S20), confirming that quaternization introduces localized ionic clusters without disrupting the intrinsic free-volume architecture. Collectively, these results demonstrate that ∼10 mol % TPB incorporation optimally balances chain rigidity and free-volume generation, yielding the highest porosity and pore interconnectivity while preserving a stable three-dimensional microporous network (figs. S21 and S22).

Small-angle x-ray scattering (SAXS) measurements reveal further insight into mesoscale structural organization and hydration behavior. A distinct primary scattering peak emerges upon quaternization, corresponding to a characteristic interdomain spacing (*d*) associated with ionic-rich hydrophilic regions. Using Bragg’s law (*39*), the d-spacing is determined to be 18.22 nm for PIM-DT and increases to 19.71 nm for Q_PIM-DT (Fig. 3F and fig. S23). This expansion reflects enhanced ionic clustering and a more pronounced microphase-separated structure in the quaternized membranes, in contrast to the relatively featureless SAXS profile of the unquaternized PIM-DT membrane (Fig. 3, A and B). Molecular simulations of hydrated Q_PIM-DT reveal that quaternary ammonium groups aggregate into localized ionic domains that serve as nucleation sites for water accumulation, leading to the formation of continuous water-rich regions throughout the polymer matrix (*40*). These regions are interconnected by dense hydrogen-bond networks, forming angstrom-scale internal hydration channels that facilitate efficient ion transport (figs. S24 and S25). By comparison, PIM-DT without quaternization exhibits only isolated water pockets with poor connectivity, preventing the establishment of continuous ion-transport channels (Fig. 3, D and E).

**Fig. 3.**
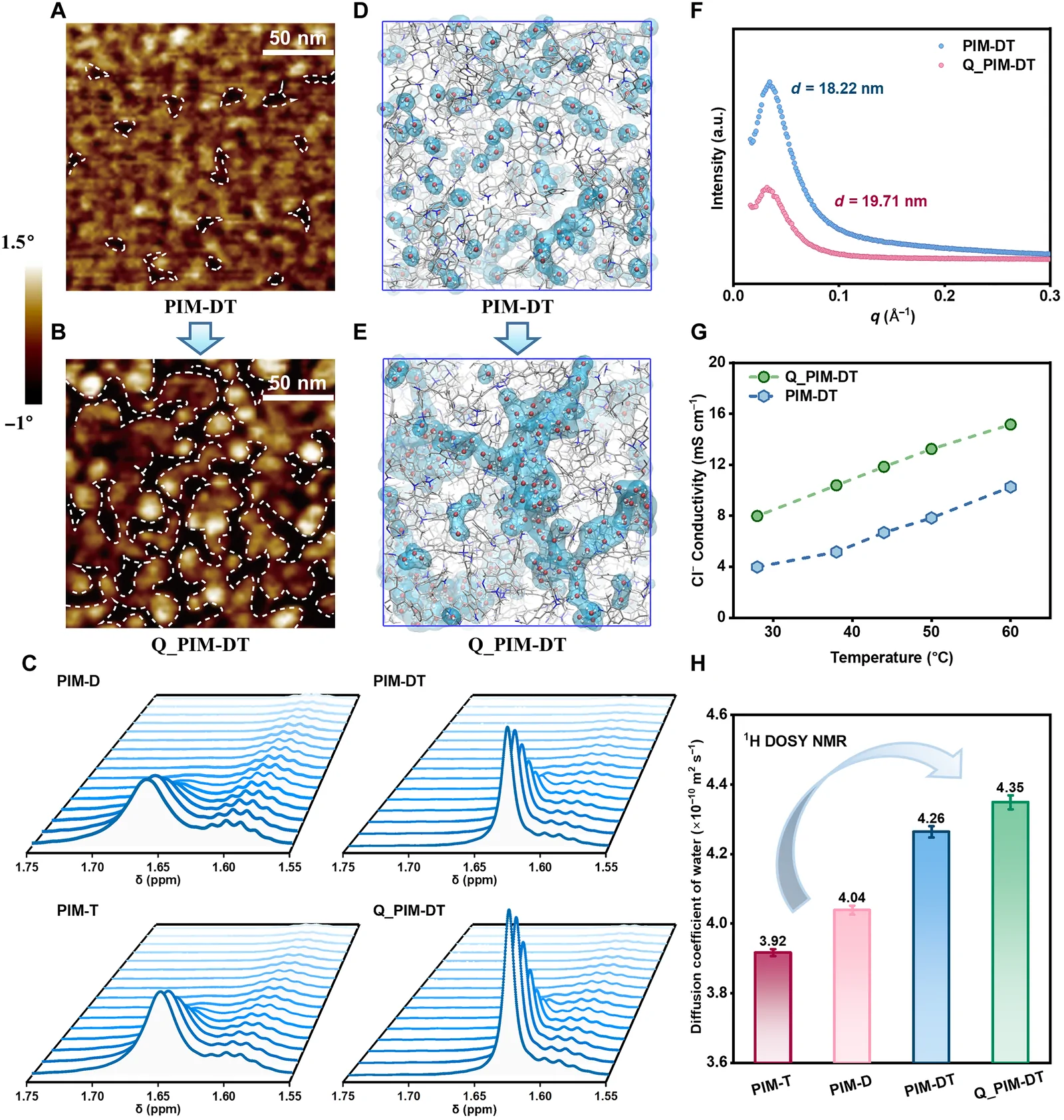
Formation of ionic domains and continuous hydration pathways in TB-based POP membranes. AFM phase images of (**A**) PIM-DT and (**B**) Q_PIM-DT. (**C**) ^1^H diffusion-ordered spectroscopy (DOSY) NMR spectra of PIM-D, PIM-T, PIM-DT, and Q_PIM-DT membranes. Simulated amorphous structures of (**D**) PIM-DT and (**E**) Q_PIM-DT under hydrated conditions, where water molecules are rendered as aggregated clusters to visualize the connectivity of internal hydration networks. (**F**) SAXS profiles of PIM-DT and Q_PIM-DT membranes. (**G**) Temperature-dependent chloride (Cl^−^) conductivity of PIM-DT and Q_PIM-DT membranes. (**H**) Water diffusion coefficients extracted from the ^1^H DOSY NMR spectra.

The impact of these structural and hydration features on water dynamics was quantified by diffusion-ordered spectroscopy (DOSY) ^1^H nuclear magnetic resonance (NMR) spectroscopy (Fig. 3C). Pristine DMBP and TPB membranes exhibit relatively low water self-diffusion coefficients (<4.0 × 10^−10^ m^2^·s^−1^), consistent with compact chain packing and restricted free volume. In contrast, moderate copolymerization followed by quaternization leads to a pronounced increase in water mobility, with Q_PIM-DT exhibiting a self-diffusion coefficient of 4.35 × 10^−10^ m^2^·s^−1^ (Fig. 3H and fig. S26), the highest among the compositions examined in this study. This value is notable for an angstrom-scale microporous network and is comparable to those reported for representative state-of-the-art ion-exchange membranes (*41*, *42*). Consistent with these findings, chloride conductivity measurements highlight the decisive role of quaternization in stabilizing hydrated ion-transport pathways (Fig. 3G). Although Q_PIM-DT and PIM-DT have similar overall pore structures, the quaternized membranes exhibit substantially higher Cl^−^ conductivity, reaching 8 mS·cm^−1^ at 28 °C and 15 mS·cm^−1^ at 80 °C—corresponding to roughly a twofold enhancement over their nonquaternized analog.

Together, the SAXS, molecular simulation, NMR, and conductivity results establish a coherent structure-hydration-transport relationship, wherein optimized copolymer topology and ionic functionalization cooperatively generate continuous hydrogen-bonded hydration networks that enable rapid and selective monovalent-ion conduction through the polymer matrix.

Given the pronounced suppression of multivalent ion transport in the Q_PIM-DT_10 (hereafter referred to as Q_PIM-DT) membranes, its potential for selective ion separation was systematically evaluated. Ion transport behavior was first examined using *I*-*V* measurements with a range of electrolytes, including monovalent and multivalent cations (K^+^, Na^+^, Li^+^, Mg^2+^, and Al^3+^) and anions (Cl^−^, F^−^, IO_3_^−^, SO_4_^2−^, and PO_4_^3−^). The resulting *I*-*V* curves demonstrate that Q_PIM-DT supports efficient conduction of monovalent salts while strongly suppressing transport of divalent and trivalent ions. For chloride-based cation electrolytes, KCl exhibits the highest conductivity, with conductance following the order *G*_K_^+^ > *G*_Na_^+^ > *G*_Li_^+^ ≫ *G*_Mg_^2+^ > *G*_Al_^3+^ (Fig. 4A). At a concentration of 0.5 M, the selectivity ratios reach 10.0 for K^+^/Mg^2+^ and 28.6 for K^+^/Al^3+^ (Fig. 4C). A comparable trend is observed for sodium-based anion electrolytes (Fig. 4B): Monovalent anions (Cl^−^, F^−^, IO_3_^−^) exhibit substantially higher conductivities than multivalent anions (SO_4_^2−^ and PO_4_^3−^), with the disparity increasing with ionic charge and hydrated radius. At 0.5 M, the Cl^−^/SO_4_^2−^ and Cl^−^/PO_4_^3−^ reach 14.0 and 31.4, respectively, highlighting the membrane’s ability to block bulky, highly hydrated multivalent anions (Fig. 4C). Collectively, these results confirm that Q_PIM-DT forms highly charge-sensitive transport pathways that permit rapid transport of monovalent ions while imposing severe steric and electrostatic penalties on multivalent species.

**Fig. 4.**
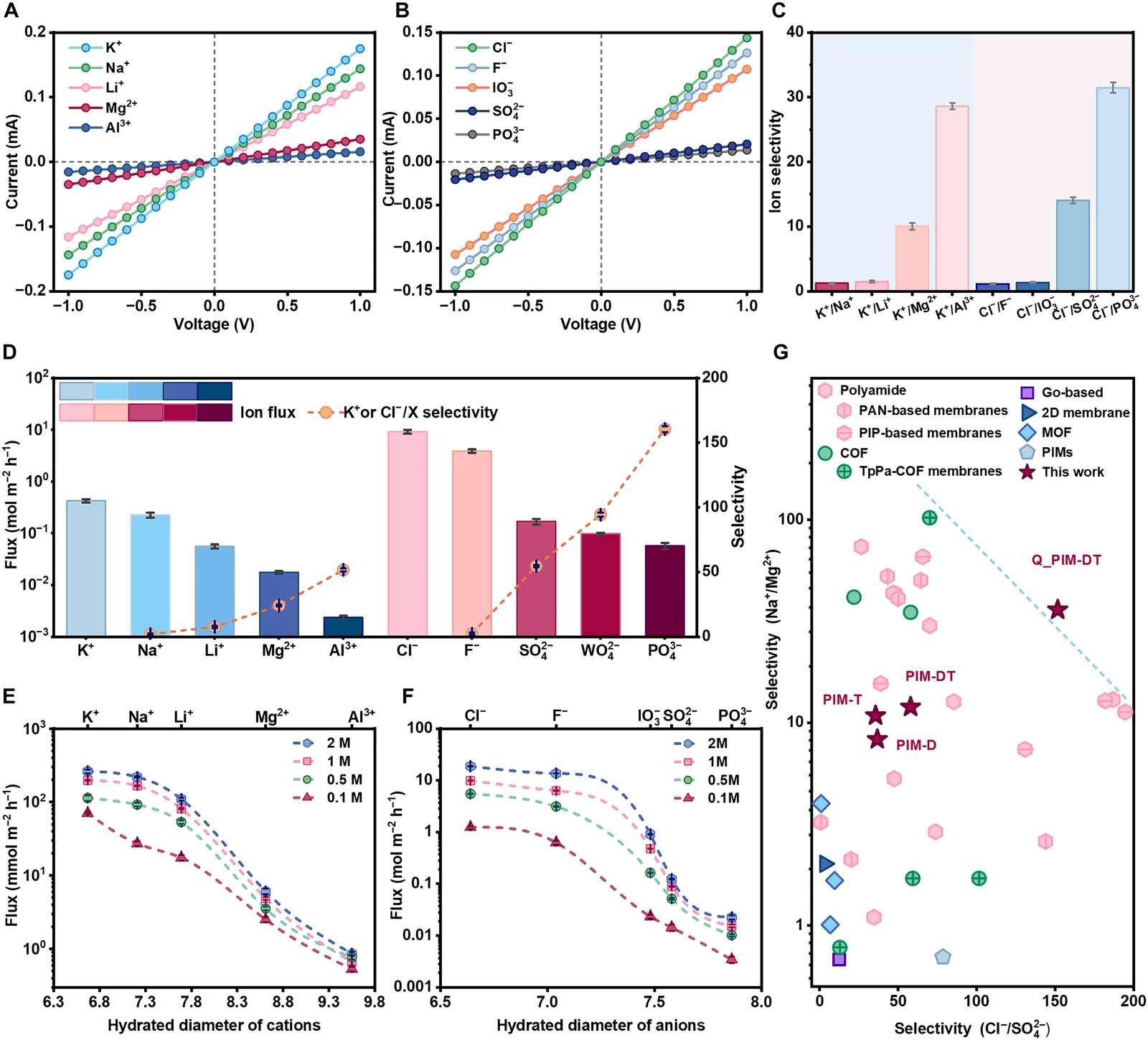
Comprehensive mono/multivalent ion discrimination in Q_PIM-DT membranes. (**A** and **B**) *I*-*V* characteristics of Q_PIM-DT measured in 0.5 M chloride-based (A) and sodium-based (B) electrolytes, revealing distinct transport behaviors across monovalent and multivalent ions. (**C**) Ion selectivity derived from the *I*-*V* measurements. (**D**) Single-salt ion fluxes and corresponding selectivity ratios measured under 0.1 M conditions for individual electrolytes. (**E** and **F**) Ion fluxes under mixed-salt conditions as a function of total ionic strength (0.1 to 2 M), demonstrating concentration-dependent mono/multivalent ion separation. (**G**) Performance comparison of Q_PIM-DT with representative state-of-the-art ion-selective membranes reported in the literature (*54*–*80*). ppm, parts per million.

To further quantify ion discrimination, transmembrane fluxes of salts were measured at 0.1 M (Fig. 4D). Monovalent ions such as K^+^ and F^−^ exhibit the highest fluxes, whereas divalent ions (Mg^2+^ and SO_4_^2−^) experience nearly one order of magnitude reduction, and trivalent ions (Al^3+^ and PO_4_^3−^) are almost completely rejected. Consequently, selectivity ratios reach 24.1 for K^+^/Mg^2+^ and 51.6 for K^+^/Al^3+^. Similarly, Cl^−^/SO_4_^2−^ and Cl^−^/PO_4_^3−^ selectivities reach 54.6 and 160.5, respectively, confirming the effective exclusion of multivalent anions. In contrast, ions with identical valence but different sizes (e.g., K^+^/Na^+^ and Cl^−^/F^−^) display nearly identical fluxes (ratios ≈ 1), reflecting the membrane’s intrinsically high conductivity toward comprehensively monovalent ions.

The permselectivity of Q_PIM-DT was further systematically examined using multi-ion mixtures over a wide concentration range (0.1 to 2 M) at a current density of 5 mA·cm^−2^ (Fig. 4, E and F). Across all conditions, ion permeation correlates strongly with ionic valence.

1) Cations: Selectivity ratios such as K^+^/Mg^2+^ increase steadily with concentration, rising from ∼28 at 0.1 M to ∼43 at 2 M, while the rate of increase gradually diminishes at higher salinities. At low concentration, monovalent cations (K^+^, Na^+^) migrate efficiently through continuous hydration networks, while multivalent cations (Mg^2+^, Al^3+^) are strongly hindered by severe confinement-induced dehydration and stringent charge compensation requirements. As salinity increases, enhanced counterion screening partially migrates long-range electrostatic repulsion for multivalent cations; however, their conduction remains fundamentally limited by large dehydration barriers. Meanwhile, progressive saturation of the hydration channels, increased ion-ion interactions (i.e., ionic crowding/ionic pairing), and slower hydration-shell recovery under high-salinity conditions increasingly restrict monovalent-cation mobility. Consequently, monovalent/divalent selectivity continues to rise with concentration but approaches a plateau at high ionic strength. Notably, trivalent cations remain exclusively excluded across the entire concentration range, with K^+^/Al^3+^ selectivities exceeding 150.

2) Anions: In contrast to cations, anion selectivity ratios of Cl^−^/SO_4_^2−^ increased more rapidly with concentration, rising from ∼86 at 0.1 M to ∼151 at 2 M. Beyond steric constraints imposed by hydrated size, multivalent anions (SO_4_^2−^ and PO_4_^3−^) exhibit strong electrostatic binding to the quaternary ammonium sites and show slow exchange dynamics within the confined, low-dielectric membrane matrix. With increasing salinity, preferential association of multivalent anions becomes more pronounced, leading to ionic trapping near fixed cationic sites—consistent with Manning-type condensation behavior—which further suppresses their mobility. By contrast, monovalent anions remain weakly associated and dynamically exchangeable, rendering their transport comparatively less sensitive to increased ionic strength. A similar concentration-accelerated enhancement in selectivity is observed for Cl^−^/PO_4_^3−^.

This dual transport behavior confirms that quaternized TB-based POP membranes form conformationally engineered, charge-decorated channels that simultaneously allow rapid transport of monovalent ions and near-complete exclusion of multivalent ions under practical operating conditions. Although other PIM-DT variants, as well as pristine DMBP and pristine TPB (figs. S27 and S28) membranes, exhibit qualitatively similar concentration-dependent selectivity trends, the magnitude of selectivity is substantially enhanced in Q_PIM-DT. At a high salinity of 2 M, the best-performing Q_PIM-DT achieved high Cl^−^/SO_4_^2−^ and Na^+^/Mg^2+^ selectivities of 151 and 36, respectively, placing it among the top-performing ion-selective membranes reported to date for simultaneous mono/multivalent separation of both cations and anions (Fig. 4G). Notably, for Li^+^/Mg^2+^ separation, Q_PIM-DT exhibits competitive performance compared with previously reported PIM-based membranes and superior performance to several commercial CEMs (figs. S29 and S30 and table S3). However, its Li^+^/Mg^2+^ selectivity remains relatively modest compared with highly specialized lithium-ion sieving membranes specifically designed for Li^+^ recognition. This difference further indicates that Q_PIM-DT is primarily designed for hydration-channel-regulated mono/multivalent ion discrimination for both cationic and anionic systems.

To elucidate the origin of ion selectivity in Q_PIM-DT, a representative fragment of the crosslinked polymer network was extracted and treated as the active transport region for nonequilibrium MD (NE-MD) simulations under an external field. Consistent with experimental observations, ion permeation proceeds through three consecutive steps (Fig. 5A): (i) interfacial partitioning, partial dehydration, and confinement-driven pore entry; (ii) migration and diffusion through interconnected angstrom-scale hydration channels; and (iii) rehydration upon exiting the membrane.

**Fig. 5.**
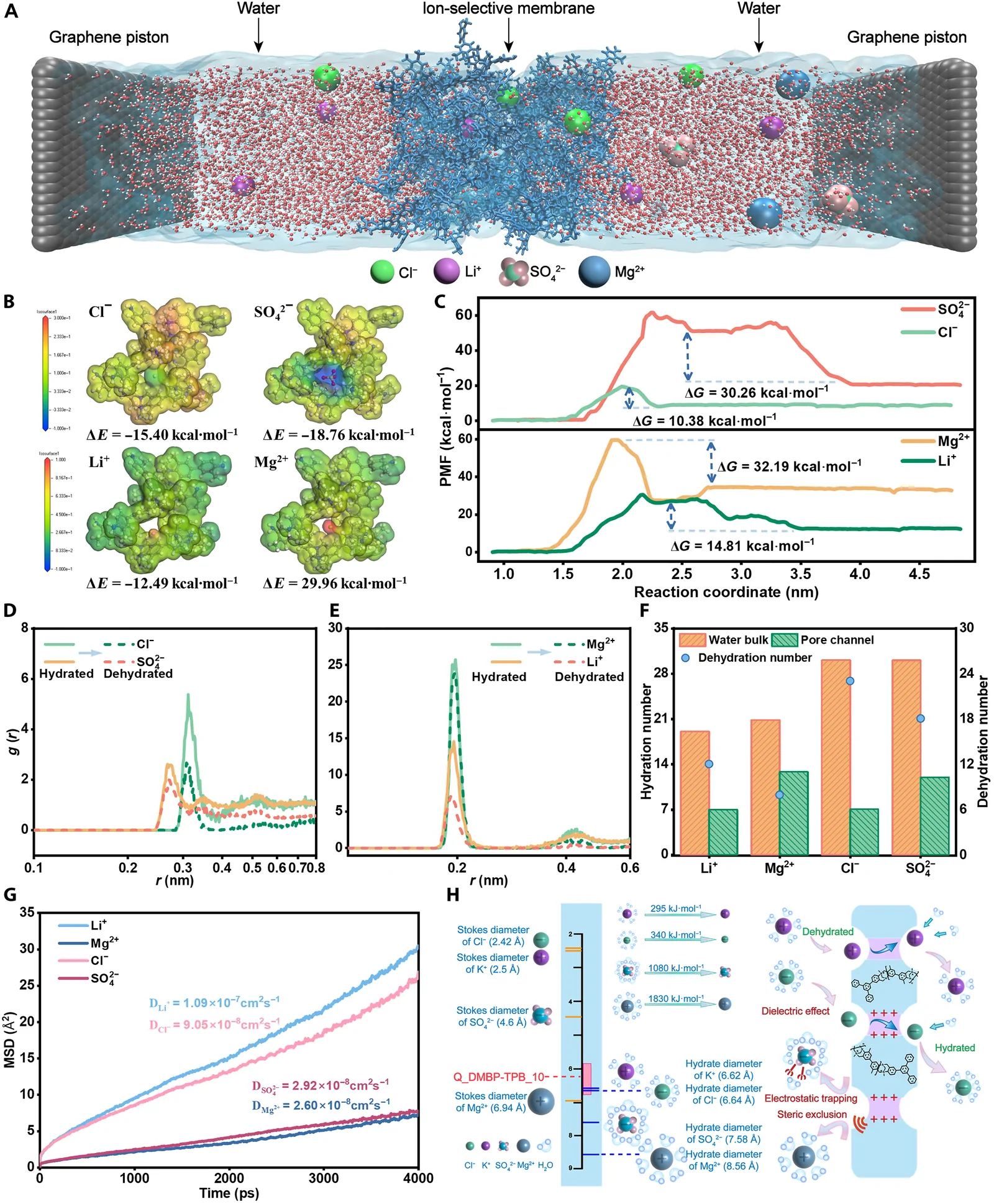
Molecular origin of monovalent ion selectivity in Q_PIM-DT membranes. (**A**) Schematic illustration of the nonequilibrium MD (NE-MD) model for ion transport across the Q_PIM-DT membrane, in which ion migration is driven by an externally applied electric field. (**B** and **C**) Free-energy landscapes derived from potential of mean force (PMF) simulations for Li^+^, Cl^−^, Mg^2+^, and SO_4_^2−^ transport through the polymer matrix of Q_PIM-DT. (**D** and **E**) Continuous radial distribution function (RDF) analysis showing the coordination of water molecules around Li^+^, Cl^−^, Mg^2+^, and SO_4_^2−^ as they traverse the angstrom-scale pore channels of Q_PIM-DT. (**F**) Corresponding dehydration numbers for the first and second hydration shells, calculated from the RDF profiles. (**G**) Mean-squared displacement (MSD) profiles of Li^+^, Cl^−^, Mg^2+^, and SO_4_^2−^ as a function of time within the Q_PIM-DT matrix, reflecting ion-dependent mobility under confinement. (**H**) Schematic illustration of the monovalent/divalent ion separation mechanism, highlighting the coupled effects of hydration energy, dehydration penalty, steric confinement, and electrostatic interactions within angstrom-scale channels.

To quantify the interfacial interactions governing the pore-entry step, NE-MD snapshots corresponding to ions crossing the water-membrane interface were extracted and analyzed using quantum chemical calculations (Fig. 5B). DFT calculations reveal a clear hierarchy in ion-polymer binding energies with the quaternized PIM-DT segment: Δ*E*_bind(Cl^−^) ≈ −15.40 kcal·mol^−1^, Δ*E*_bind(Li^+^) ≈ −12.49 kcal·mol^−1^, Δ*E*_bind(SO_4_^2−^) ≈ −18.76 kcal·mol^−1^, Δ*E*_bind(Mg^2+^) ≈ +29.96 kcal·mol^−1^. Because the Q_PIM-DT framework carries fixed positive charges, Mg^2+^ binding is energetically unfavorable (Δ*E*_bind > 0), indicating that its high charge density cannot be effectively stabilized within the local coordination environment. In contrast, Li^+^ binding is energetically favorable (Δ*E*_bind < 0), demonstrating that tuning of charge distribution and pore geometry allows selective stabilization of Li^+^ while disfavoring Mg^2+^—a critical requirement for efficient Li^+^ extraction from Mg-rich brines.

Electrostatic potential maps further clarify the interfacial selectivity. As Cl^−^ approaches the pore entrance, its local potential around the ion is largely neutralized (green), while adjacent regions of the polymer remain positively charged (red), facilitating electrostatic attraction and rapid entry. By contrast, SO_4_^2−^ retains a strongly negative potential (deep blue) and forms tightly bound, multipoint ion-pair coordination motifs with quaternary ammonium groups at the interface. This leads to pronounced electrostatic trapping of SO_4_^2−^ near the membrane surface, whereas Cl^−^ remains dynamically exchangeable and readily permeable.

To characterize the complete transmembrane energy landscape, potentials of mean force (PMFs) were calculated along the permeate direction for Li^+^, Cl^−^, Mg^2+^, and SO_4_^2−^, averaged over NE-MD trajectories (Fig. 5C). The resulting free-energy barriers for crossing the active layer are Δ*G*_Cl^−^ = 10.38 kcal·mol^−1^, Δ*G*_Li^+^ = 14.81 kcal·mol^−1^, Δ*G*_SO_4_^2−^ = 30.26 kcal·mol^−1^, and Δ*G*_Mg^2+^ = 32.19 kcal·mol^−1^, showing a consistent order: Δ*G*_Mg^2+^ > Δ*G*_SO_4_^2−^ > Δ*G*_Li^+^ > Δ*G*_Cl^−^. Therefore, Mg^2+^ and SO_4_^2−^ encounter barriers approximately two to three times higher than those faced by Li^+^ and Cl^−^. Specifically, for Mg^2+^, strong Donnan repulsion from the cationic framework, combined with steric hindrance at angstrom-scale pore gates, dominates the barrier. For SO_4_^2−^, its large, hydrated size and strong electrostatic association with quaternary ammonium centers significantly increase the migration barrier. In contrast, Li^+^ and Cl^−^ experience substantially lower PMF maxima.

To elucidate the microscopic origin of these PMF barriers, the evolution of the ion solvation environment along the transport pathway was analyzed using ion-water radial distribution functions (RDFs) and the corresponding coordination (hydration) numbers (Fig. 5, D and E). The hydration numbers were extracted from the same externally driven ion-permeation trajectories used for the PMF analysis. Upon entering the angstrom-scale confined channels, all ions exhibit suppressed RDF peaks and reduced coordination numbers, indicating partial dehydration within the pore (Fig. 5F). Li^+^ and Cl^−^ show the largest reductions in hydration number, with dehydration numbers of 12.09 and 23.05, respectively, upon transitioning from the bulk solution into the pore interior. In contrast, Mg^2+^ and SO_4_^2−^ display smaller decreases in hydration number (8.01 and 18.09, respectively). Because of their stronger solvation energies (*43*), Mg^2+^ and SO_4_^2−^ retain more coordinated water molecules inside the pore and therefore maintain a larger effective transport size at the angstrom-scale bottlenecks, resulting in substantially higher free-energy barriers. By contrast, Li^+^ and Cl^−^ can reorganize their hydration shells more readily and adopt a smaller effective transport size, thereby enabling lower-barrier transport through the confined channels. Therefore, the dominant origin of mono/multivalent ion selectivity lies in the confinement-amplified difference in effective hydrated size, arising from their distinct dehydration behaviors. Consistent with this interpretation, the diffusivities derived from mean-squared displacement (MSD) analysis within the pore follow the order *D*_Cl^−^ > *D*_Li^+^ > *D*_SO_4_^2−^ > *D*_Mg^2+^, confirming the strongly suppressed internal mobility of multivalent ions in angstrom-scale channels (Fig. 5G).

In summary, the exceptional ion selectivity of Q_PIM-DT arises from the synergistic interplay of four mechanisms (Fig. 5H), as suggested by the simulations and supported by the experimental conductivity, pore size distribution, and hydrophilicity results: (i) controlled partial dehydration that lowers energy barrier for monovalent ions at the pore entrance; (ii) strong steric confinement and Donnan exclusion that effectively block multivalent cations (such as Mg^2+^) from passing through the angstrom-scale gates; (iii) electrostatic trapping of multivalent anions (such as SO_4_^2−^) at quaternary ammonium clusters, stabilizing interfacial bound states and suppressing forward migration; and (iv) continuous internal hydration channels that enable rapid conduction of comprehensive monovalent carriers. Together, these simulation results, combined with the experimental observations, suggest that charge-sensitive interactions and size-selective confinement collectively establish a distinct transport hierarchy in Q_PIM-DT, enabling fast monovalent-ion conduction while strongly suppressing multivalent-ion transport.

To evaluate the practical separation performance and operational durability of Q_PIM-DT membranes under realistic, high-Mg/Li conditions, a fixed-feed, three-stage electrodialysis process was conducted using a concentrated East Taijiner Salt Lake (initial Mg^2+^/Li^+^ ≈ 40.07), as schematically illustrated in Fig. 6A. The Mg^2+^/Li^+^ ratio in the permeate decreased monotonically with each electrodialysis stage, from 40.07 in the feed to 7.84 after stage I, 0.98 after stage II, and 0.08 after stage III (Fig. 6B and fig. S31). The current efficiencies of the monovalent ions (Li^+^, Na^+^, and K^+^) during the three-stage electrodialysis process were 68.4, 73.6, and 77.5%, respectively (fig. S32). These results further indicate that the observed Li enrichment is mainly due to efficient suppression of Mg^2+^ transport, rather than to targeted Li^+^ recognition. These values correspond to approximately 5-, 8-, and 12-fold decreases per stage, respectively, resulting in an overall reduction of ∼500-fold in the Mg^2+^/Li^+^ ratio across the three-stage process. This pronounced enrichment underscores the strong and sustained exclusion of divalent ion transport afforded by the Q_PIM-DT membrane.

**Fig. 6.**
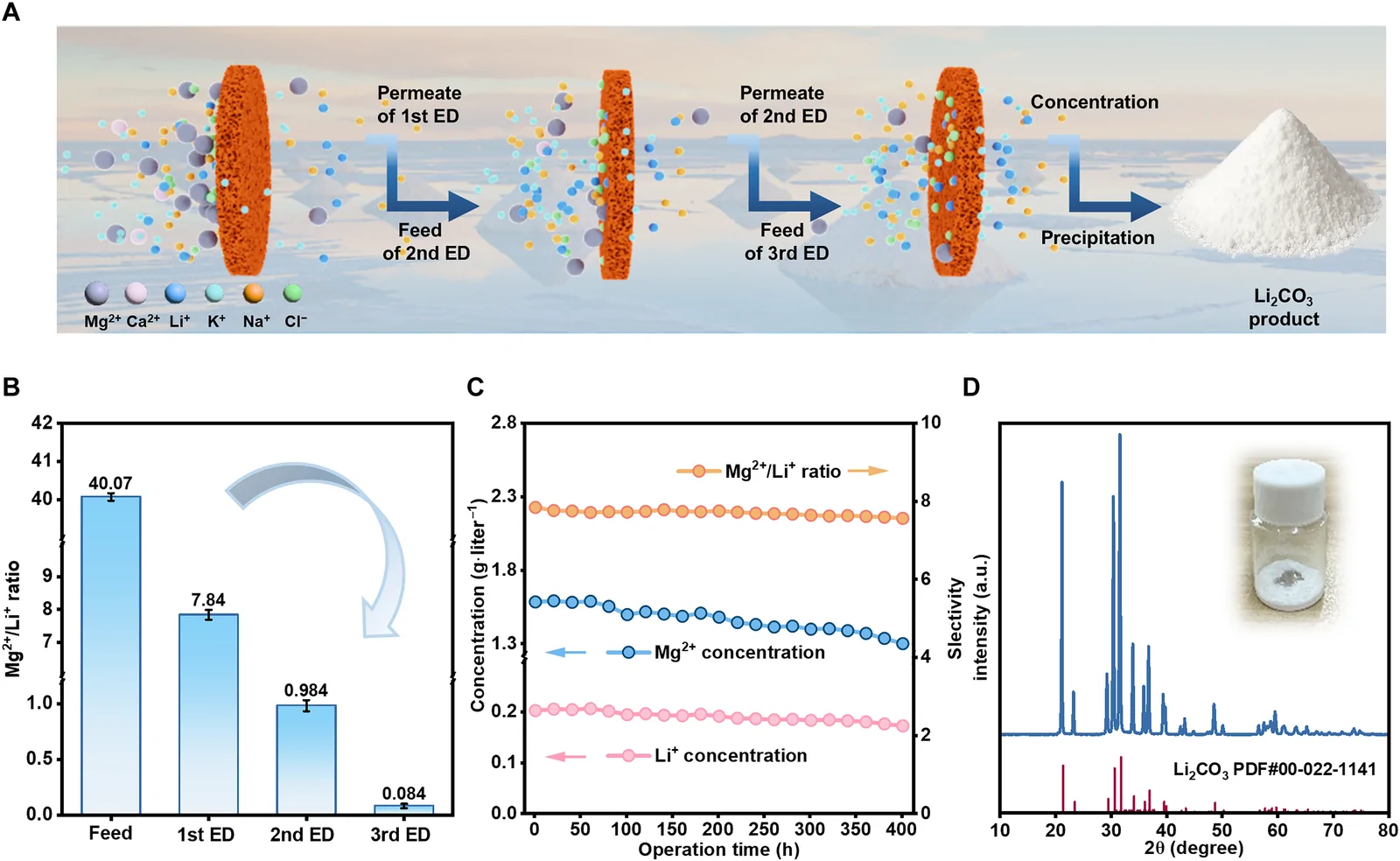
Direct lithium extraction from high-Mg brines enabled by Q_PIM-DT membranes. (**A**) Schematic illustration of a streamlined three-stage electrodialysis process for Li_2_CO_3_ extraction from simulated high-Mg/Li brine. (**B**) Evolution of the Mg^2+^/Li^+^ ratio in the Li-enriched product stream after the first, second, and third electrodialysis stages, demonstrating progressive suppression of divalent ion transport. (**C**) Mg^2+^/Li^+^ ratio and Li^+^ flux of the product stream during prolonged continuous operation of the first-stage electrodialysis, illustrating stable separation performance and operational durability. (**D**) XRD pattern of the recovered Li_2_CO_3_ product, confirming high crystallinity and phase purity. a.u., arbitrary unit.

These observed enrichment behaviors are fully consistent with the transport hierarchy established in earlier sections, including *I*-*V* characteristics (Fig. 4, A and B), activation energy analysis (fig. S18), and flux and concentration dependence (Fig. 4, E and F). Monovalent ions (K^+^/Na^+^/Li^+^/Cl^−^) readily migrate through quaternary ammonium-decorated hydration channels with low energetic barriers, whereas multivalent ions, particularly Mg^2+^, are strongly suppressed by large dehydration penalties and stringent charge compensation constraints imposed by angstrom-scale confinement in the TB-based POP framework. Mechanistically, stage I separation is dominated by Donnan exclusion and steric hindrance, resulting in pronounced Mg^2+^ depletion while maintaining high Li^+^ conductivity. In stages II and III, continued Mg^2+^ exclusion progressively reshapes interfacial ion partitioning. The resulting increase in local Li^+^ concentration and Li^+^/Mg^2+^ ratio lowers the effective free-energy barrier for Li^+^ entry into the nanochannels, thereby establishing Li^+^ as the dominant charge-compensating ion within the confined pore environment. Simultaneously, Cl^−^ transport is increasingly constrained by local electroneutrality and fixed-charge effects, suppressing Cl^−^ back diffusion and further increasing the effective Li^+^ transport number. This cascade-driven reinforcement of Li^+^-selective transport rationalizes the progressively enhanced enrichment factors observed across successive stages.

The long-term stability was further evaluated via continuous electrodialysis operation over 400 hours under constant current conditions. Throughout extended cycling, both Li^+^ flux and Mg^2+^/Li^+^ selectivity remained essentially unchanged, with Mg^2+^ leakage consistently below 1.8 g·liter^−1^ (Fig. 6C). The membrane resistance and voltage profiles showed negligible drift (<3%), indicating stable electrochemical performance. Post-operation SEM, FTIR, and XPS analyses revealed no evidence of delamination, pinhole formation, or chemical degradation (figs. S33 and S34), confirming the mechanical and chemical robustness of the membrane. This durability is attributed to the TPB node–reinforced, covalently crosslinked architecture, which stabilizes the microporous network against hydration-driven rearrangement under sustained electric fields and high ionic strength. Last, the Li-enriched permeate obtained after three-stage electrodialysis was concentrated by evaporation and subsequently treated with Na_2_CO_3_ to precipitate Li_2_CO_3_. At this stage, the Li^+^ and Mg^2+^ concentrations were 5.4 and 0.45 g liter^−1^, respectively (fig. S31), and the low Mg content does not interfere with the precipitation of Li_2_CO_3_. The resulting XRD pattern of the precipitate (Fig. 6D) exhibits sharp diffraction patterns consistent with the reference Li_2_CO_3_ phase, indicating its high crystallinity. Furthermore, the solid product was then redissolved and analyzed by inductively coupled plasma optical emission spectrometry (ICP-OES), confirming a purity of up to 99.5% (fig. S35). Overall, Li recovery from initial brine to final product was 76.8%. Notably, this purity meets the requirements for subsequent downstream recrystallization to achieve battery-grade lithium carbonate (*44*).

To demonstrate the proof-of-concept potential of the Q_PIM-DT membrane for comprehensive mono/multivalent ion discrimination, we evaluated its capability to enrich NaCl from simulated seawater (*45*). The Q_PIM-DT membrane exhibits exceptionally high permeability to NaCl while effectively blocking the transport of divalent ions. During an 8-hour electrically driven ion permeation process, the combined mass fraction of Na^+^ and Cl^−^ in the system increased from 87.2% in the original simulated seawater to 99.4%. Meanwhile, the total mass fraction of Mg^2+^, Ca^2+^, and SO_4_^2−^ decreased markedly from 12.9 to 0.6% (fig. S36 and table S4).

Collectively, these results show that Q_PIM-DT supports stagewise lithium enrichment from realistic high-Mg/Li brines, maintains stable long-term electrodialysis performance, and enables downstream recovery of crystalline Li_2_CO_3_. Beyond lithium extraction, its ability to enrich NaCl from simulated seawater highlights a broader capability for comprehensive mono/multivalent ion discrimination, positioning Q_PIM-DT as a durable platform for resource-selective separations in complex brines.

## DISCUSSION

We present Tröger’s base-derived POP membranes that integrate intrinsic microporosity with rigid, covalently crosslinked network architectures to enable selective and durable ion transport under realistic aqueous and electrochemical conditions. This design addresses a central bottleneck in sustainable ion-separation technologies, where high flux, sharp selectivity, and durability must be achieved simultaneously. The membranes suppress hydration-induced swelling, maintain long-term dimensional stability, and form continuous angstrom-scale hydration channels that render rapid transport of monovalent ions while almost completely excluding multivalent ions through combined steric, Donnan, and electrostatic effects. The resulting charge-sensitive energy landscape supports exceptional mono/divalent selectivity across both cations and anions. In representative electrochemical separation processes, the membranes achieve ∼500-fold lithium enrichment from high-Mg brines via a three-stage electrodialysis process without conventional Mg/Ca preprecipitation and also facilitate direct NaCl recovery from seawater. The resulting Li-enriched stream can be converted into crystalline Li_2_CO_3_ with 99.5% purity, and the extracted NaCl reaches 99.4% purity. More broadly, this work establishes a scalable, all-organic membrane platform in which molecular-level control of pore geometry and charge distribution yields reduced energy use, improved durability, and a lower environmental footprint for sustainable resource recovery.

## MATERIALS AND METHODS

### Materials

TPB (95%), DMBP (98%), trifluoroacetic acid (TFA; 98%), dimethoxymethane (DMM; 98%), and methyl iodide (CH_3_I; 98%) were purchased from TCI (Shanghai) Development Co. Ltd. Inorganic salt potassium chloride (KCl; 99.8%), sodium chloride (NaCl; 99.8%), lithium chloride (LiCl; 99.8%), magnesium chloride hexahydrate (MgCl_2_·6H_2_O; 99.8%), aluminum chloride hexahydrate (AlCl_3_·6H_2_O; 99.8%), sulfate (Na_2_SO_4_; 99.8%), sodium fluoride (NaF; 99.8%), sodium iodate (NaIO_3_; 99.8%), sodium tungstate dihydrate (Na_2_WO_4_·2H_2_O; 99.8%), and sodium phosphate (Na_3_PO_4_; 99.8%) were acquired from Sinopharm Chemical Reagent Co. Ltd. and used without additional purification. Deionized water was used throughout this study. Nafion 117 membranes used in diffusion dialysis were obtained from Chemours Chemical (Shanghai) Co. Ltd. The ACS membranes used in selective electrodialysis were obtained from ASTOM Corporation (Japan).

### Synthesis of PIM-DT

A sequential monomer-addition strategy was used, in which DMBP was first polymerized as the connecting unit, followed by the delayed introduction of TPB as crosslinking sites, enabling controlled evolution from linear segments to a branched network. Short-chain monomer DMBP (0.168 g) was reacted with DMM (0.305 g) for 24 hours, catalyzed by TFA (3.0 ml) in a round-bottomed flask at 0 °C. Subsequently, TPB (0.028 g), serving as a branching linker, was added to the reaction mixture, and the copolymerization proceeded for an additional 12 hours at room temperature to yield the branched copolymer.

### Preparation of Q_PIM-DT membrane

Tröger’s base framework membranes were synthesized via a sol-gel method. The as-prepared viscous solution was poured onto a flat glass substrate and heat-treated at 30°C for 12 hours until completely dried. The dried membranes were immersed in an aqueous 0.1 M KOH solution to remove excess TFA. Branched polymer membranes were obtained by varying the dosage of TPB and DMBP, designated as PIM-DT_X, where “X” denotes the molar percentage ratio of TPB in the copolymer.

The resulting PIM-DT_X membranes were further immersed in a 40 weight % (wt %) methanol-methyl iodide mixture for 6 hours to initiate functional quaternization, with the degree of quaternization adjustable by varying the reaction time. Subsequently, the Q_PIM-DT_X membranes were immersed in a 1 M KCl solution for 24 hours to perform anion exchange and then washed with deionized water to remove residual salts.

### Characterizations

Attenuated total reflection infrared-fourier transform infrared reflection characterization (Thermo Fisher Scientific Nicolet iS20) and XPS characterization (Thermo Scientific K-Alpha) were conducted to investigate the molecular structure and chemical composition of PIM-DT_X membranes. X-ray diffraction patterns were obtained using a Rigaku SmartLab SE diffractometer with Cu radiation. The sample was scanned over a 2θ range of 5° to 90° at a step size of 2°/min. The water contact angle was measured by an angle meter (SL200B, Solon Tech Co. Ltd, China). SEM characterization (ZEISS Gemini SEM 300) and AFM characterization (Bruker Scanasyst Dimension Icon AFM, using a peak force tapping mode in ambient air) were conducted to investigate the surface microstructure. Brunauer-Emmett-Teller measurements (ASAP 2640, Micromeritics 3Flex, USA) were conducted to investigate the surface area and pore distribution of PIM-DT_X membranes. Small/wide-angle x-ray scattering (SAXS/WAXS) data were recorded using a Xenocs Xeuss 2.0 instrument equipped with a Cu Kα x-ray source operated at 50 kV and 0.6 mA in the *q* range of 0 to 10 nm^−1^. DOSY ^1^H NMR experiments were performed at a Larmor frequency of 600 MHz using an NMR spectrometer (Bruker Avance NEO 600) to determine the self-diffusion coefficients of H_2_O within membrane samples immersed in CDCl_3_. The surface charging properties of the PIM-DT_X and Q_PIM-DT_X were examined using an electrokinetic analyzer (SurPass 3, Anton Paar, Australia). The concentrations of cations and SO_4_^2−^ were determined using ICP-OES (ARCOS, SPECTRO, Germany). The concentrations of anions (F^−^, IO_3_^−^, and PO_4_^3−^) were determined using an ion chromatography system (Thermo Scientific Aquion, USA) equipped with a conductivity detector. Cl^−^ concentrations were measured separately by Mohr’s method using AgNO_3_ as the titrant and K_2_CrO_4_ as the indicator.

### Selective-ion transport

The selective transport of mono- and divalent ions across the Q_PIM-DT_X membrane was evaluated using *I*-*V* measurements in a two-compartment H-cell. The compartments were separated by the Q_PIM-DT_X membrane (surface area: 1.8 cm^2^) and filled with 30 ml of electrolyte solution (0.1 mol liter^−1^; NaCl, Na_2_SO_4_, KCl, or MgCl_2_, as specified). Before measurement, the Q_PIM-DT_X membranes were preimmersed in the corresponding electrolyte solution for 4 hours to achieve equilibrium. Salt-bridged Ag/AgCl electrodes were placed in each compartment and connected to an Autolab PGSTAT302N (Metrohm AG), which supplied a direct voltage that swept from −1 to 1 V at a scan rate of 0.01 V s^−1^. The resulting electrical signals were processed using NOVA2 (Metrohm AG) to plot *I*-*V* curves. Transmembrane conductivity was derived by fitting the slope of the *I*-*V* curve with linear least squares regression. Membrane ohmic resistance was calculated by subtracting the filmless resistance from the total ohmic resistance of the cell.

Activation energy (*E*_a_) for ion transport through the membrane was measured using an electrochemical method with electrolyte solutions based on the Arrhenius Eq. 1 (*46*)

$$\text{ln}\left({\mathrm{Gt}}_{\pm }T\right)=\text{ln}\left(B_{\pm }\right)-\frac{E_{a}}{R}\times \frac{1}{T}$$ (1)

where *G* is the overall ion conductivity through the membrane (mS·cm^−1^); *B*_±_ is the preexponential factor for anions or cations; *t*_±_ is the transference number for cations and anions, respectively; *R* is the universal gas constant (8.314 J·mol^−1^·K^−1^), *T* is the absolute temperature (K), and *E*_a_ is the apparent activation energy (kJ·mol^−1^). The *G* value was determined by linear sweep voltammetry on an electrochemical workstation at five temperatures (301.15, 311.15, 317.15, 324.15, and 334.15 K).

Similarly, the voltage was swept from −0.1 to 0.1 V, with both chambers filled with a 10-fold KCl concentration gradient (0.01 M and 0.1 M). The membrane potential was determined from the *x*-axis intercept of the *I*-*V* curve, and the transference number *t*- was calculated using the following Eq. 2 (*47*)

$$E_{m}=\left[\frac{t_{+}}{z_{+}}-\frac{t_{-}}{z_{-}}\right]\left[\frac{\mathrm{kT}}{e}\right]\text{ln}\left[\Delta C\frac{\gamma_{\text{high}}}{\gamma_{\mathrm{low}}}\right]$$ (2)

where *E*_m_ is the zero-current potential, *k* is the Boltzmann constant, *T* is the temperature (K), *e* is the elementary charge, ∆*C* is the ratio of high to low concentrations (∆*C* = 10), and γ_high_ and γ_low_ are the activity coefficients of the high- and low-concentration solutions, respectively.

### Ion permeation measurements

The ion separation capabilities of the Q_PIM-DT_X membranes were determined by measuring the selective transport of cations (K^+^, Na^+^, Li^+^, Mg^2+^, and Al^3+^) and anions (Cl^−^, F^−^, IO^3−^, SO_4_^2−^, and PO_4_^3−^) in a selective electrodialysis process. The electrodialysis cell consisted of four compartments: a concentrate compartment, a dilute compartment, and two electrode compartments. Two ruthenium-coated titanium electrodes were used as the anode and cathode, respectively. Anion separation was performed using a CEM/Q_PIM-DT/CEM configuration (fig. S14A), whereas cation discrimination was carried out using an AEM/Q_PIM-DT/AEM configuration (fig. S14B). For cations, the diluted compartment was filled with 200 ml of a multisalt mixture (KCl, NaCl, LiCl, MgCl_2_, and AlCl_3_ at 0.1 mol·liter^−1^) or a single-salt solution for each cation. Meanwhile, the concentrated compartment contained 200 ml of NH_4_Cl (0.4 mol·liter^−1^). For anion selectivity tests, the diluted compartment contained either a multi-anion mixture with all NaCl, NaF, NaIO_3_, Na_2_SO_4_, and Na_3_PO_4_ at 0.1 mol·liter^−1^ or individual sodium salt solutions. The concentrated compartment was filled with 200 ml of KNO_3_ (0.4 mol·liter^−1^). In both cases, the electrode compartments were filled with 200 ml of Na_2_SO_4_ (0.3 mol·liter^−1^), which served as the electrolyte. The Q_PIM-DT_X membranes, with an effective membrane area of 7.0 cm^2^, were sandwiched between the diluted and concentrated chambers, and the corresponding solutions were circulated inside each compartment at a flow rate of 30 ml·min^−1^. Before measurement, the Q_PIM-DT_X membranes were equilibrated for 30 min to reach steady state. Subsequently, a constant current density of 5 mA·cm^−2^ was applied and maintained for 1 hour.

Three sets of each test were performed, and the ion fluxes across the Q_PIM-DT_X membrane were calculated using Eq. 3 (*48*)

$$J=\frac{\left(C^{t}-C^{0}\right)V}{\mathrm{At}}$$ (3)

where *J* (mol·m^−2^·hour^−1^) is the ion flux during the electrodialysis process. *C*^0^ (mol·liter^−1^) and *C*^t^ (mol·liter^−1^) are the initial and final concentrations in the concentrated compartment before and after electrodialysis, respectively. *V* (ml) is the volume of solution in the concentrated cell. *A* (cm^2^) is the effective area of the membrane. *t* is the experimental time (hour).

The selectivity (S) of the Q_PIM-DT_X membrane could be obtained by applying Eq. 4 (*49*)

$$S=\frac{J_{A}C_{\mathrm{dB}}}{J_{B}C_{\mathrm{dA}}}$$ (4)

where $C_{\mathrm{dA}}$ and $C_{\mathrm{dB}}$ are the initial concentrations of ions in the diluted compartment.

The current efficiency (η) of the electrodialysis process using Q_PIM-DT was determined as Eq. 5 from the ratio of transported ion equivalents to the applied electric charge

$$\eta =\frac{zF\Delta n}{\mathrm{It}}$$ (5)

where *z* is the ionic charge, *F* is the Faraday constant, Δ*n* is the amount of transported ions, *I* is the applied current, and *t* is the operation time.

The ion separation capabilities of the Q_PIM-DT_X membranes were verified by measuring the selective transport of K^+^ over Mg^2+^ (Cl^−^ over SO_4_^2−^) via diffusion dialysis, using a two-compartment cell. The Q_PIM-DT_X membrane, with an effective membrane area of 7.0 cm^2^, was sandwiched between the feed-side and permeate-side chambers. Before measurement, the membrane was equilibrated for 24 hours in the test solution, followed by washing with deionized (DI) water. The feed-side compartment was filled with 120 ml of a mixture containing KCl (0.5 mol·liter^−1^) and MgCl_2_ (0.5 mol·liter^−1^) (and NaCl (0.5 mol·liter^−1^) and Na_2_SO_4_ (0.5 mol·liter^−1^) for anion-selectivity tests), while the permeate-side compartment was filled with 120 ml of DI water. Stirring was conducted with a magnetic stirrer in each compartment to minimize concentration polarization, and diffusion dialysis was performed for 12 hours at room temperature.

Three sets of each test were performed, and the dialysis coefficients were determined according to Eq. 6 (*50*)

$$U=\frac{M}{At\Delta C}$$ (6)

where *M* is the amount of a component in moles transported, *A* is the effective membrane area (7.0 cm^2^), *t* is the experimental time (12 hours), and Δ*C* is the average logarithmic concentration (mol·liter^−1^) between the two compartments, which can be obtained by applying Eq. 7 (*51*)

$$\Delta C=\frac{C_{f}^{0}-\left(C_{f}^{t}-C_{d}^{t}\right)}{\text{ln}\left(\frac{C_{f}^{0}}{C_{f}^{t}-C_{d}^{t}}\right)}$$ (7)

where $C_{f}^{0}$ and $C_{f}^{t}$ are the concentrations in the feed-side compartment at time 0 and t, respectively, and $C_{d}^{t}$ is the concentration in the permeate-side compartment after a certain time (t).

The diffusion selectivity (*S*) could be calculated based on Eq. 8 (*52*)

$$S=\frac{U_{A}}{U_{B}}$$ (8)

### Electro-driven ion separation test

Electro-driven separation tests were conducted at an input voltage of −1 V using an H-type diffusion cell. The feed chamber was loaded with 80 ml of a 0.1 M simulated seawater solution, while the opposite chamber was filled with 80 ml of deionized water. To ensure a constant ionic strength throughout the testing, the feed solutions were circulated using a peristaltic pump.

### Concentration gradient electrodialysis and multistage electrodialysis

Given the prevalence of ions such as K^+^, Na^+^, Mg^2+^, Cl^−^, and SO_4_^2−^ in natural water sources, we evaluated the ion separation capabilities of the Q_PIM-DT_10 membrane under binary-salt conditions using NaCl and Na_2_SO_4_, KCl, and MgCl_2_. We investigated the impact of varying salt concentrations on membrane separation performance. The mixed salt concentration gradients are 0.1, 0.5, 1, and 2 M, respectively.

To demonstrate the practical potential of the Q_PIM-DT_10 membrane for direct lithium extraction, we integrated our developed membranes into electrodialysis stacks and performed multistage electrodialysis. The feed was a simulated sulfate-type brine from East Taijiner Salt Lake with an Mg^2+^/Li^+^ ratio of 40, and the composition is shown in table S1. The concentrated compartment solution from the primary electrodialysis was transferred to the desalination compartment of the secondary electrodialysis after concentration. This step was repeated three times. The permeate was concentrated until the Li_2_CO_3_ content reached approximately 5 wt %, after which excess Na_2_CO_3_ solution (39.5 g liter^−1^) was added to induce Li_2_CO_3_ precipitation at 80 °C. Under these conditions, Na^+^, K^+^, and SO_4_^2−^ salts remain substantially more soluble than Li_2_CO_3_ and therefore largely stay in solution. For instance, at 80°C, the solubilities of Li_2_CO_3_, K_2_CO_3_, and Na_2_CO_3_ are 8.5, 139.8, and 45.8 g/100 g H_2_O, respectively, while sulfate salts are generally also highly soluble under these conditions (*53*). The precipitated product was then filtered, washed with deionized water, and dried in a vacuum oven at 50°C for 12 hours.

The Li recovery in multistage electrodialysis can be calculated using the following Eq. 9

$$R_{\mathrm{Li}}=\frac{C_{p}V_{p}}{C_{0}V_{0}}\times 100\%$$ (9)

where *V*_p_ (liter) and *C*_p_ (g liter^−1^) represent the volume and Li^+^ concentration of the final product (5.42 g liter^−1^ and 200 ml), respectively, while *V*_0_ (liter) and C_0_ (g liter^−1^) denote the volume and Li^+^ concentration of the initial brine (0.14 g liter^−1^ and 10 liters).

## Supplementary Materials

### The PDF file includes:

Supplementary Text Figs. S1 to S36 Tables S1 to S4 Legends for datasets S1 to S30 Legend for file S1 References

### Other Supplementary Material for this manuscript includes the following:

Datasets S1 to S30

## References

[1] P. Zuo, C. Ye, Z. Jiao, J. Luo, J. Fang, U. S. Schubert, N. B. McKeown, T. L. Liu, Z. Yang, T. Xu, Near-frictionless ion transport within triazine framework membranes. Nature 617, 299–305 (2023). 10.1038/s41586-023-05888-x PMC10131500 37100908

[2] W. Xu, Y. Wang, Y. Wu, F. Xu, L. Dai, K. Qu, J. Wang, J. Wu, L. Lei, S. Li, Z. Xu, Sub-2-nm channels within covalent triazine framework enable fast proton-selective transport in flow battery membrane. Adv. Funct. Mater. 33, 2300138 (2023).

[3] J.-N. Chang, K. Yang, L.-N. Wang, J. Wang, Y.-Q. Lan, C. Wang, Plasmonic ion diode membrane (PIDM) for enhanced nanofluidic ion transport. Angew. Chem. Int. Ed. 64, e202502591 (2025). 10.1002/anie.202502591 40268680

[4] Y. Huang, C. Liao, Q. Song, Z. Yuan, X. Li, Chemically cross-linked polybenzimidazole membranes with ion-conductive sub-nanometer channels for zinc-iron flow batteries. Angew. Chem. Int. Ed. 64, e202511744 (2025). 10.1002/anie.202511744 40709836

[5] K. Liu, R. Epsztein, S. Lin, L. Liu, J. Qu, M. Sun, Electrostatic-driven dehydration of ions in nanoporous membranes. Sci. Adv. 11, eadv0174 (2025). 10.1126/sciadv.adv0174 PMC12396314 40880460

[6] C. Hu, H. W. Kang, S. W. Jung, M. L. Liu, Y. J. Lee, J. H. Park, N. Y. Kang, M. G. Kim, S. J. Yoo, C. H. Park, Y. M. Lee, High free volume polyelectrolytes for anion exchange membrane water electrolyzers with a current density of 13.39 A cm^−2^ and a durability of 1000 h. Adv. Sci. 11, e2306988 (2024). 10.1002/advs.202306988 PMC10837377 38044283

[7] R. Wang, S. Lin, Membrane design principles for ion-selective electrodialysis: An analysis for Li/Mg separation. Environ. Sci. Technol. 58, 3552–3563 (2024). 10.1021/acs.est.3c08956 PMC10882969 38324772

[8] G. Xu, J. Pan, X. Zou, Z. Jin, J. Zhang, P. Fang, Q. Zhang, Z. Sun, F. Yan, High-performance poly(biphenyl piperidinium) type anion exchange membranes with interconnected ion transfer channels: Cooperativity of dual cations and fluorinated side chains. Adv. Funct. Mater. 33, 2302364 (2023).

[9] D. Liu, Y. Xie, Z. Zhao, J. Li, J. Pang, Z. Jiang, Structural optimization and performance trade-off strategies for semi-crystalline sulfonated poly (arylene ether ketone) membranes in high-concentration direct methanol fuel cells. J. Energy Chem. 85, 67–75 (2023).

[10] M. Atwa, S. Xu, X. Li, S. Dull, Z. Wang, H. Yu, R. Tang, T. Goh, Y. Jung, M. Kiani, M. Lid, H. Nishihara, F. B. Prinz, V. Birss, Controlled distribution of both Pt nanoparticles and ionomer in self-supported nanoporous carbon scaffolds significantly enhances oxygen reduction kinetics and stability. J. Mater. Chem. A 13, 33587–33600 (2025).

[11] A. Wang, C. Breakwell, F. Foglia, R. Tan, L. Lovell, X. Wei, T. Wong, N. Meng, H. Li, A. Seel, M. Sarter, K. Smith, A. Alvarez-Fernandez, M. Furedi, S. Guldin, M. M. Britton, N. B. McKeown, K. E. Jelfs, Q. Song, Selective ion transport through hydrated micropores in polymer membranes. Nature 635, 353–358 (2024). 10.1038/s41586-024-08140-2 PMC11560840 39506120

[12] Y. Ma, C. Hu, G. Yi, Z. Jiang, X. Su, Q. Liu, J. Y. Lee, S. Y. Lee, Y. M. Lee, Q. Zhang, Durable multiblock poly (biphenyl alkylene) anion exchange membranes with microphase separation for hydrogen energy conversion. Angew. Chem. Int. Ed. 62, e202311509 (2023). 10.1002/anie.202311509 37646106

[13] Y. J. Shin, M. Kim, H. Kim, Y. Y. Kim, N. H. Kwon, A. Machida, Y. Nakahira, S. Kim, K. Sakaki, W. B. Lee, E. S. Cho, Surface-engineered asymmetric covalent organic framework membranes with polymers for improved ion transport. ACS Nano 19, 34150–34162 (2025). 10.1021/acsnano.5c10521 40963101

[14] Y. Liu, J. Song, Z. Liu, J. Chen, D. Wang, H. Zhi, J. Tang, Y. Zhang, N. Li, W. Zhou, M. An, H. Liu, G. Xue, Anti-swelling polyelectrolyte hydrogel with submillimeter lateral confinement for osmotic energy conversion. Nano Micro Lett. 17, 81 (2024). 10.1007/s40820-024-01577-0 PMC11612061 39623075

[15] R.-J. Mo, S. Chen, L.-Q. Huang, X.-L. Ding, S. Rafique, X.-H. Xia, Z.-Q. Li, Regulating ion affinity and dehydration of metal-organic framework sub-nanochannels for high-precision ion separation. Nat. Commun. 15, 2145 (2024). 10.1038/s41467-024-46378-6 PMC10924084 38459053

[16] W. Meng, S. Chen, M. Wu, F. Gao, Y. Hou, X. Zhan, W. Hu, L. Liang, Q. Zhang, Dehydration-enhanced ion recognition of triazine covalent organic frameworks for high-resolution Li^+^/Mg^2+^ Separation. Angew. Chem. Int. Ed. 64, e202422423 (2025). 10.1002/anie.202422423 39834313

[17] T. Xu, B. Wu, L. Hou, Y. Zhu, F. Sheng, Z. Zhao, Y. Dong, J. Liu, B. Ye, X. Li, L. Ge, H. Wang, T. Xu, Highly ion-permselective porous organic cage membranes with hierarchical channels. J. Am. Chem. Soc. 144, 10220–10229 (2022). 10.1021/jacs.2c00318 35586909

[18] R. Tan, H. He, A. Wang, T. Wong, Y. Yang, S. Iguodala, C. Ye, D. Liu, Z. Fan, M. Furedi, G. He, S. Guldin, D. J. L. Brett, N. B. McKeown, Q. Song, Interfacial engineering of polymer membranes with intrinsic microporosity for dendrite-free zinc metal batteries. Angew. Chem. Int. Ed. 63, e202409322 (2024). 10.1002/anie.202409322 39195347

[19] Z. Li, H. Li, C. Jin, J. Li, J. Bao, X. Zhang, N. Zhang, G. He, C. Chen, Y. Song, Confined in situ synthesis of uniformly distributed mixed matrix membranes via solvent evaporation for efficient CO_2_ capture. Adv. Funct. Mater. 35, e2420713 (2025).

[20] T. H. Lee, M. Balcik, W.-N. Wu, I. Pinnau, Z. P. Smith, Dual-phase microporous polymer nanofilms by interfacial polymerization for ultrafast molecular separation. Sci. Adv. 10, eadp6666 (2024). 10.1126/sciadv.adp6666 PMC11323956 39141741

[21] S. Guo, C. Fang, J. Li, X. Wang, W. Feng, H. Guo, M. Xie, Y. Zhuang, Y. M. Lee, L. Zhu, Microporous polymer membranes: Molecular stents enhanced solvent-accessibility for organic solvent transport. Adv. Sci. 12, e16748 (2025). 10.1002/advs.202416748 PMC12376686 40439596

[22] R. Tan, A. Wang, R. Malpass-Evans, R. Williams, E. W. Zhao, T. Liu, C. Ye, X. Zhou, B. P. Darwich, Z. Fan, L. Turcani, E. Jackson, L. Chen, S. Y. Chong, T. Li, K. E. Jelfs, A. I. Cooper, N. P. Brandon, C. P. Grey, N. B. McKeown, Q. Song, Hydrophilic microporous membranes for selective ion separation and flow-battery energy storage. Nat. Mater. 19, 195–202 (2020). 10.1038/s41563-019-0536-8 31792424

[23] Y. Wang, N. Alaslai, B. Ghanem, X. Ma, X. Hu, M. Balcik, Q. Liu, M. A. Abdulhamid, Y. Han, M. Eddaoudi, I. Pinnau, Hydroxyl-functionalized polymers of intrinsic microporosity and dual-functionalized blends for high-performance membrane-based gas separations. Adv. Mater. 36, e202406076 (2024). 10.1002/adma.202406076 39324252

[24] M. J. Baran, M. N. Braten, S. Sahu, A. Baskin, S. M. Meckler, L. Li, L. Maserati, M. E. Carrington, Y.-M. Chiang, D. Prendergast, B. A. Helms, Design rules for membranes from polymers of intrinsic microporosity for crossover-free aqueous electrochemical devices. Joule 3, 2968–2985 (2019).

[25] E. Yang, M. Kim, Y. Liang, J. Byun, H. Kim, K. C. Mamani, I. S. Kim, H. Choi, Facile fabrication of robust and tight PIMs-based TFC hollow fiber membranes with a semi-interpenetrating polymer network via liquid phase cross-linking for organic solvent nanofiltration. J. Membr. Sci. 713, 123338 (2025).

[26] L. Sun, W. Xu, H. Zhang, J. Chu, M. Wang, K. Song, W. Wu, J. Li, Y. Wang, I. Pinnau, X. Ma, In-situ formation of three-dimensional network intrinsic microporous ladder polymer membranes with ultra-high gas separation performance and anti-trade-off effect. Angew. Chem. Int. Ed. 64, e202420742 (2025). 10.1002/anie.202420742 39822022

[27] X. Yang, Z. Ullah, J. F. Stoddart, C. T. Yavuz, Porous organic cages. Chem. Rev. 123, 4602–4634 (2023). 10.1021/acs.chemrev.2c00667 PMC10141292 37023354

[28] G.-H. He, L. Li, H. Xu, Y. Ji, P. Wang, A polymer membrane with integrated microphase separation and intrinsic microporosity for aqueous organic redox flow batteries. Joule 9, 101976 (2025).

[29] Z. Zhang, L. Wen, L. Jiang, Nanofluidics for osmotic energy conversion. Nat. Rev. Mater. 6, 622–639 (2021).

[30] R. Wang, R. He, T. He, M. Elimelech, S. Lin, Performance metrics for nanofiltration-based selective separation for resource extraction and recovery. Nat. Water 1, 291–300 (2023).

[31] J. S. Chen, J. Wang, J. H. Zhang, Z. Y. Guo, P. P. Zhang, X. F. Guo, J. Liu, Z. Y. Ji, Electronanofiltration membranes with a bilayer charged structure enable high Li^+^/Mg^2+^ selectivity. ACS Appl. Mater. Interfaces 16, 6632–6643 (2024). 10.1021/acsami.3c16092 38272023

[32] R. Wang, M. Sheng, X. T. Liu, Q. Y. Wang, Z. Wang, L. Wang, Electrosorption theoretically outperforms nanofiltration and electrodialysis for direct lithium extraction from brines. Environ. Sci. Technol. 60, 1577–1595 (2026). 10.1021/acs.est.5c13571 41401054

[33] Y. Wu, C. Lu, C. Hu, J. Qu, Combined effect of electrostatic interaction and steric sieving during ion transport in nanochannels. Environ. Sci. Technol. 59, 11377–11388 (2025). 10.1021/acs.est.5c02112 40445042

[34] M. Wang, P. Zhang, X. Liang, J. Zhao, Y. Liu, Y. Cao, H. Wang, Y. Chen, Z. Zhang, F. Pan, Z. Zhang, Z. Jiang, Ultrafast seawater desalination with covalent organic framework membranes. Nat. Sustain. 5, 518–526 (2022).

[35] H. Sun, N. Gan, Y. Lin, B. Wu, Y. Qiu, J. Su, Z. Zhou, F. Zou, J. Yu, H. Matsuyama, Ultramicroporous Tröger’s base framework membranes with ionized sub-nanochannels for efficient acid/alkali recovery. Adv. Sci. 12, e2414280 (2025). 10.1002/advs.202414280 PMC11884555 39807014

[36] B. Zhang, H. Zhang, Y. Wang, S. Fang, Adsorption behavior and mechanism of amine/quaternary ammonium lignin on tungsten. Int. J. Biol. Macromol. 216, 882–890 (2022). 10.1016/j.ijbiomac.2022.07.226 35926372

[37] D. Elizebath, B. Vedhanarayanan, A. Dhiman, R. K. Mishra, C. N. Ramachandran, T.-W. Lin, V. K. Praveen, Spontaneous curvature induction in an artificial bilayer membrane. Angew. Chem. Int. Ed. 63, e202403900 (2024). 10.1002/anie.202403900 38459961

[38] B. A. Corzo, H. Hernández-Martínez, E. J. Aldeco-Pérez, J. Cárdenas, V. Lara, L. I. Olvera, Functionalized imidazolium ether-free polymer backbones with ion transport channels and catalytic activity. ACS Mater. Au 5, 508–521 (2025). 10.1021/acsmaterialsau.4c00154 PMC12082359 40385953

[39] H. Amiri, K. L. Shepard, C. Nuckolls, R. Hernández Sánchez, Single-walled carbon nanotubes: Mimics of biological ion channels. Nano Lett. 17, 1204–1211 (2017). 10.1021/acs.nanolett.6b04967 PMC5301282 28103039

[40] A. Wang, R. Tan, D. Z. Liu, J. Lu, X. Wei, A. Alvarez-Fernandez, C. Ye, C. Breakwell, S. Guldin, A. R. Kucernak, K. E. Jelfs, N. P. Brandon, N. B. McKeown, Q. Song, Ion-selective microporous polymer membranes with hydrogen-bond and salt-bridge networks for aqueous organic redox flow batteries. Adv. Mater. 35, e2210098 (2023). 10.1002/adma.202210098 36634684

[41] G. Suresh, Y. Scindia, A. Pandey, A. Goswami, Self-diffusion coefficient of water in Nafion-117 membrane with different monovalent counterions: A radiotracer study. J. Membr. Sci. 250, 39–45 (2005).

[42] L. M. Thieu, L. Zhu, A. G. Korovich, M. A. Hickner, L. A. Madsen, Multiscale tortuous diffusion in anion and cation exchange membranes. Macromolecules 52, 24–35 (2018).

[43] C. Liu, X. Zou, Y. Lv, X. Liu, C. Ma, K. Li, Y. Liu, Y. Chai, L. Liao, J. He, Controllable van der Waals gaps by water adsorption. Nat. Nanotechnol. 19, 448–454 (2024). 10.1038/s41565-023-01579-w 38177277

[44] L. Jiang, L. Chen, C. Cao, K. Chen, C. Ji, Y. Zhang, F. Gan, Coupling CO_2_ capture process with electrochemically enhanced membrane distillation system for lithium-ion battery recovery: Reagent-Saving and environmental footprint reducing. Water Res. 272, 122976 (2025). 10.1016/j.watres.2024.122976 39693792

[45] H. Cho, J. Kim, C.-S. Han, Multi-ion transport in reverse electrodialysis: A validated model for design and optimization. Desalination 586, 117746 (2024).

[46] J. W. Colson, A. R. Woll, A. Mukherjee, M. P. Levendorf, E. L. Spitler, V. B. Shields, M. G. Spencer, J. Park, W. R. Dichtel, Oriented 2D covalent organic framework thin films on single-layer graphene. Science 332, 228–231 (2011). 10.1126/science.1202747 21474758

[47] D. Yang, Y. Yang, T. Wong, S. Iguodala, A. Wang, L. Lovell, F. Foglia, P. Fouquet, C. Breakwell, Z. Fan, Y. Wang, M. M. Britton, D. R. Williams, N. Shah, T. Xu, N. B. McKeown, M.-M. Titirici, K. E. Jelfs, Q. Song, Solution-processable polymer membranes with hydrophilic subnanometre pores for sustainable lithium extraction. Nat. Water 3, 319–333 (2025). 10.1038/s44221-025-00398-8 PMC11932922 40144313

[48] Q.-W. Meng, J. Li, Z. Xing, W. Xian, Z. Lai, Z. Dai, S. Wang, L. Zhang, H. Yin, S. Ma, Q. Sun, Regulation of ion binding sites in covalent organic framework membranes for enhanced selectivity under high ionic competition. ACS Nano 19, 12080–12089 (2025). 10.1021/acsnano.4c18135 40101969

[49] Y. Li, R. Jia, R. Tan, Z. Hong, J. Gu, P. Cui, S. Zhang, H. Shao, J. Ran, C.-F. Fu, Ionization-engineered two-dimensional confined channels in GO membranes for highly efficient OH^−^ separation. AIChE J. 70, e18298 (2023).

[50] J. Yan, H. Wang, R. Fu, R. Fu, R. Li, B. Chen, C. Jiang, L. Ge, Z. Liu, Y. Wang, T. Xu, Ion exchange membranes for acid recovery: Diffusion dialysis (DD) or selective electrodialysis (SED). Desalination 531, 115690 (2022).

[51] W. Ji, N. U. Afsar, B. Wu, F. Sheng, M. A. Shehzad, L. Ge, T. Xu, In-situ crosslinked SPPO/PVA composite membranes for alkali recovery via diffusion dialysis. J. Membr. Sci. 590, 117267 (2019).

[52] R. Mondal, S. Pal, U. Chatterjee, Alkylated imidazole moieties in a cross-linked anion exchange membrane facilitate acid recovery with high purity. ACS Appl. Polym. Mater. 3, 1544–1554 (2021).

[53] M. Shi, L. A. Diaz, J. R. Klaehn, A. D. Wilson, T. E. Lister, Li_2_CO_3_ Recovery through a carbon-negative electrodialysis of lithium-ion battery leachates. ACS Sustain. Chem. Eng. 10, 11773–11781 (2022).

[54] F. Soyekwo, C. Liu, H. Wen, S. Raza, Polyamide–based membranes with nanoscale homogeneity and asymmetric structure for ultrafast ion separation. J. Membr. Sci. 685, 121921 (2023).

[55] Y. Liang, Y. Zhu, C. Liu, K.-R. Lee, W.-S. Hung, Z. Wang, Y. Li, M. Elimelech, J. Jin, S. Lin, Polyamide nanofiltration membrane with highly uniform sub-nanometre pores for sub-1 Å precision separation. Nat. Commun. 11, 2015 (2020). 10.1038/s41467-020-15771-2 PMC7181833 32332724

[56] G. Gong, P. Wang, Z. Zhou, Y. Hu, New insights into the role of an interlayer for the fabrication of highly selective and permeable thin-film composite nanofiltration membrane. ACS Appl. Mater. Interfaces 11, 7349–7356 (2019). 10.1021/acsami.8b18719 30668908

[57] C. Ji, C.-W. Lin, S. Zhang, Y. Guo, Z. Yang, W. Hu, S. Xue, Q. J. Niu, R. B. Kaner, Ultrapermeable nanofiltration membranes with tunable selectivity fabricated with polyaniline nanofibers. J. Mater. Chem. A 10, 4392–4401 (2022).

[58] J. Zhao, X. You, G. Wang, J. Yuan, Y. Li, C. Yang, S. Zhang, X. Wang, R. Zhang, H. Wu, Z. Jiang, Mix-charged polyamide membranes via molecular hybridization for selective ionic nanofiltration. J. Membr. Sci. 644, 120051 (2022).

[59] Z. L. Qiu, W. H. Yu, Y. J. Shen, B. K. Zhu, L. F. Fang, Janus charged polyamide nanofilm with ultra-high separation selectivity for mono−/divalent ions. Chem. Eng. J. 416, 129023 (2021).

[60] Y. Zhang, H. Wang, J. Guo, X. Cheng, G. Han, C. H. Lau, H. Lin, S. M. Liu, J. Ma, L. Shao, Ice-confined synthesis of highly ionized 3D-quasilayered polyamide nanofiltration membranes. Science 382, 202–206 (2023). 10.1126/science.adi9531 37824644

[61] B. Yuan, S. Zhao, P. Hu, J. Cui, Q. J. Niu, Asymmetric polyamide nanofilms with highly ordered nanovoids for water purification. Nat. Commun. 11, 6102 (2020). 10.1038/s41467-020-19809-3 PMC7705655 33257695

[62] S. Han, J. Zhu, A. A. Uliana, D. Li, Y. Zhang, L. Zhang, Y. Wang, T. He, M. Elimelech, Microporous organic nanotube assisted design of high performance nanofiltration membranes. Nat. Commun. 13, 7954 (2022). 10.1038/s41467-022-35681-9 PMC9794819 36575167

[63] Y. Zhang, R. Dong, U. R. Gabinet, R. Poling-Skutvik, N. K. Kim, C. Lee, O. Q. Imran, X. Feng, C. O. Osuji, Rapid fabrication by lyotropic self-assembly of thin nanofiltration membranes with uniform 1 nanometer pores. ACS Nano 15, 8192–8203 (2021). 10.1021/acsnano.1c00722 33729764

[64] Z.-L. Qiu, L.-F. Fang, Y.-J. Shen, W.-H. Yu, B.-K. Zhu, C. Hélix-Nielsen, W. Zhang, Ionic dendrimer based polyamide membranes for ion separation. ACS Nano 15, 7522–7535 (2021). 10.1021/acsnano.1c00936 33779134

[65] G. Lu, S. Lu, J. Sun, M. W. Boey, W. Shang, J. Wu, A. K. An, Nano-confined supramolecular assembly of ultrathin crystalline polymer membranes for high-performance nanofiltration. Adv. Funct. Mater. 34, 2309913 (2024).

[66] Y. Chen, W. Wang, X. Liu, X. Duan, Y. Ren, Nanocapsules induce in situ grafting of polyelectrolytes and structural rearrangement of polyamide layers. Chem. Eng. Sci. 308, 121437 (2025).

[67] Q. Zhou, M. Chen, J. Lu, B. Sheng, J. Chen, Q. Zhang, X. Han, Wide-temperature solid polymer electrolytes: Li^+^ coordination structure, ionic transport and interphases. Mater. Horiz. 12, 3201–3233 (2025). 10.1039/d4mh01869a 39989217

[68] Y. Lin, X. Yao, Q. Shen, T. Ueda, Y. Kawabata, J. Segawa, K. Guan, T. Istirokhatun, Q. Song, T. Yoshioka, H. Matsuyama, Zwitterionic copolymer-regulated interfacial polymerization for highly permselective nanofiltration membrane. Nano Lett. 21, 6525–6532 (2021). 10.1021/acs.nanolett.1c01711 34339209

[69] W. L. Li, W. T. Lin, C. Y. Zhu, P. Fu, D. Zhou, X. J. Huang, Z. K. Xu, L. S. Wan, High-performance thin film composite nanofiltration membranes with MoS_2_ nanosheet interlayer. J. Membr. Sci. 685, 121956 (2023).

[70] Z. Liu, Z. Ma, B. Qian, A. Y. H. Chan, X. Wang, Y. Liu, J. H. Xin, A facile and scalable method of fabrication of large-area ultrathin graphene oxide nanofiltration membrane. ACS Nano 15, 15294–15305 (2021). 10.1021/acsnano.1c06155 34478273

[71] H. Qian, P. Peng, H. Fan, Z. Yang, L. Yang, Y. Zhou, D. Tan, F. Yang, M. Willatzen, G. Amaratunga, Z. Wang, D. Wei, Horizontal transport in Ti_3_C_2_T_x_ MXene for highly efficient osmotic energy conversion from saline-alkali environments. Angew. Chem. Int. Ed. 63, e202414984 (2024). 10.1002/anie.202414984 39147723

[72] T.-H. Chen, T. Su, L. Xia, L.-Y. Gong, B.-N. Dong, L.-F. Liu, Corolliform morphology thin film composite polyamide membrane constructed via Tröger’s base PIM for enhancing nanofiltration separation performances. Desalination 548, 116234 (2023).

[73] X.-F. Hu, T. Jiang, H. Fan, Y.-F. Guan, J.-J. Chen, H.-Q. Yu, M. Elimelech, Dual-regulated covalent organic framework membranes with near-theoretical pore sizes for angstrom-scale ion separations. Sci. Adv. 11, eady3587 (2025). 10.1126/sciadv.ady3587 PMC12609051 41223271

[74] F. Yang, J. Guo, C. Han, J. Huang, Z. Zhou, S.-P. Sun, Y. Zhang, L. Shao, Turing covalent organic framework membranes via heterogeneous nucleation synthesis for organic solvent nanofiltration. Sci. Adv. 10, eadr9260 (2024). 10.1126/sciadv.adr9260 PMC11633759 39661688

[75] J. Shen, J. Yuan, B. Shi, X. You, R. Ding, T. Zhang, Y. Zhang, Y. Deng, J. Guan, M. Long, Y. Zheng, R. Zhang, H. Wu, Z. Jiang, Homointerface covalent organic framework membranes for efficient desalination. J. Mater. Chem. A 9, 23178–23187 (2021).

[76] M. Wang, Z.-H. Wu, G.-Z. Zhou, X.-Q. Wu, S. Li, T.-Y. Ma, Y. Ren, G. Hai, B. Wang, D.-S. Li, Partitioning COF membrane channels into ultramicroporous and π-electron-rich compartments for multicomponent ion separations. Angew. Chem. Int. Ed. 64, e202514179 (2025). 10.1002/anie.202514179 40944379

[77] Y.-X. Fang, Y.-F. Lin, Z.-L. Xu, D. Pandaya, S.-J. Xu, Interfacial polymerization modulated by COFs interlayer to fabricate efficient mono/divalent ions separation nanofiltration membrane. Desalination 588, 117977 (2024).

[78] Y.-L. Ji, B.-X. Gu, S.-J. Xie, M.-J. Yin, W.-J. Qian, Q. Zhao, W.-S. Hung, K.-R. Lee, Y. Zhou, Q.-F. An, C.-J. Gao, Superfast water transport zwitterionic polymeric nanofluidic membrane reinforced by metal-organic frameworks. Adv. Mater. 33, e2102292 (2021). 10.1002/adma.202102292 34346108

[79] Y. Chen, T. Zhang, D. Chai, H. Ye, S. Tang, P. Wang, H. Sun, B. Ge, Q. J. Niu, Enhancing the NaCl/Na_2_SO_4_ separation selectivity and chlorine resistance of nanofiltration membranes by incorporating novel designed starch nanoparticles. Appl. Surf. Sci. 604, 154417 (2022).

[80] M. Jian, R. Qiu, Y. Xia, J. Lu, Y. Chen, Q. Gu, R. Liu, C. Hu, J. Qu, H. Wang, X. Zhang, Ultrathin water-stable metal-organic framework membranes for ion separation. Sci. Adv. 6, eaay3998 (2020). 10.1126/sciadv.aay3998 PMC7274808 32548253

[81] P. Ramirez, J. Cervera, S. Nasir, M. Ali, W. Ensinger, S. Mafe, Electrochemical impedance spectroscopy of membranes with nanofluidic conical pores. J. Colloid Interface Sci. 655, 876–885 (2024). 10.1016/j.jcis.2023.11.060 37979293

[82] S. Wang, Y. Huang, Y. Qiang, M. Wu, S. Liang, J. Wang, C. Fang, L. Zhu, Nanoconfined electrostatic interaction for efficient anion sieving in graphene oxide membranes. Water Res. 270, 122855 (2025). 10.1016/j.watres.2024.122855 39612816

[83] D. Wu, Z. Xing, Q. Guo, Z. Lai, J. Yi, Q.-W. Meng, S. Wang, Z. Dai, S. Ma, Q. Sun, Engineering bipolar covalent organic framework membranes for selective acid extraction. Angew. Chem. Int. Ed. 64, e202503945 (2025). 10.1002/anie.202503945 40295221

[84] B. Wu, Y. Lin, N. Gan, Y. Qiu, H. Sun, J. Su, P. Huang, X. Lin, J. Yu, T. Yoshioka, H. Matsuyama, Bioinspired membrane with Angstrom-scale channels: A versatile nanofluidic platform for efficient single-ion sieving. AIChE J. 72, e70098 (2026).

[85] H. Zhang, W. Xu, W. Song, K. Peng, L. Sun, C. Yang, X. Zhang, H. Zhang, B. Ye, X. Liang, Z. Yang, L. Wu, X. Ge, T. Xu, High-performance spiro-branched polymeric membranes for sustainability applications. Nat. Sustain. 7, 910–919 (2024).

[86] S.-Y. Sun, L.-J. Cai, X.-Y. Nie, X. Song, J.-G. Yu, Separation of magnesium and lithium from brine using a Desal nanofiltration membrane. J. Water Process Eng. 7, 210–217 (2015).

[87] H. Kazemzadeh, J. Karimi-Sabet, J. T. Darian, A. Adhami, Evaluation of polymer inclusion membrane efficiency in selective separation of lithium ion from aqueous solution. Sep. Purif. Technol. 251, 117298 (2020).

[88] C. Zhang, Y. Mu, S. Zhao, W. Zhang, Y. Wang, Lithium extraction from synthetic brine with high Mg^2+^/Li^+^ ratio using the polymer inclusion membrane. Desalination 496, 114710 (2020).

[89] L. Xu, X. Zeng, Q. He, T. Deng, C. Zhang, W. Zhang, Stable ionic liquid-based polymer inclusion membranes for lithium and magnesium separation. Sep. Purif. Technol. 288, 120626 (2022).

[90] W. Shen, D. Wang, Y. Tian, F. Zhou, Y. Lin, Z. Zhang, J. Xu, C. Yang, Highly permeable and selective polymer inclusion membrane for Li^+^ recovery and underlying enhanced mechanism. J. Membr. Sci. 699, 122671 (2024).

[91] J. Ying, Z. Cui, L. Tu, T. Liu, S. Lu, Q. Shen, M. Guo, Y. Wang, Z. Yang, M. Zeng, Z. Yu, X. Cao, Y. Lin, Polymers with intrinsic microporosity engineered via acid-base pairs for highly selective lithium-ion transport channels. J. Membr. Sci. 741, 125059 (2026).

[92] C. Bandl, W. Kern, M. Ranz, B. Grabner, Functionalization of PTFE substrates with quaternary ammonium groups—An approach towards anion conducting properties. Appl. Surf. Sci. 661, 160084 (2024).

[93] W. Wang, G. Hong, Y. Zhang, X. Yang, N. Hu, J. Zhang, P. Sorokin, L. Shao, Designing an energy-efficient multi-stage selective electrodialysis process based on high-performance materials for lithium extraction. J. Membr. Sci. 675, 121534 (2023).

[94] W. Meng, S. Chen, Z. Guo, F. Gao, J. Wang, J. Lu, Y. Hou, Q. He, X. Zhan, M. Qiu, Q. Zhang, Three-dimensional cationic covalent organic framework membranes for rapid and selective lithium extraction from saline water. Nat. Water 3, 191–200 (2025).

[95] F. Sheng, B. Wu, X. Li, T. Xu, M. A. Shehzad, X. Wang, L. Ge, H. Wang, T. Xu, Efficient ion sieving in covalent organic framework membranes with sub-2-nanometer channels. Adv. Mater. 33, e2104404 (2021). 10.1002/adma.202104404 34480387

[96] X.-Y. Xie, H.-Q. Huo, W.-H. Zhang, Y. Zhao, Y.-L. Ji, B. Van der Bruggen, C.-J. Gao, Facile surface modification of cation exchange membranes via Debus-Radziszewski in-situ cross-linking for lithium extraction from salt-lakes. Chem. Eng. J. 487, 150617 (2024).

[97] D. Seo, J. Lee, S. R. Kong, G. Sim, Y. Park, Selective lithium extraction from salt-lake brine using LATP-incorporated cellulose membranes in electrically driven systems. J. Membr. Sci. 718, 123676 (2025).

