# Supplementary material for: Hydration channel engineering in Tröger’s base-POP membranes for mono/multivalent cation and anion discrimination
Source: Sci Adv. 2026 Oct 9;12(41):eaef9368. doi: 10.1126/sciadv.aef9368 (PMC13651864; doi:10.1126/sciadv.aef9368)
Supplement: Supplementary file 1 [file sciadv.aef9368_sm.pdf]

Science Advances
AAAS

Supplementary Materials for

Hydration channel engineering in Tröger's base-POP membranes for mono/multivalent cation and anion discrimination

Haopan Sun *et al.*

Corresponding author: Yuqing Lin, linyuqing@people.kobe-u.ac.jp; Hideto Matsuyama, matuyama@kobe-u.ac.jp; Young Moo Lee, ymlee@hanyang.ac.kr

Sci. Adv. **12**, eaef9368 (2026)
DOI: 10.1126/sciadv.aef9368

The PDF file includes:

Supplementary Text
Figs. S1 to S36
Tables S1 to S4
Legends for datasets S1 to S30
Legend for file S1
References

Other Supplementary Material for this manuscript includes the following:

Datasets S1 to S30
File S1

Supplementary Text

1. Supplementary membrane property measurements

Water uptake and swelling ratio

The water uptake and area swelling ratio were determined by measuring the changes in membrane weight and surface area before and after membrane wetting. The square-shaped membrane (1 × 2 cm^2) was initially subjected to thermal treatment at 80 °C for 24 hours. Subsequently, the dry weight (W_{dry}) and dry area (A_{dry}) were measured. The membrane was then immersed in water for an additional 48 hours. After removing the excess surface water, the wet weight (W_{wet}) and wet area (A_{wet}) of the membrane were measured. Three sets of each test were performed, and the water uptake and area swelling ratio (%) were computed employing Equations S1 and S2 (*26*):

$$\text{Water uptake } (\%) = \frac{(W_{wet} - W_{dry})}{W_{dry}} \times 100\% \quad \text{(S1)}$$

$$\text{Area swelling ratio } (\%) = \frac{(A_{wet} - A_{dry})}{A_{dry}} \times 100\% \quad \text{(S2)}$$

Ion exchange capacity

The dried Q_PIM-DT_X membrane was circulated in a 0.5 M Na_2SO_4 salt solution under an electric field for 24 hours. Subsequently, any residual salt solution on the membrane surface was rinsed several times with pure water, wiped dry, and immersed in a 0.5 M NaCl salt solution for 24 hours. The ion exchange capacity (IEC) of the membrane was calculated using the following Equation S3 (*26*):

$$IEC = \frac{(C_{1Cl^-} - C_{2Cl^-})V}{M_{dry}} \quad \text{(S3)}$$

where C_{1Cl^-} and C_{2Cl^-} are the concentrations of Cl^- before and after immersing the membrane, V (30 mL) represents the volume of NaCl solution, and M_{dry} is the quantity of the membrane sample after initial drying.

Electrochemical impedance spectroscopy (EIS)

Electrochemical Impedance Spectroscopy (EIS) was used to measure the ohmic resistance of all membranes, using the FRA impedance potentiostatic mode with a 10 mV bias and a frequency range of 100 kHz to 0.01 Hz. The membranes were saturated in solutions of 0.1 M KCl, 0.1 M $MgCl_2$, 0.1 M NaCl, or 0.1 M Na_2SO_4 and subsequently installed in a self-made four-electrode device. The ohmic resistance (Rohmic) of the membranes was determined from the intersection points of the EIS curves with the x-axis. The ionic conductivity of these membranes, over a temperature range from room temperature to 60°C, can be calculated using the following Equation S4 (*81*):

$$\sigma = \frac{l}{A \times R_{ohmic}} \quad \text{(S4)}$$

where σ is the ionic conductivity, l is the membrane thickness, A is the effective area, and R_{ohmic} is the membrane resistance. The effective membrane area is 1.7 cm^2. The overall thicknesses of the membranes were determined by a micrometer, and the thickness of the selective layer was tested by SEM.

Zeta potential

The surface charge properties of the Q_PIM-DT_X membranes were investigated using an electrokinetic analyzer (SurPass 3, Anton Paar, Australia). A 1 mM KCl aqueous solution served

as the background electrolyte, and measurements were conducted over a pH range of 3-10 at 25°C. The zeta potential (ζ) was calculated using the Helmholtz-Smoluchowski Equation S5 (*82*):

$$\zeta = \frac{dU}{dP} \times \frac{\eta}{\varepsilon\varepsilon_0} \times \kappa \tag{S5}$$

where U and P denote the streaming potential and pressure, respectively; η, ε, and κ are the electrolyte viscosity, permittivity, and conductivity, respectively; and ε_0 is the vacuum permittivity.

Ion selectivity calculated based on the ion transmembrane conductance

The selective transport of ions of varying sizes across the Q_PIM-DT_X membrane was evaluated using current-voltage (I-V) measurements in a two-compartment H-cell. The selectivity ratio (SR) of K^+ relative to metal ions was determined by measuring transmembrane ion conductance. Assuming the I–V curves follow Ohm's law, we computed the ratio of the respective currents at +1 V using the following Equation S6, taking into account valence differences:

$$SR_{K^+/M^*} = \frac{I_{KCl}}{I_{MCl}} \times \frac{Z_{M^*}}{Z_{K^+}} \tag{S6}$$

where I_{KCl} and I_{MCl} are the currents at +1 V of KCl and metal chloride electrolytes, respectively, at the same concentration; Z_{K+} and Z_{M*} denote the valences of K^+ and metal ions, respectively.

Similarly, for the SR of Cl^- compared to other anions, it was calculated as follows (*83*):

$$SR_{Cl^-/Y^*} = \frac{I_{NaCl}}{I_{NaY}} \times \frac{Z_{Y^*}}{Z_{Cl^-}} \tag{S7}$$

where I_{NaCl} and I_{NaY} are the currents at +1 V of NaCl and other sodium electrolytes, respectively, measured at the same concentration; Z_{Cl-} and Z_{Y*} indicate the valences of Cl^- and other anions, respectively.

2. Molecular Simulation Methods

Model Construction

All molecular models were constructed using BIOVIA Materials Studio 2020. Monomeric and oligomeric building blocks of DMBP, TPB, and their copolymers were first generated. For each polymer type, representative repeat units consisting of 2-3 monomeric units were employed to capture the local chain rigidity and conformational features while maintaining computational tractability. Four categories of polymer models were considered: 1) pristine DMBP, 2) pristine TPB, 3) DMBP-TPB copolymer, and 4) quaternized DMBP-TPB copolymer (Q_PIM-DT, TPB: DMBP molar ratio = 1:10). Geometry optimizations of isolated monomers and oligomers were performed using the DMol3 module with the meta-GGA M06-L functional and an all-electron basis set. Amorphous polymer cells were subsequently generated under the COMPASS III force field. Cubic simulation boxes of 40×40×40 $Å^3$ were constructed with an initial density of 0.8 $g \cdot cm^{-3}$, yielding four independent amorphous cells corresponding to the polymer categories listed above.

Equilibrium molecular dynamics of membrane models

To remove unfavorable chain packing and achieve realistic amorphous morphologies, each system underwent the annealing procedure. Specifically, the systems were gradually heated from 300 K to 800 K followed by cooling back to 300 K under the NPT ensemble for 5 cycles, with a total annealing duration of 5 ns. After annealing, the systems were equilibrated sequentially with an NPT ensemble (1 ns, 300 K and 1 atm, Berendsen thermostat and barostat) to allow full relaxation of density and cell volume; then an NVT ensemble (1 ns, 300 K, Nosé-Hoover thermostat) to stabilize local chain conformations and remove residual pressure fluctuations. The equilibrated dry polymer models were then used for pore-size analysis and void connectivity visualization.

The subsequent hydration models were generated by inserting explicit water molecules into the equilibrated dry cells, yielding an overall initial density of approximately 1.0 $g \cdot cm^{-3}$. Hydrated systems were equilibrated using the same protocol described above. The resulting configurations were analyzed to visualize water clustering, percolation, and connectivity within the polymer matrix, allowing direct identification of continuous internal hydration channels.

Three representative electrolyte systems (LiCl, Li_2SO_4, and $MgCl_2$) were constructed for Q_PIM-DT membrane. In all cases, the polymer-to-water ratio was fixed at 2:10, while the cation/anion ratio was adjusted according to salt valence (LiCl: 2:2; Li_2SO_4: 2:1; $MgCl_2$: 1:2). Ion diffusion coefficients within hydrated polymer channels were obtained from mean square displacement (MSD) analysis (*84*):

$$\mathrm{MSD}(t) = \left\langle |r(t) - r(0)|^2 \right\rangle \tag{S8}$$

The diffusion coefficient D was calculated using the Einstein relation:

$$D = \frac{1}{6} \times \frac{d}{dt} \times \mathrm{MSD}(t) \tag{S9}$$

MSD calculations were performed under NVT conditions, and diffusion coefficients were compared among different ion species to assess mobility.

Non-Equilibrium calculations for ion transport

To quantify ion-transport energetics, potential of mean force (PMF) calculations were performed for Li^+, Mg^{2+}, Cl^-, and SO_4^{2-} across the Q_PIM-DT network. A representative fragment was selected from the pre-equilibrated hydrated membrane model, with a total atom count of 1041, to construct the simulation system in GROMACS 2025.4. The polymer fragment was then solvated in SPCE water. In addition to the target ion under investigation, the corresponding number of counterions was introduced to maintain overall charge neutrality of the simulation box. The OPLS-AA force field was employed for polymer-ion interactions, while ion LJ parameters were generated

using LigParGen. Long-range electrostatics were treated using the Particle Mesh Ewald (PME) method, with a short-range Coulomb and LJ cutoff of 1.0 nm. Temperature was controlled by the V-rescale thermostat, and pressure was controlled by the Berendsen barostat. Water molecules were modeled using the SPCE water model. Umbrella sampling was conducted along the membrane-normal (z) direction. The ion was restrained using a harmonic biasing potential with a force constant of 1000 $kJ\cdot mol^{-1}\cdot nm^{-2}$. Sampling windows were generated at 0.05 nm spacing along the membrane-normal direction, covering the entire translocation pathway. For each window, the system was first equilibrated for 0.2 ns in the NPT ensemble under the harmonic restraint to relax local hydration and polymer configurations, followed by 5.0 ns production run for data collection (typically in NVT). Free-energy profiles were reconstructed using the Weighted Histogram Analysis Method (WHAM).

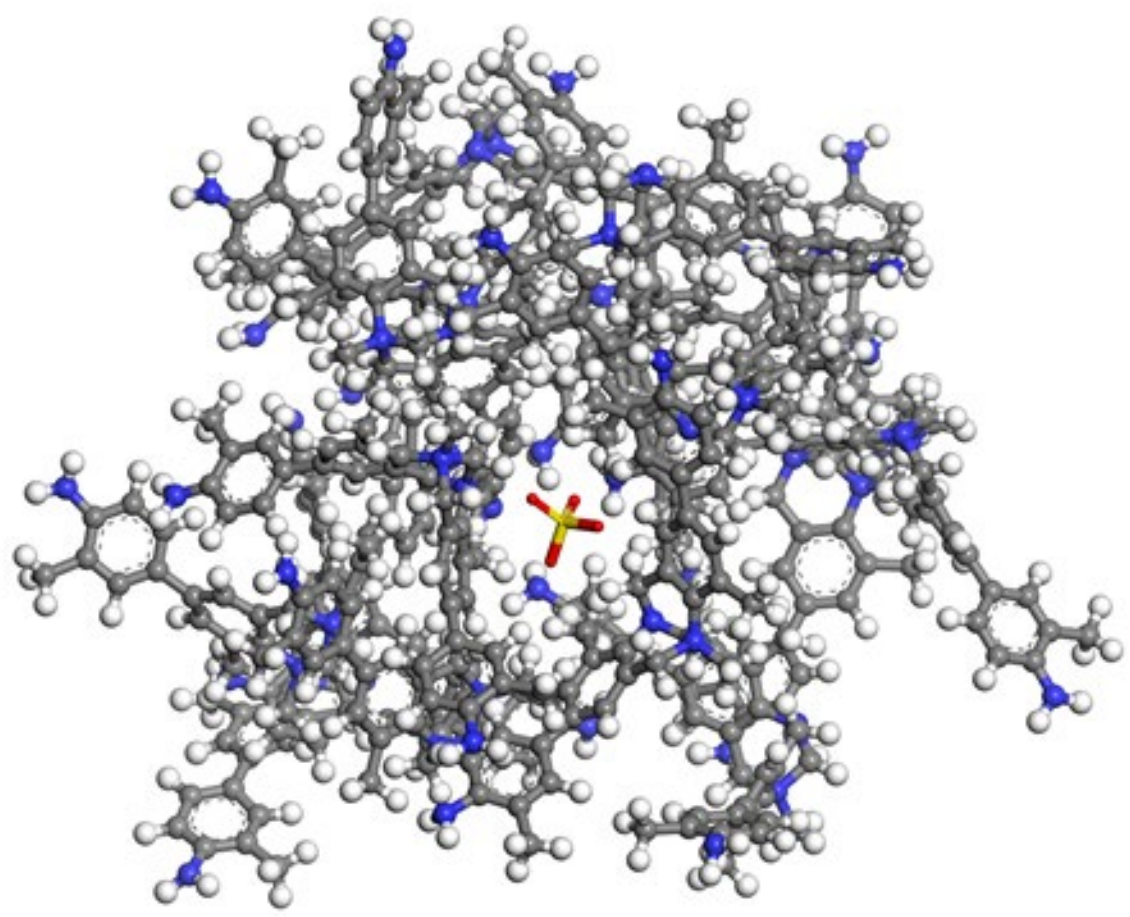

Fig. S1. Representative snapshot of the membrane model used for non-equilibrium ion-transport calculations, with SO_4^{2-} at the pore entrance during translocation.

Quantum-Chemical Analyses along the PMF Pathway

Snapshots corresponding to ions located at the pore entrance were extracted from NE-MD trajectories. To preserve the local chemical environment while keeping the model computationally tractable, only the nearest framework segments around the ion were retained for quantum-chemical calculations. Ion-polymer binding energies were computed using DFT as (*84*):

$$\Delta E_{bind} = E_{complex} - E_{ploymer} - E_{ion} \quad \text{(S10)}$$

All energies were calculated using the DMol3 module with the meta-GGA M06-L functional, an all-electron basis set and the COSMO implicit solvent model, ensuring consistency with earlier geometry optimizations.

Radial distribution functions (RDFs) between ions and surrounding water oxygen atoms were calculated as (*85*):

$$g(r) = \frac{1}{4\pi r^2 \rho}\left\langle \sum_{i,j} \delta(r - r_{ij}) \right\rangle \quad \text{(S11)}$$

where ρ is the number density of water molecules and r_{ij} is the distance between ion i and water oxygen j.

The coordination number (CN) was obtained by integrating the RDF:

$$CN = 4\pi\rho \int_0^r g(r) r^2 dr \quad \text{(S12)}$$

Changes in CN along the PMF coordinate were used to quantify ion dehydration and rehydration during pore entry and translocation.

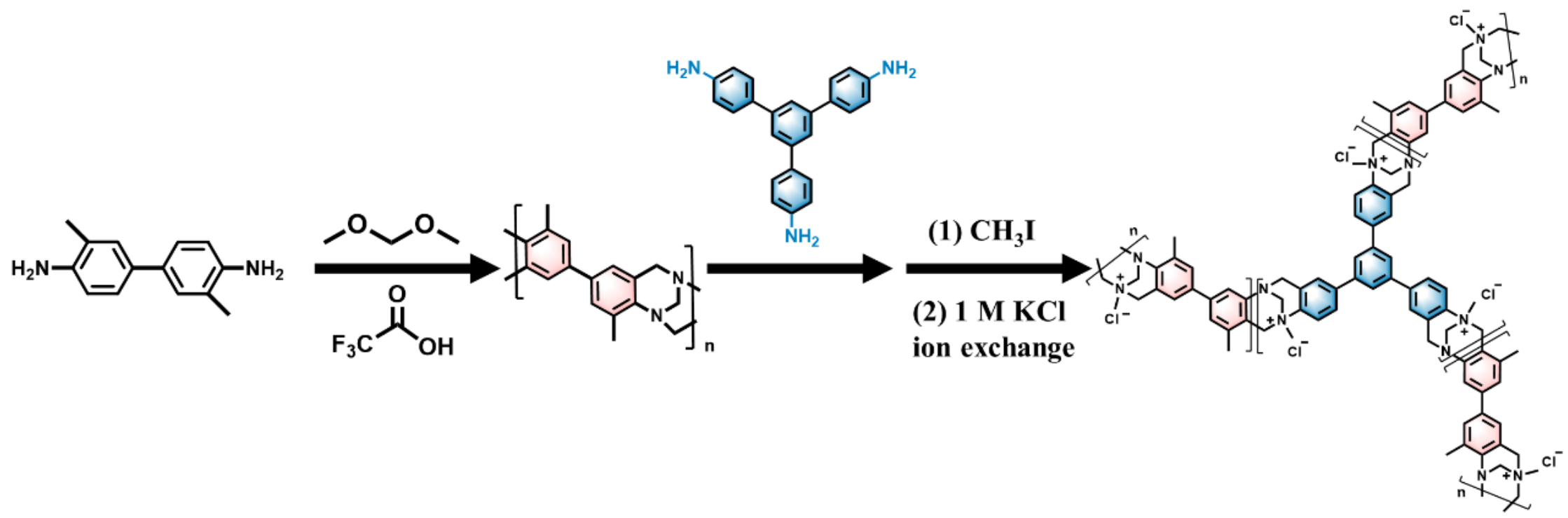

Fig. S2. Synthetic pathway of Q_PIM-DT polymers. Quaternized polymer containing Tröger's base, exhibiting microporosity, can be synthesized via a sequential-synthesis strategy. By varying the DMBP/TPB monomer ratios, a series of network PIM films was synthesized and denoted as PIM-DT_X (X = 0, 5, 10, 25, and 100), where x represents the molar fraction of TPB incorporated into the polymer framework.

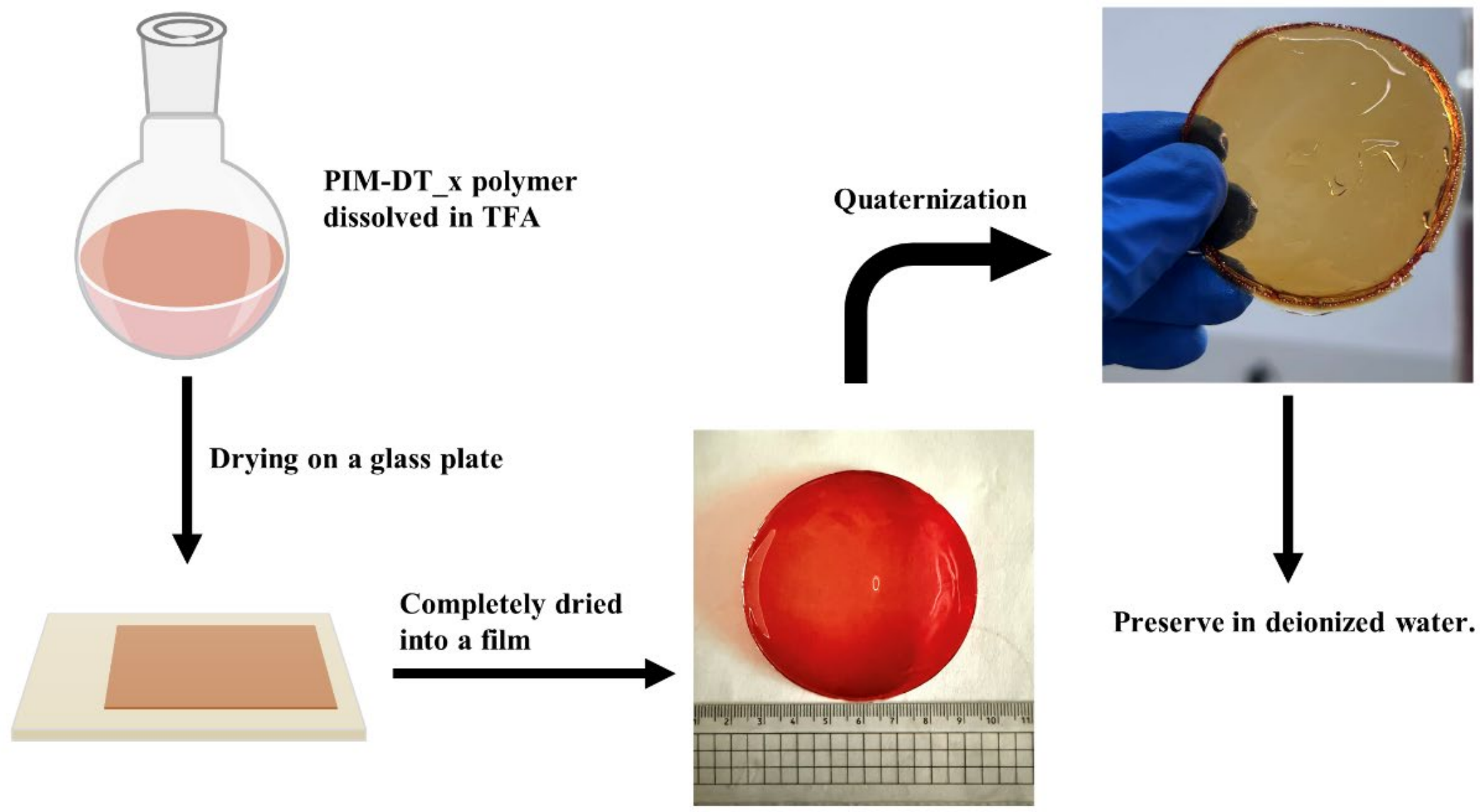

Fig. S3. Post-functionalization process of Q_PIM-DT membranes. PIM-DT-X membranes are dried into a film followed by quaternization process with CH_3I.

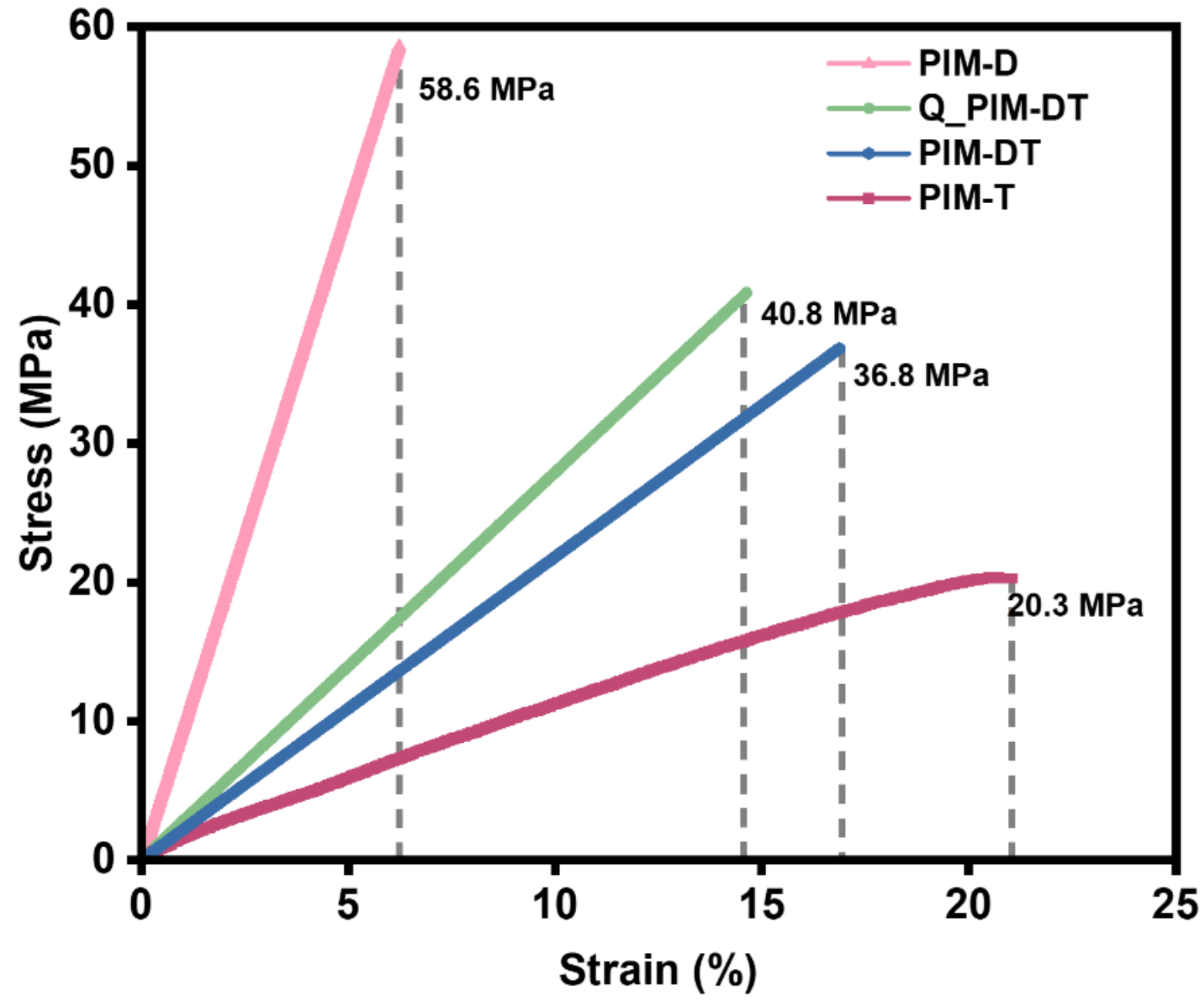

Fig. S4. Mechanical properties of PIM membranes. PIM-D (pink), PIM-T (red), PIM-DT_10 (blue), and Q_PIM-DT_10 (green) membranes.

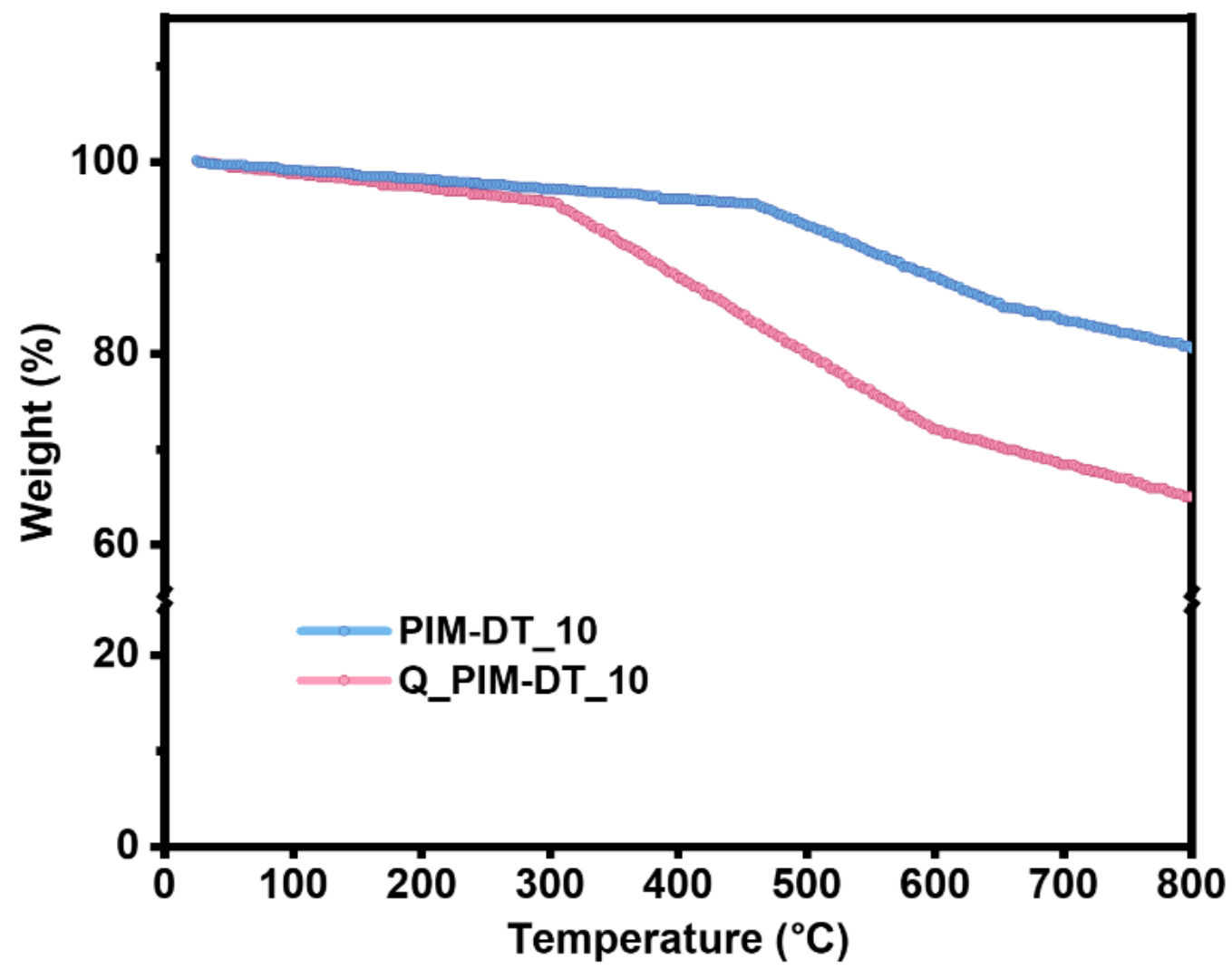

Fig. S5. Thermogravimetric analysis (TGA) curves of PIM membranes. PIM-DT_10 is represented with blue and Q_PIM-DT_10 in pink.

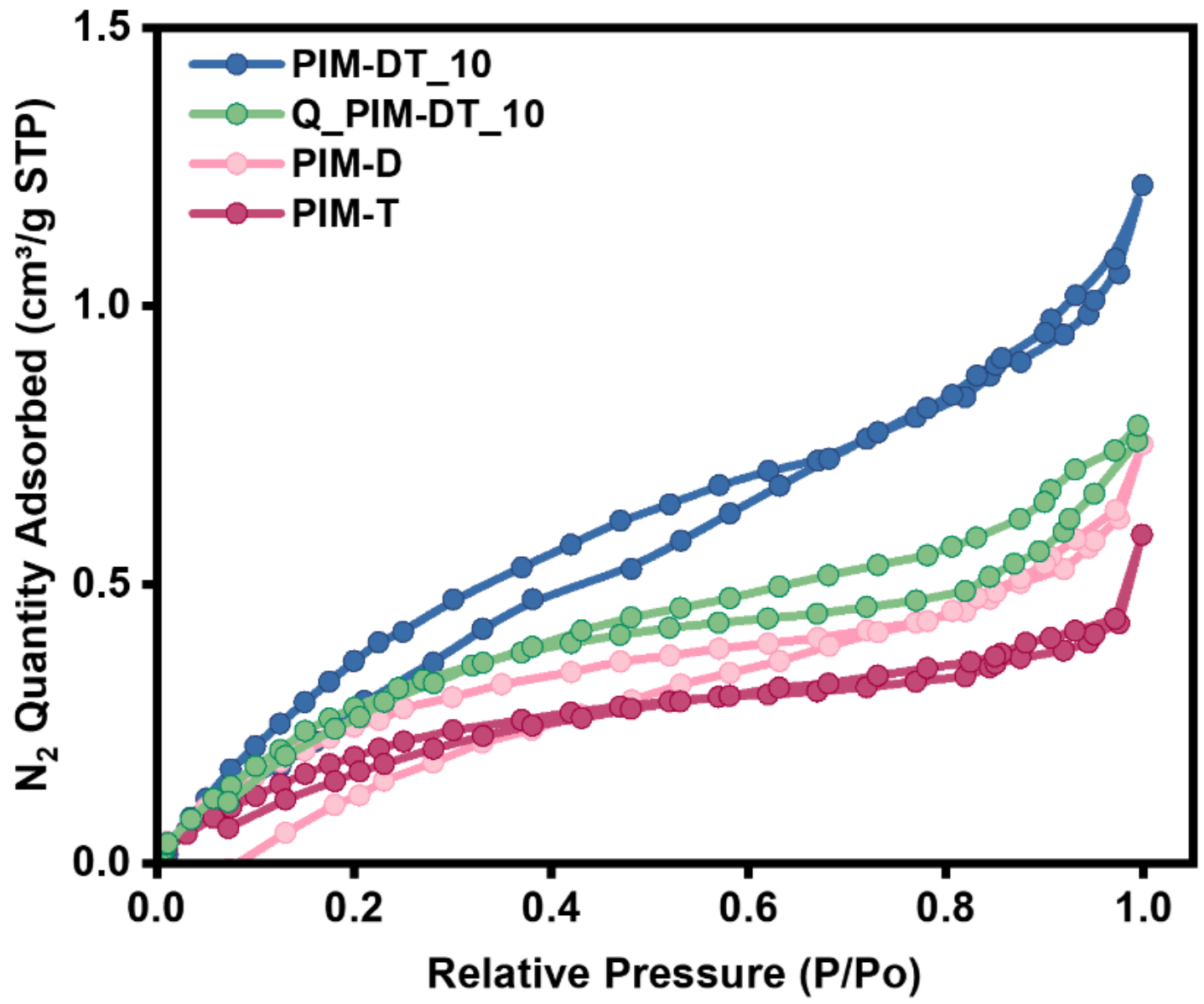

Fig. S6. N_2 sorption isotherms of PIM membranes. PIM-D (pink), PIM-T (red), PIM-DT_10 (blue), and Q_PIM-DT_10 (green) membranes at 77.361 K. N_2 sorption isotherms revealed uptake capacities of 0.76, 1.22, 0.79, and 0.59 $cm^3 \cdot g^{-1}$ STP for PIM-D, PIM-DT_10, Q_PIM-DT_10, and PIM-T, respectively.

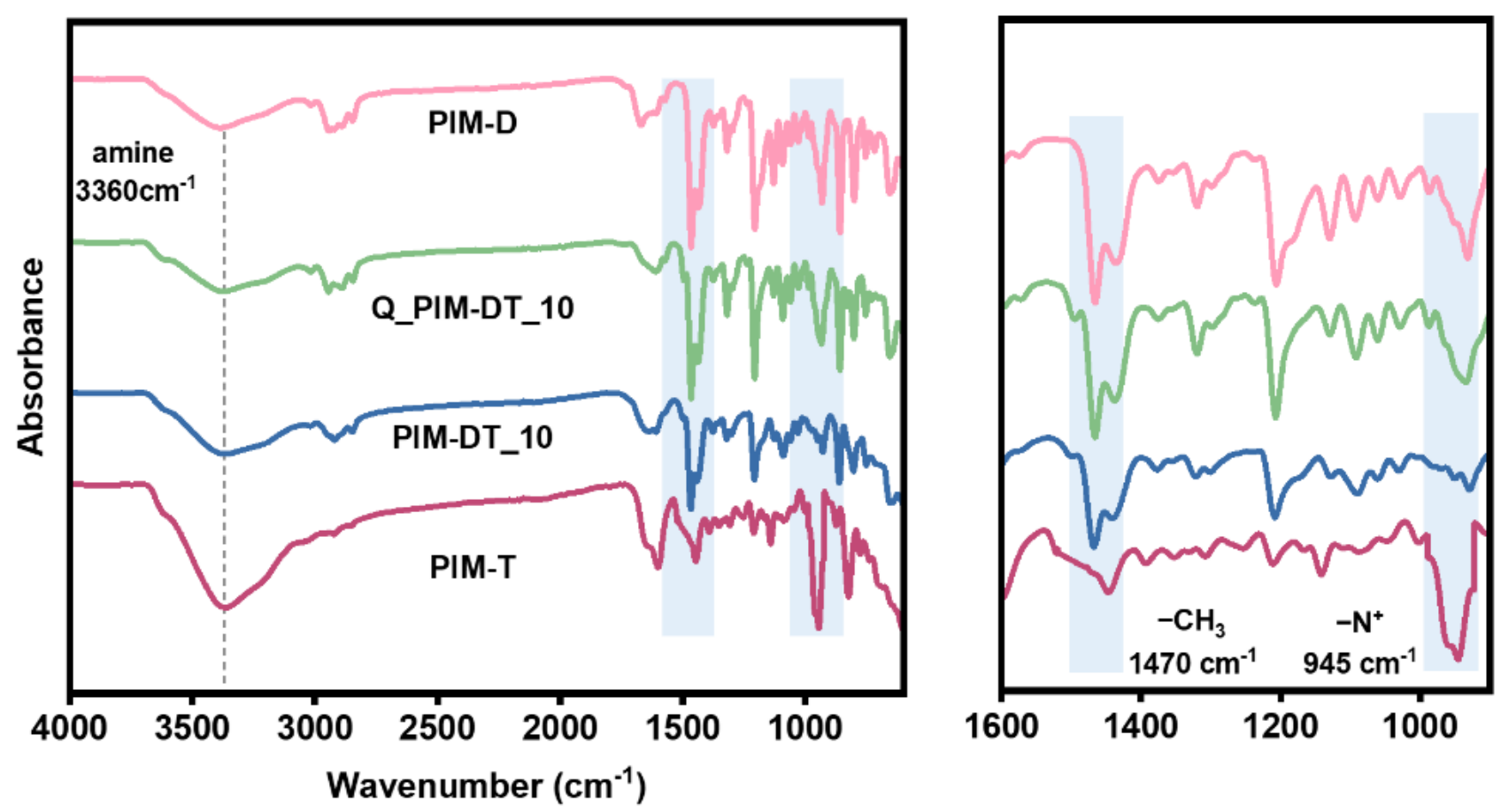

Fig. S7. FTIR spectra of PIM membranes. PIM-D (pink), PIM-T (red), PIM-DT_10 (blue), and Q_PIM-DT_10 (green) membranes. Characteristic absorption peaks at 945 cm^{-1} and 3360 cm^{-1} were assigned to the quaternary amine bond, trisubstituted benzene ring vibrations, and N–H stretching bands from the TPB units, respectively. In addition, the intensity of a new band at ~1470 cm^{-1}—corresponding to the bending vibration of methyl groups ($–CH_3$) from the DMBP monomer—gradually decreased with increasing TPB content, consistent with the designed DMBP/TPB monomer ratios.

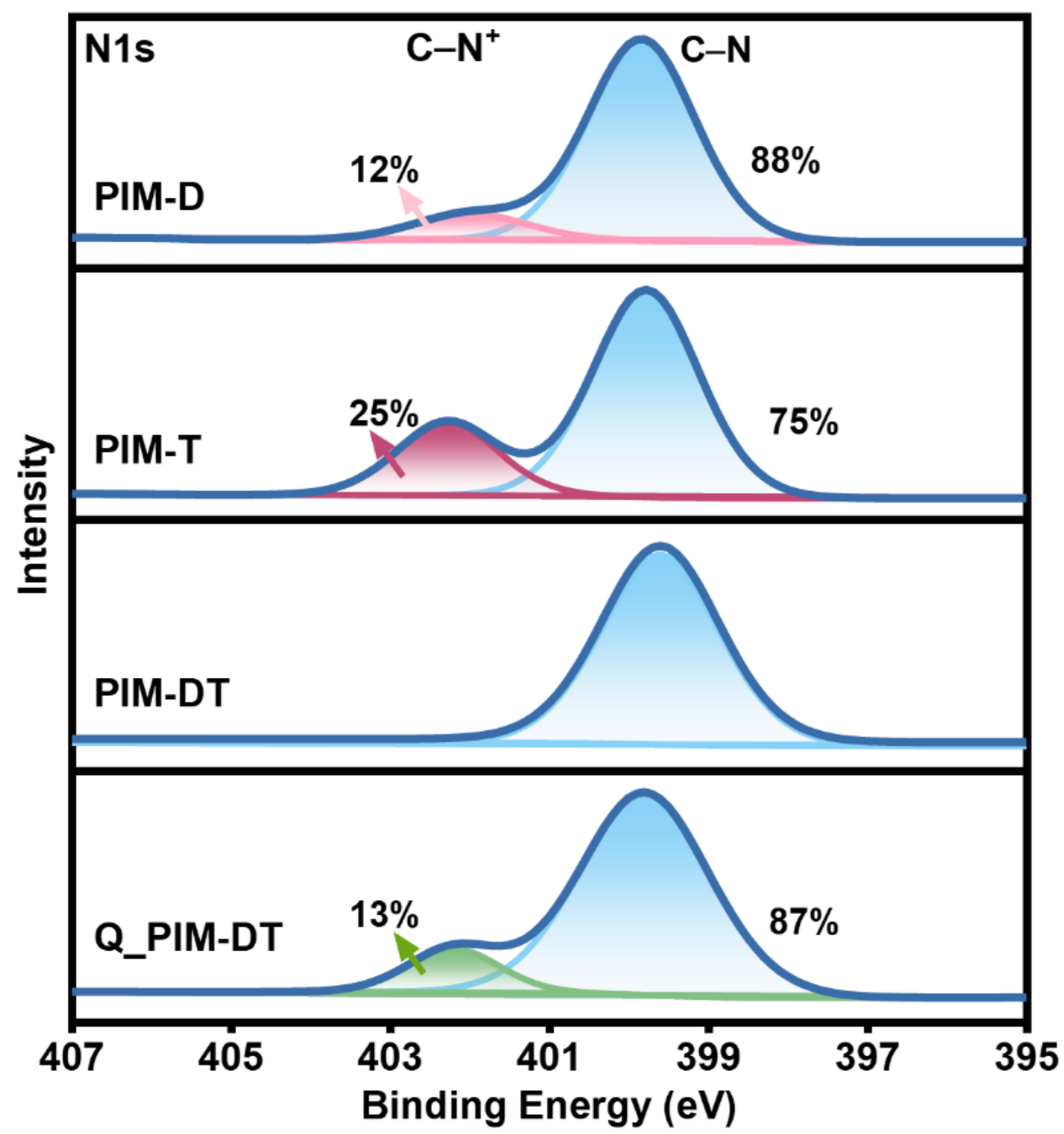

Fig. S8. N 1s core-level XPS spectra of TB-based POP membranes. Nitrogen species are highlighted as they are associated with the transformation of quaternary ammonium group. The Q_PIM-DT_10 membrane exhibited a pronounced XPS N 1s peak at 402.17 eV, characteristic of quaternary ammonium (–N^+) species, confirming successful quaternization and the introduction of positive charge relative to the pristine PIM-DT_10 membrane. Furthermore, systematic variations in the –N^+ peak intensity across the series correlate with the designed DMBP/TPB ratios, verifying controlled incorporation of quaternary ammonium groups during synthesis.

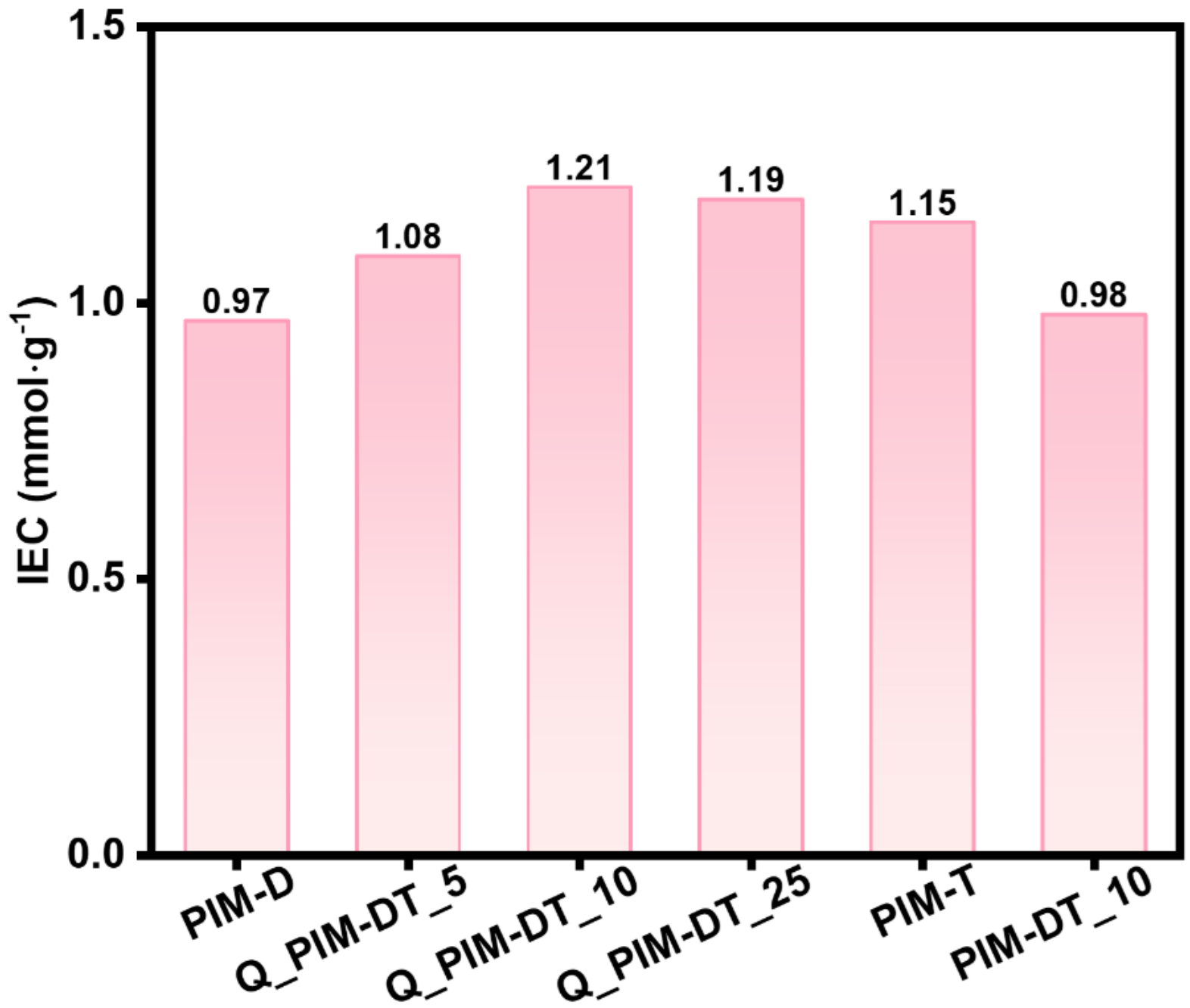

Fig. S9. Ion exchange capacity (IEC) of PIM membranes. IEC of Q_PIM-DT_X membranes with varying TPB molar fractions are reported.

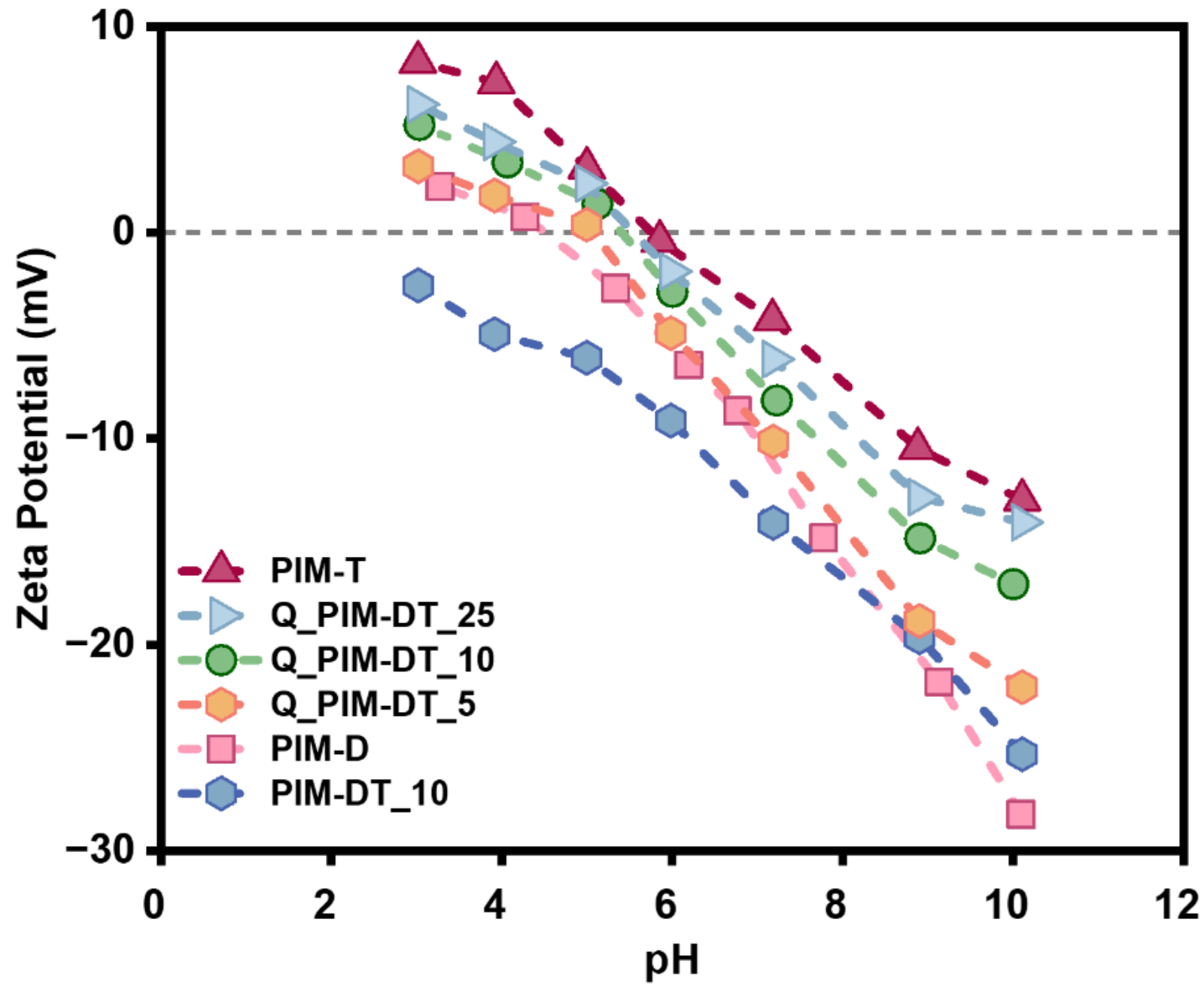

Fig. S10. Zeta Potentials of PIM membranes. PIM-D (pink), PIM-T (red), PIM-DT_10 (blue), and Q_ PIM-DT _5 (orange), Q_ PIM-DT _10 (green), Q_ PIM-DT _25 (light blue) membranes at different pH values. The Q_ PIM-DT membranes exhibited markedly higher positive surface potentials than their unquaternized counterparts, with ζ-potentials of +5.21 mV for Q_ PIM-DT _10 and −2.56 mV for PIM-DT _10 at pH 3. This charge reversal confirms the successful introduction of quaternary ammonium groups, which substantially increase the density of fixed cationic sites. Moreover, the magnitude of the positive potential increases with higher crosslinking degrees, reflecting the enhanced incorporation of ionized quaternary functionalities within the network.

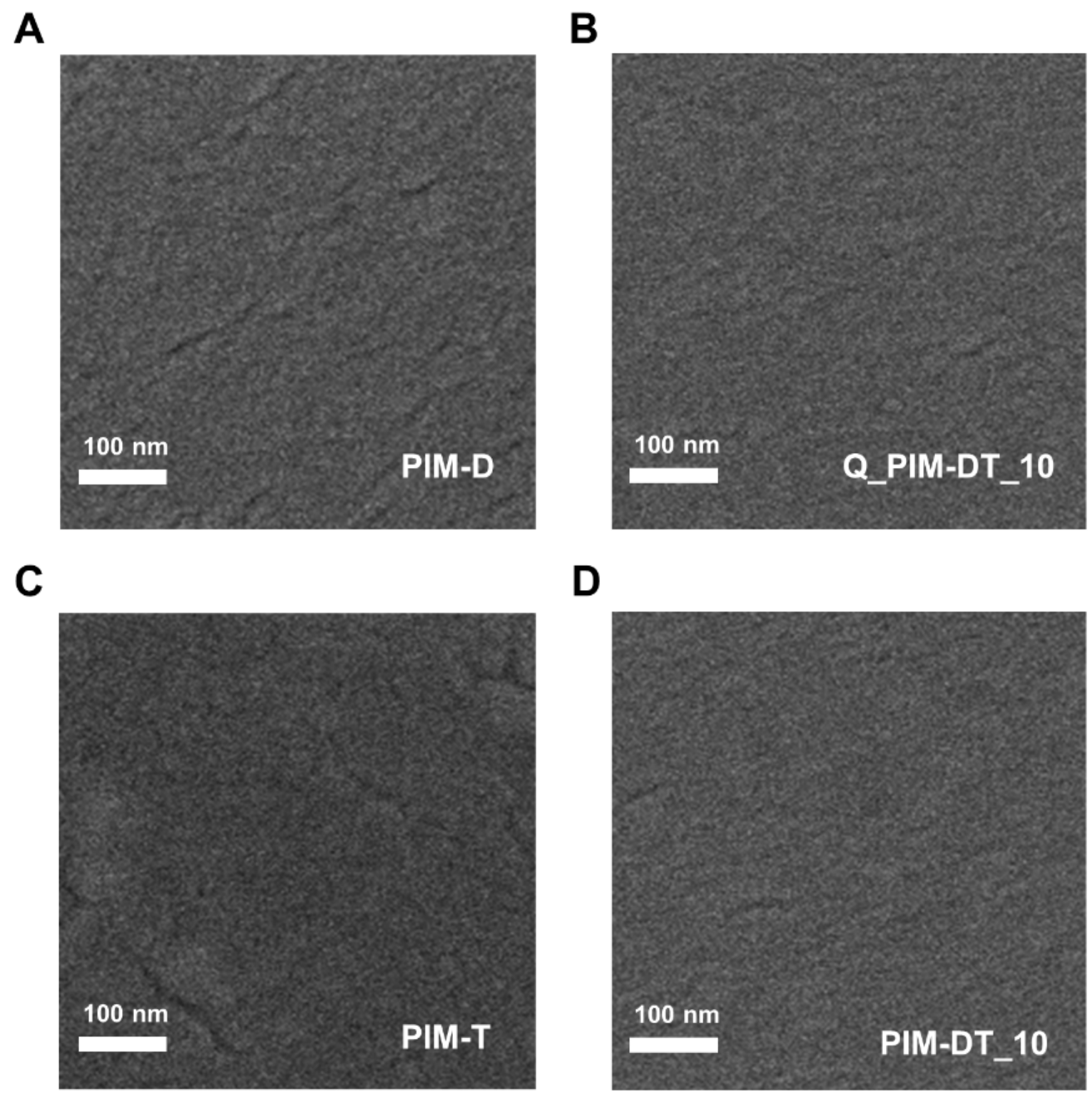

Fig. S11. SEM analysis of surface morphology in TB-based POP membranes. (**A)** SEM image of the surface morphology of PIM-D. (**B)** Q_PIM-DT_10. **(C)** PIM-T. (**D)** PIM-DT_10.

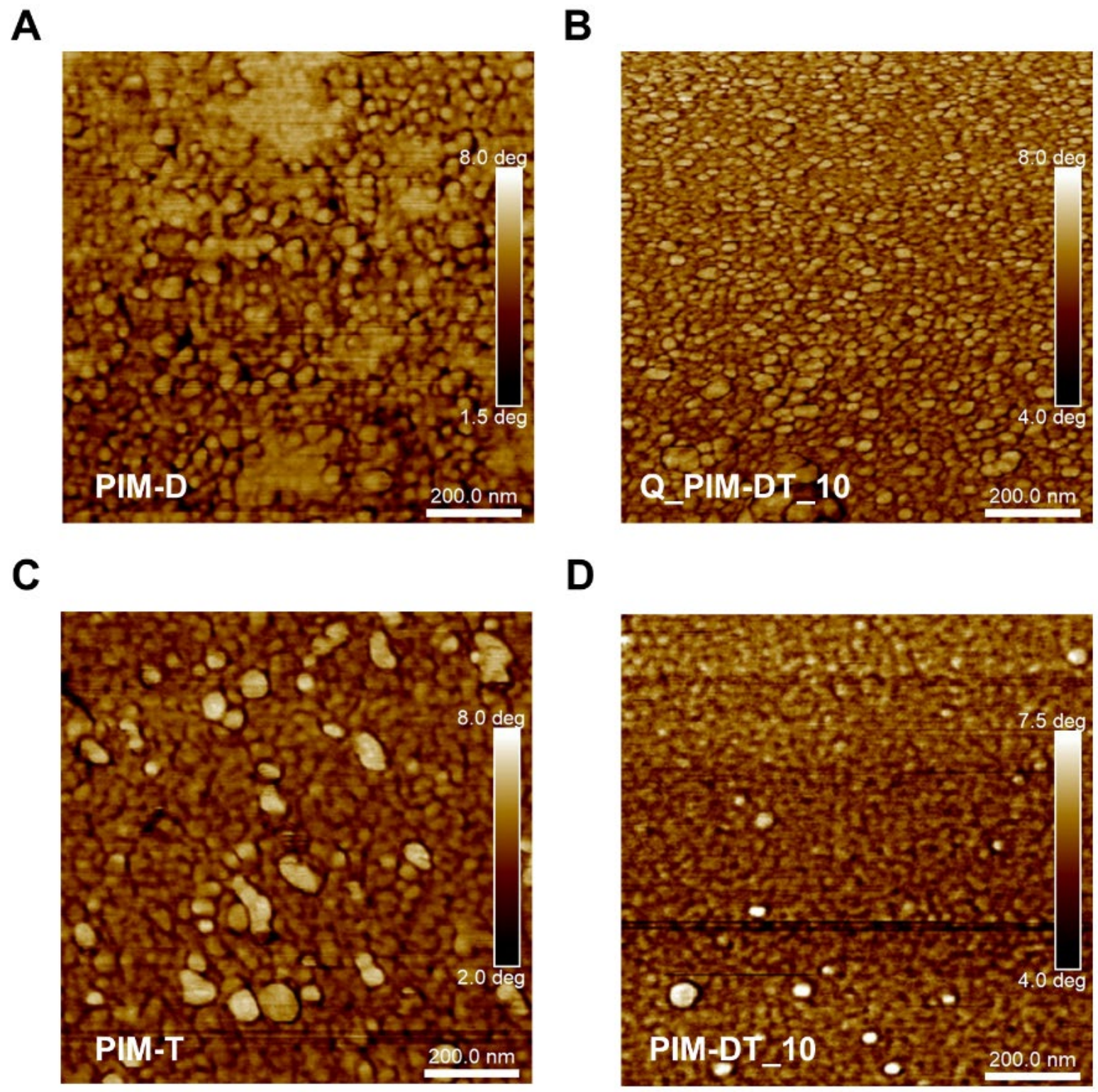

Fig. S12. AFM phase analysis of surface morphology in TB-based POP membranes. (**A)** AFM phase image of the surface morphology of PIM-D. (**B)** Q_PIM-DT_10. (**C)** PIM-T. (**D)** PIM-DT_10.

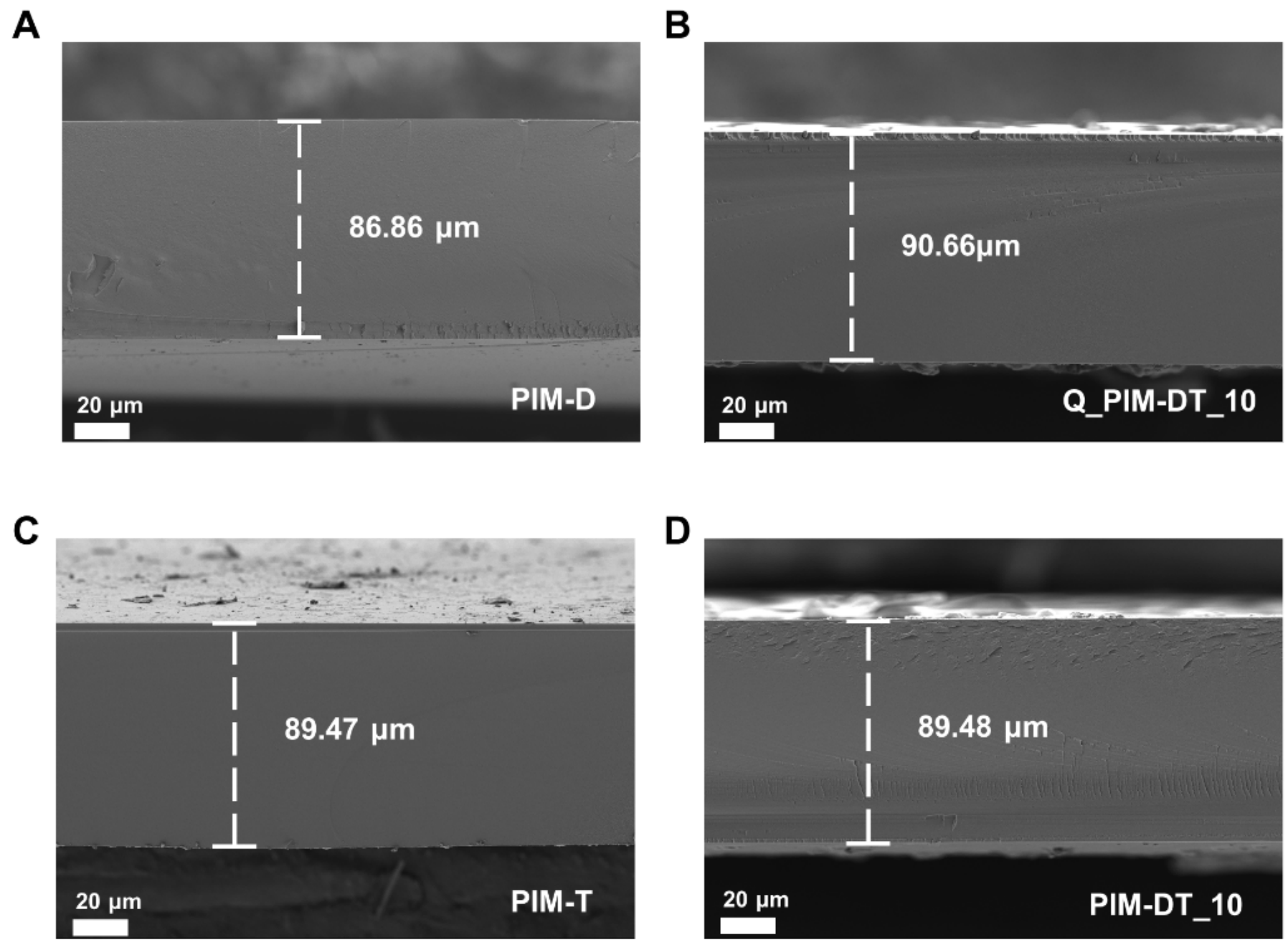

Fig. S13. Cross-sectional SEM analysis of TB-based POP membranes. (**A**) Cross-sectional SEM image of PIM-D. (**B**) Q_PIM-DT_10. (**C**) PIM-T. (**D**) PIM-DT_10.

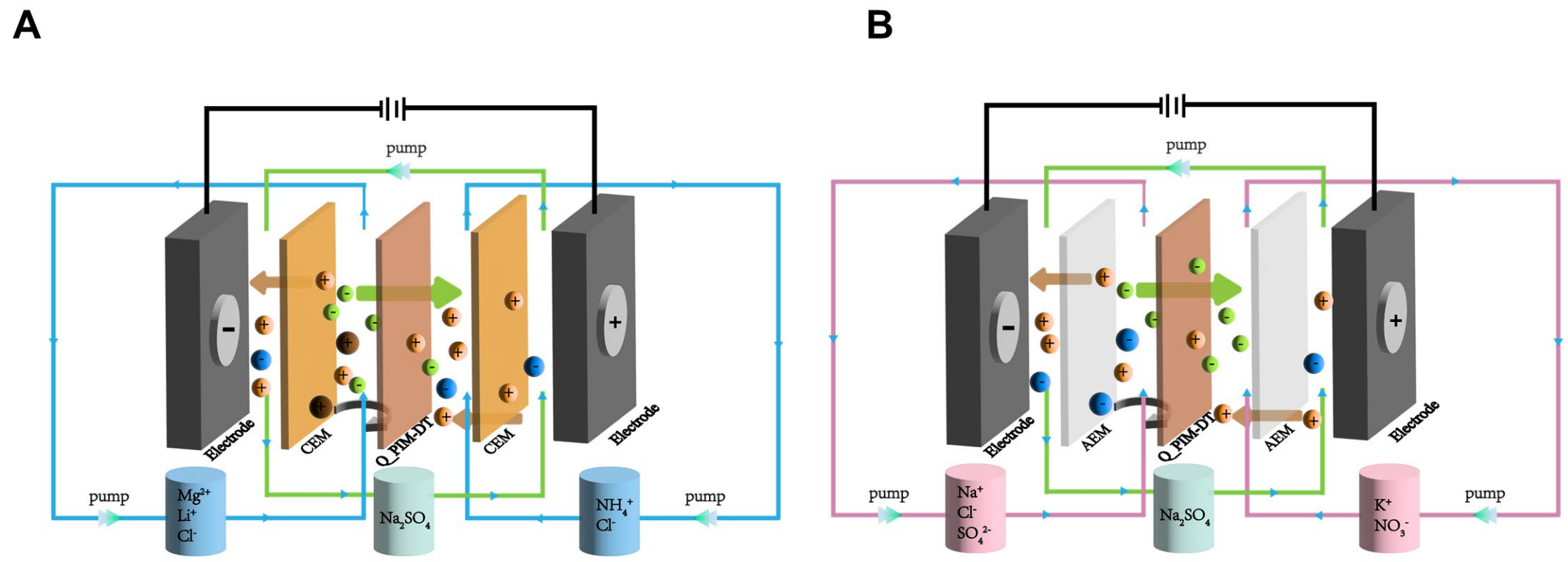

Fig. S14. Schematic illustrations of different electrodialysis stack configurations used for (A) monovalent-anion separation tests and (B) monovalent-cation separation tests.

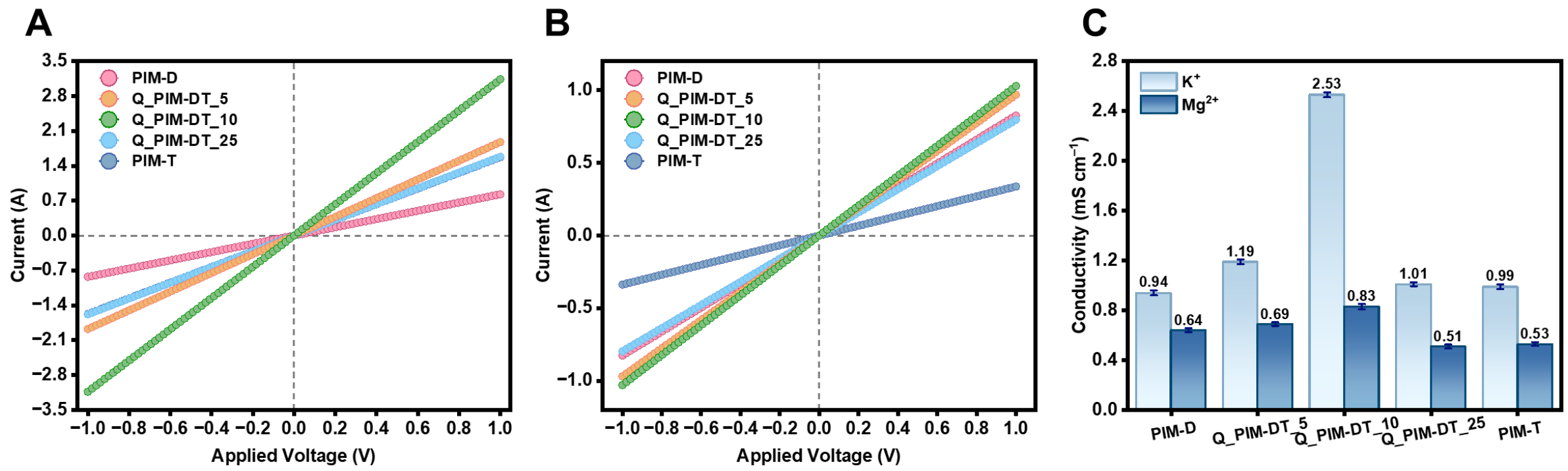

Fig. S15. Ion transport characteristics of TB-based POP membranes in mono- and divalent sulfate-based electrolytes. (**A-B)** Current-voltage (I-V) curves of Q_PIM-DT_X membranes measured in 0.5 M K_2SO_4 **(A)** and 0.5 M $MgSO_4$ **(B),** revealing distinct transport responses for monovalent versus divalent cations. **(C)** Corresponding cation conductivities of the different membranes derived from the I-V measurements.

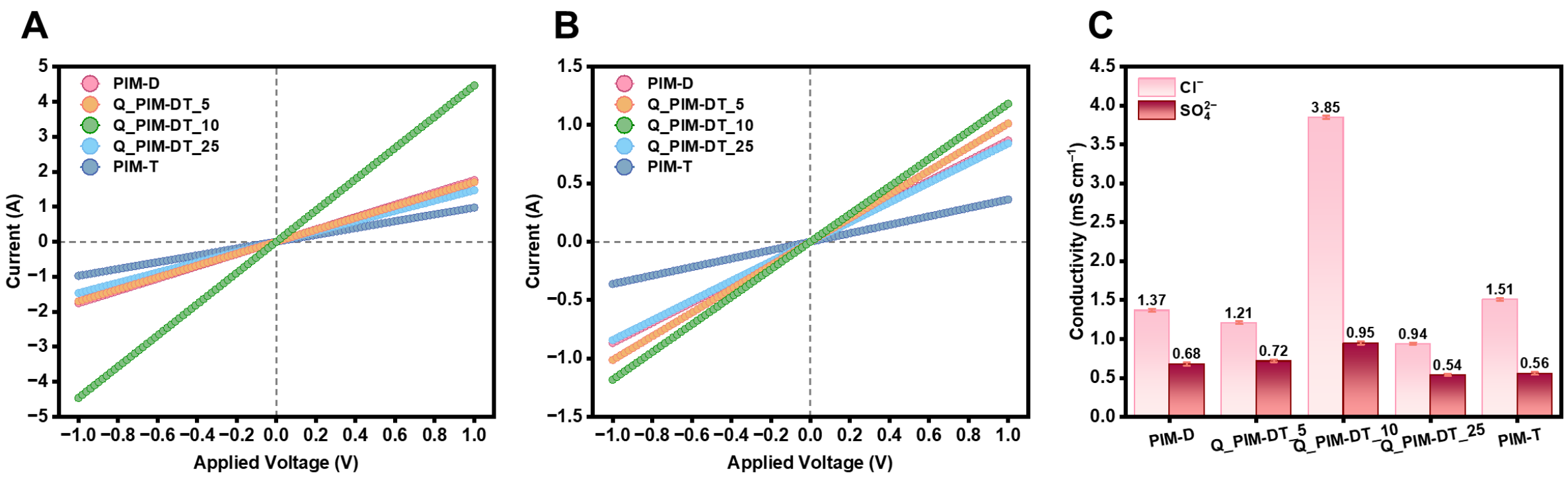

Fig. S16. Ion transport characteristics of TB-based POP membranes in monovalent and divalent sodium-based electrolytes. (**A-B**) Current-voltage (I-V) curves of Q_PIM-DT_X membranes measured in 0.5 M NaCl **(A)** and 0.5 M Na_2SO_4 **(B),** revealing distinct transport responses for monovalent (Cl^-) versus divalent (SO_4^{2-}) anions. (**C**) Corresponding anion conductivities of the different membranes derived from the I-V measurements.

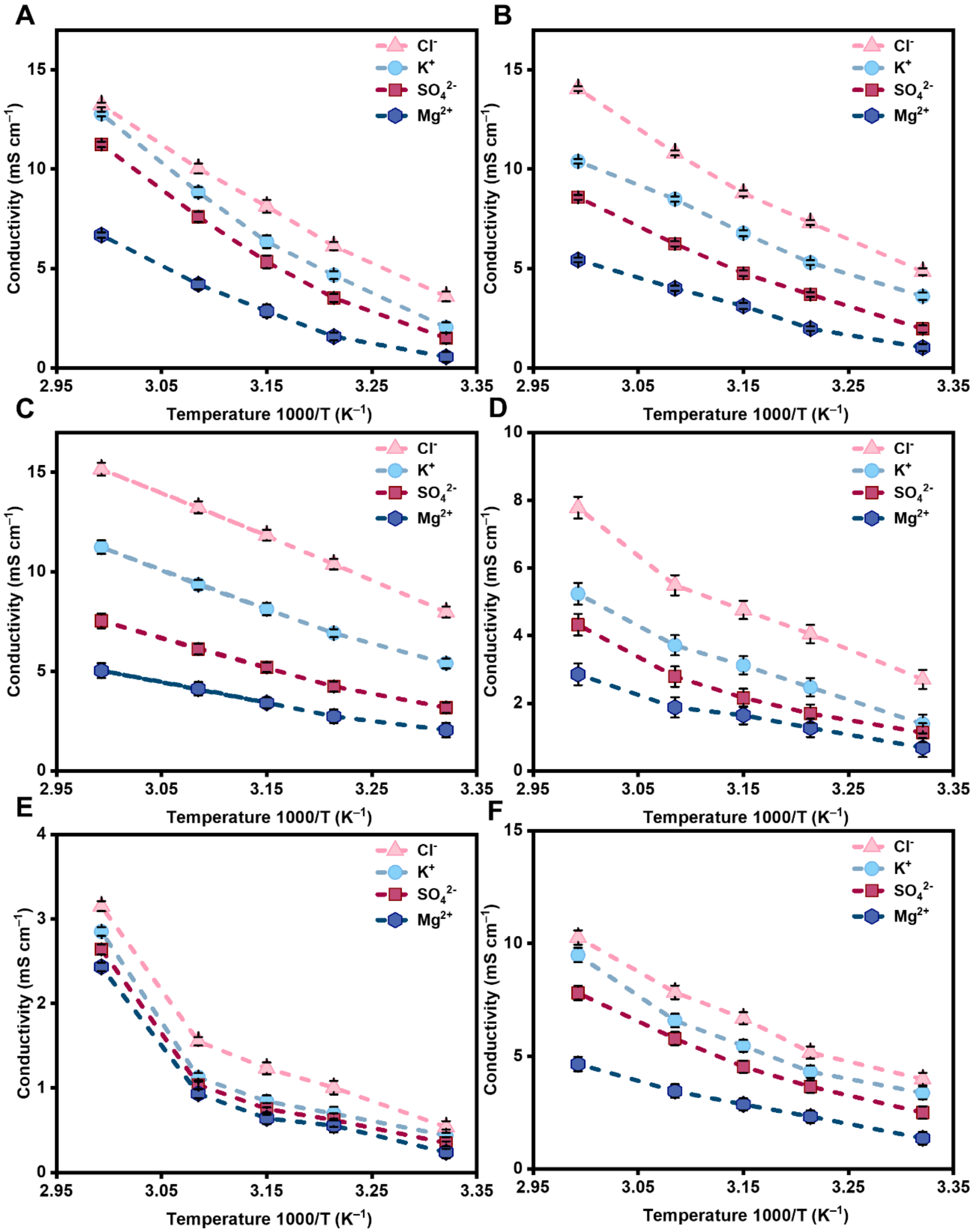

Fig. S17. Temperature-dependent ionic conductivity of TB-based POP membranes in representative mono-/multivalent electrolytes. (**A–F**) Ionic conductivity as a function of temperature for pristine and pretreated membranes, including PIM-D **(A),** Q_PIM-DT_5 **(B),** Q_PIM-DT_10 **(C),** Q_PIM-DT_25 **(D),** PIM-T **(E),** and PIM-DT_10 **(F),** measured in 0.1 M KCl, $MgCl_2$, NaCl, and Na_2SO_4 solutions.

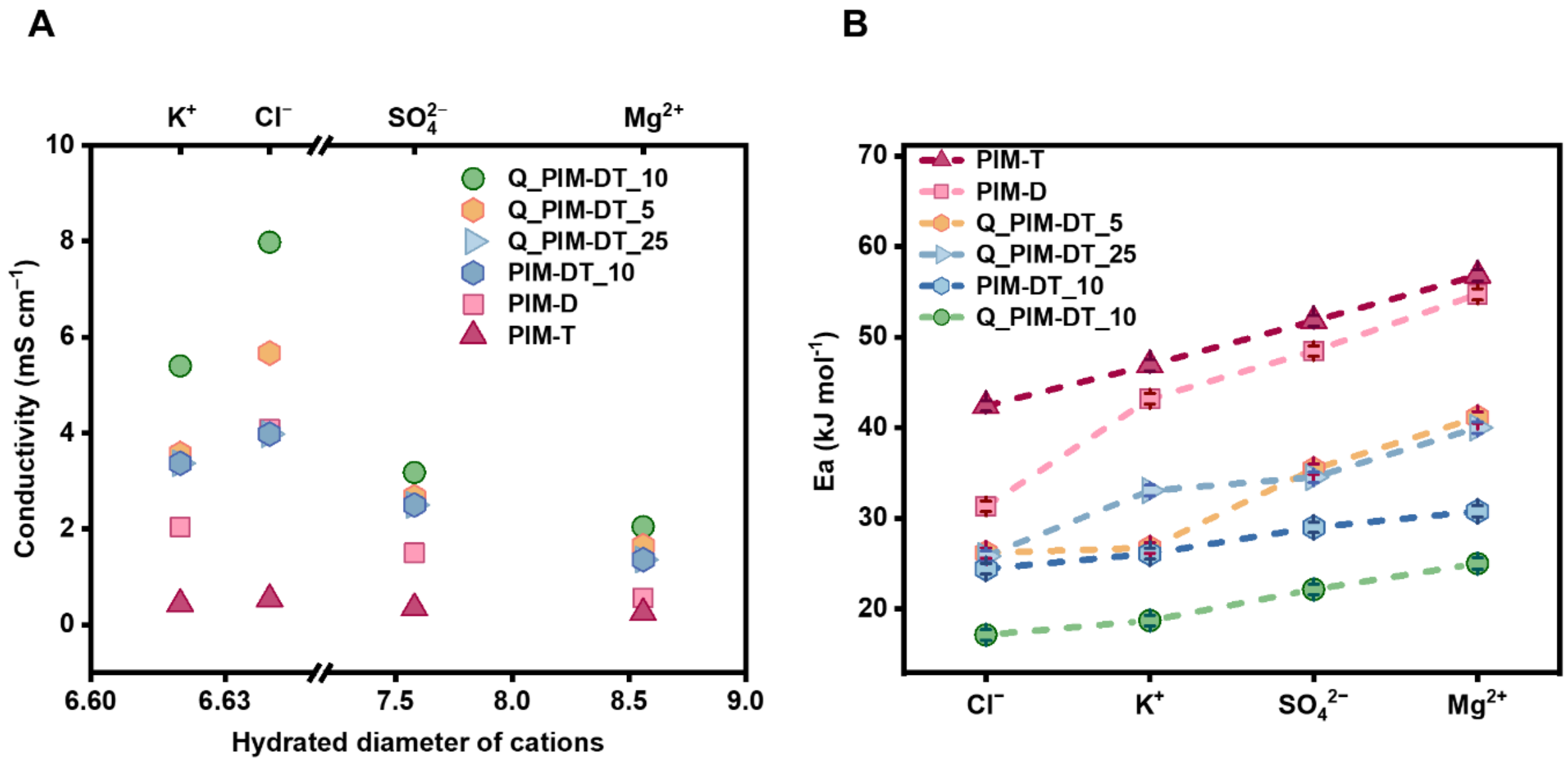

Fig. S18. Ion-size-dependent transport and activation energetics in TB-based POP membranes. (**A)** Ionic conductivity of Q_PIM-DT_X membranes as a function of hydrated ion diameter, measured in 0.1 M KCl, $MgCl_2$, NaCl, and Na_2SO_4 at 30 °C. (**B)** Apparent transmembrane activation energies for ion transport across TB-based POP membranes extracted from temperature-dependent conductivity data.

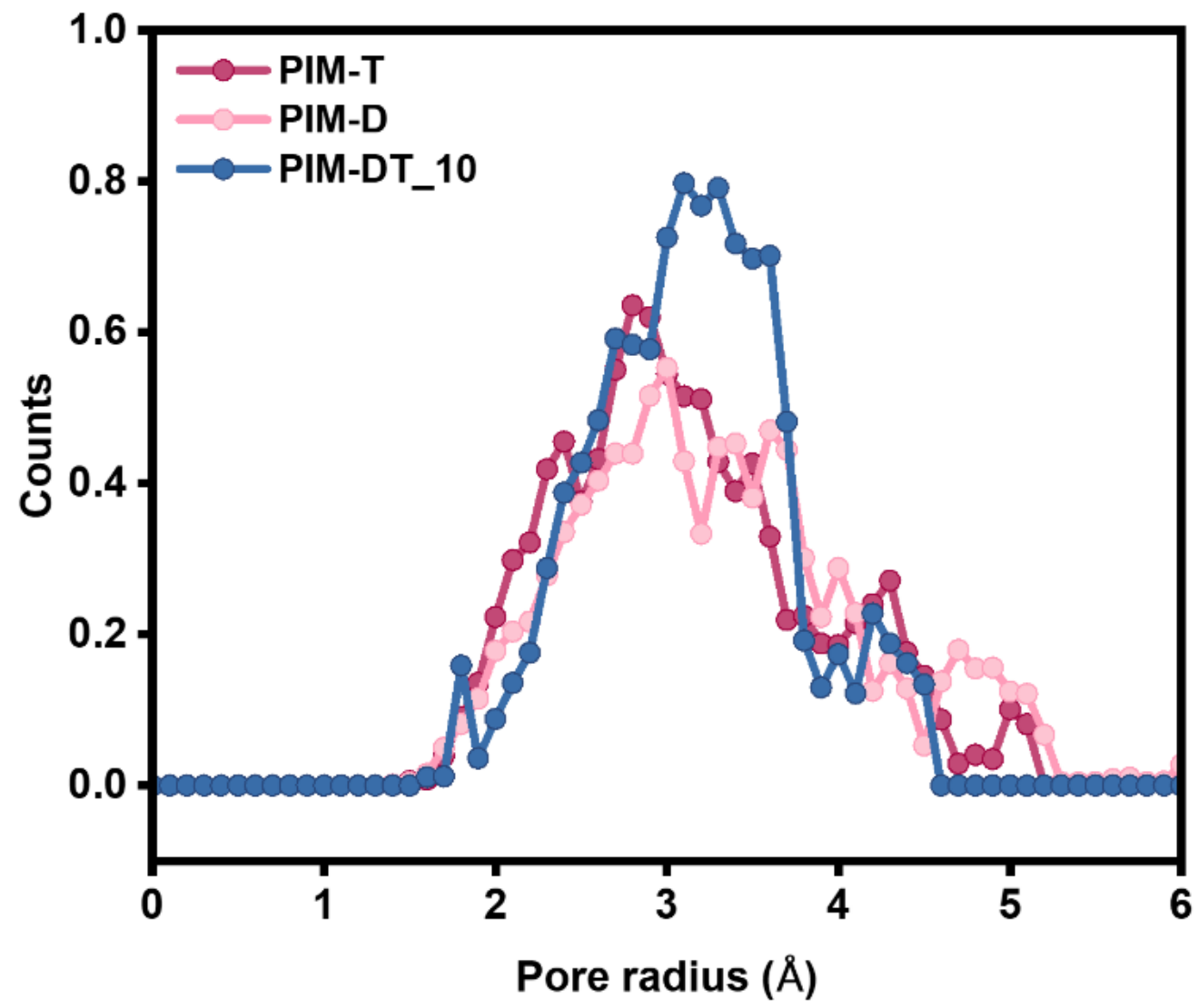

Fig. S19. Simulated pore size distribution of PIMs. Pore size intensity simulations of PIM-T (red), PIM-D (pink), and PIM-DT_10 (blue) networks.

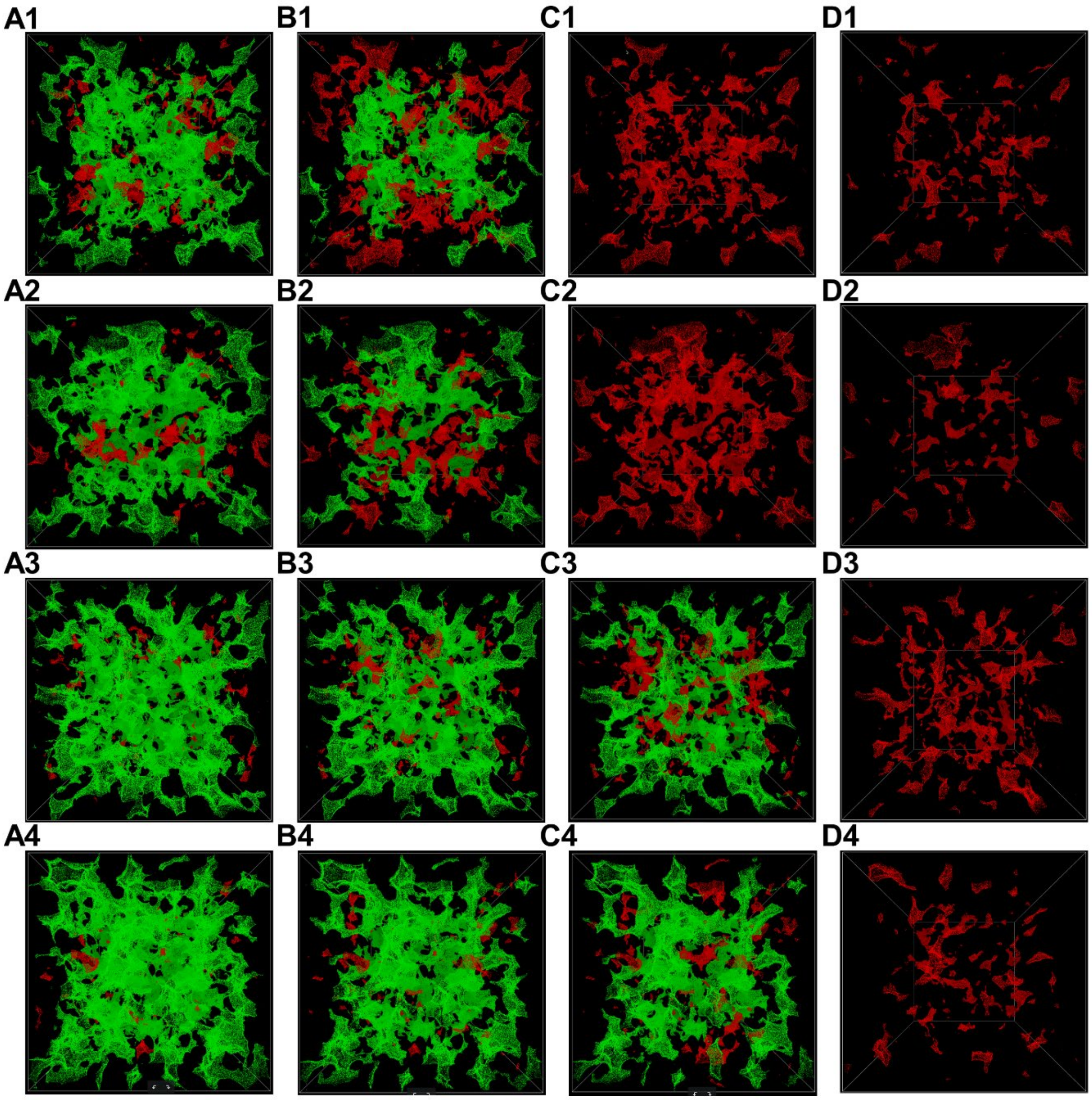

Fig. S20. Probe-size-dependent void connectivity in TB-based POP network models. (A1–D1) Interconnected (green) and disconnected (red) void networks in PIM-T identified using spherical probes with radii of 0.8, 0.9, 1.0, and 1.2 Å **(columns A–D).** (**A2–D2)** Corresponding void connectivity maps for PIM-D. (**A3–D3)** Corresponding maps for PIM-DT_10. (**A4–D4)** Corresponding maps for Q_PIM-DT_10, highlighting how increasing probe size progressively restricts accessible interconnected pathways across different network chemistries.

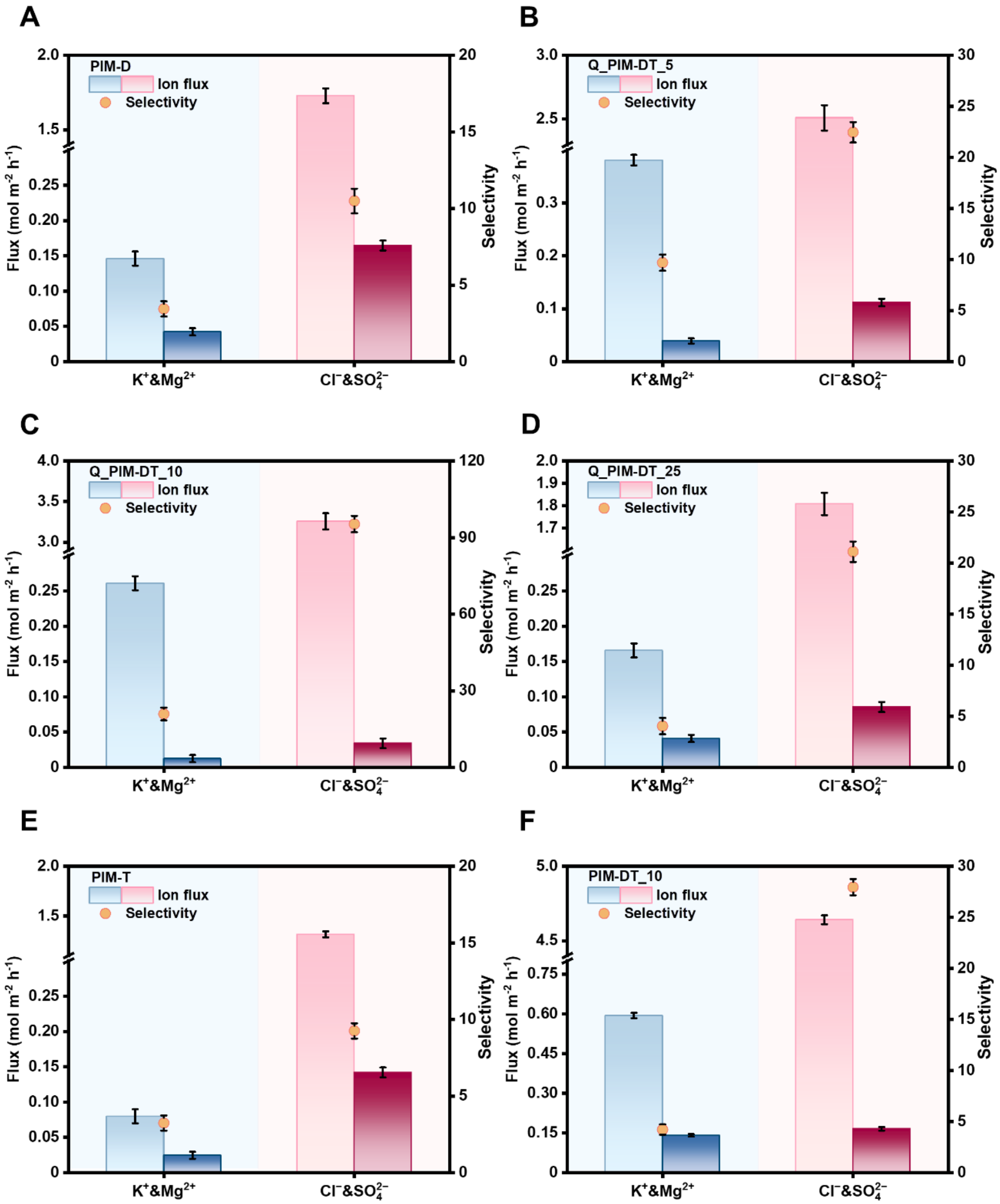

Fig. S21. K^+&Mg^{2+}, Cl^-&SO_4^{2-} fluxes and selectivities of TB-based POP membranes at 5 mA·cm^{-2}. (**A**) PIM-D. (**B**) Q_PIM-DT_5. (**C**) Q_PIM-DT_10. (**D**) Q_PIM-DT_25. (**E**) PIM-T. (**F**) PIM-DT_10. Bar plots show the individual ion fluxes for the K^+/Mg^{2+} and Cl^-/SO_4^{2-} pairs, and symbols denote the corresponding selectivity ratios.

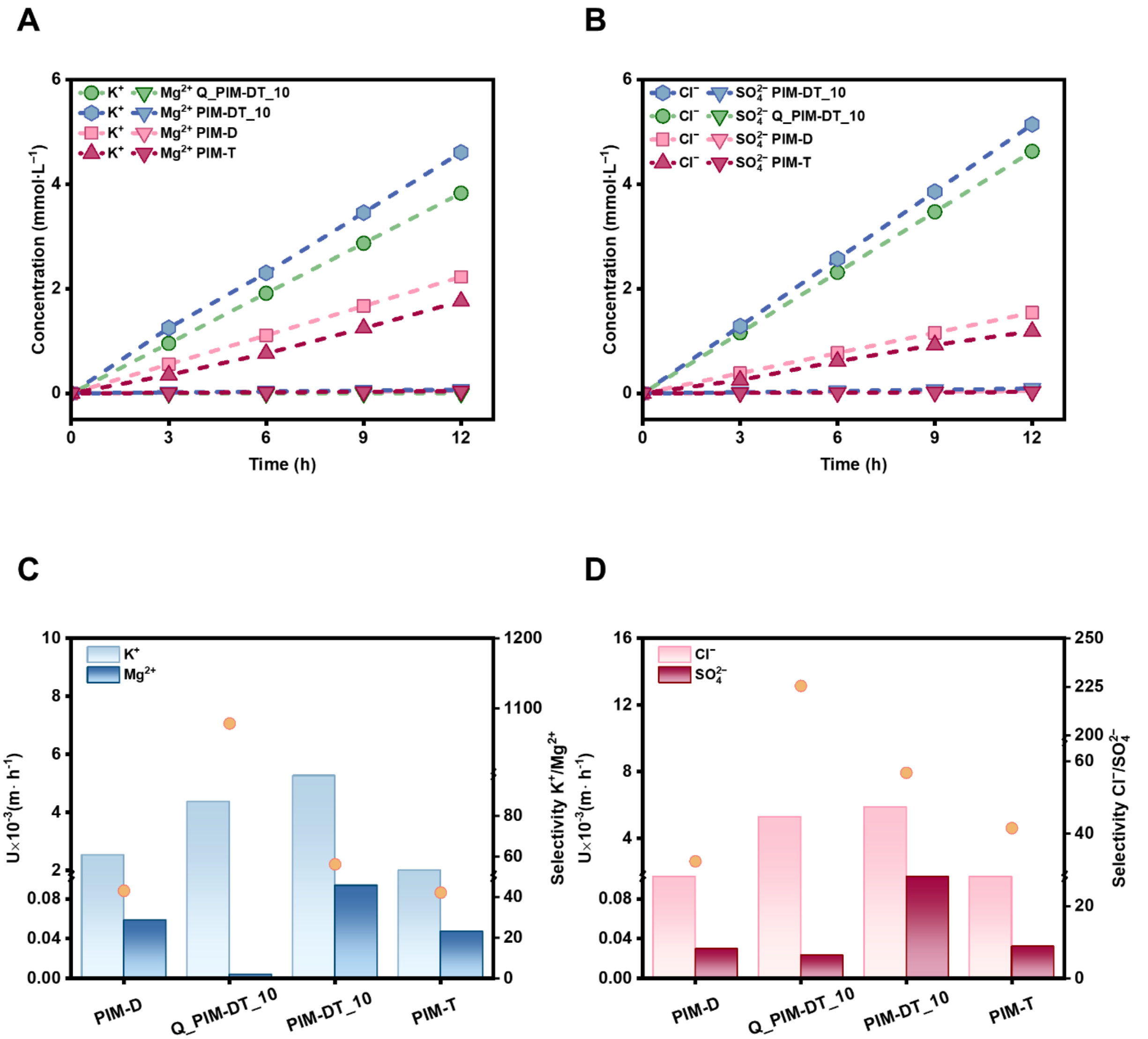

Fig. S22. Time-dependent permeability and mono-/multivalent ion discrimination in TB-based POP membranes. (**A–B**) Time-dependent ion concentrations in the receiving chamber for selected **(A)** cations (K^+ and Mg^{2+}) and **(B)** anions (Cl^- and SO_4^{2-}); the slopes indicate permeability differences across the TB-based POP membranes. (**C**) Comparison of K^+ and Mg^{2+} fluxes (J_K^+ and J_Mg^{2+}) and the corresponding K^+/Mg^{2+} selectivity, (**D**) and comparison of Cl^- and SO_4^{2-} fluxes (J_Cl^- and $J\_SO_4^{2-}$) and the corresponding Cl^-/SO_4^{2-} selectivity among the TB-based POP membranes.

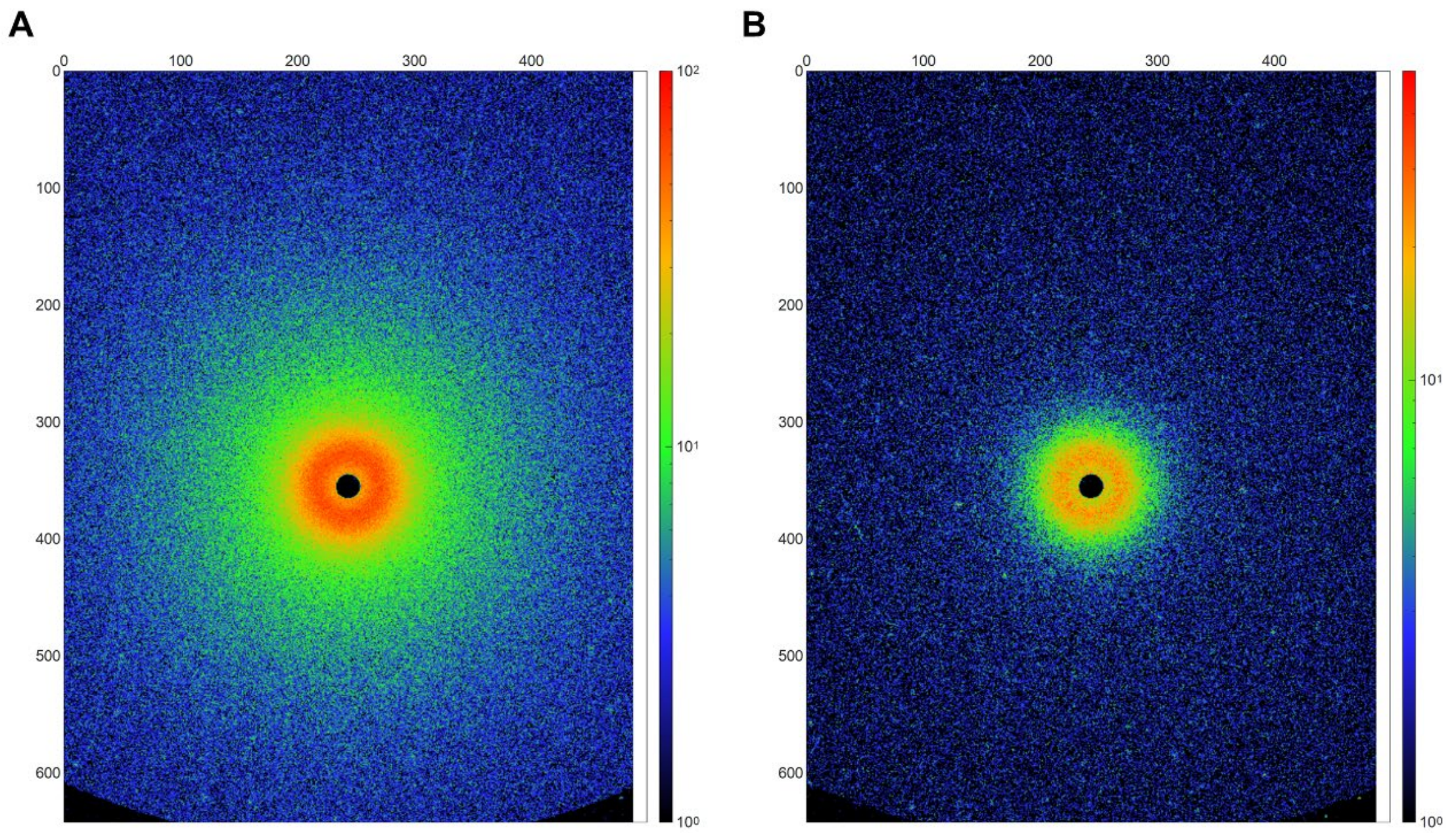

Fig. S23. SAXS two-dimensional patterns of PIM. (**A**) PIM-DT_10 and (**B**) Q_PIM-DT_10.

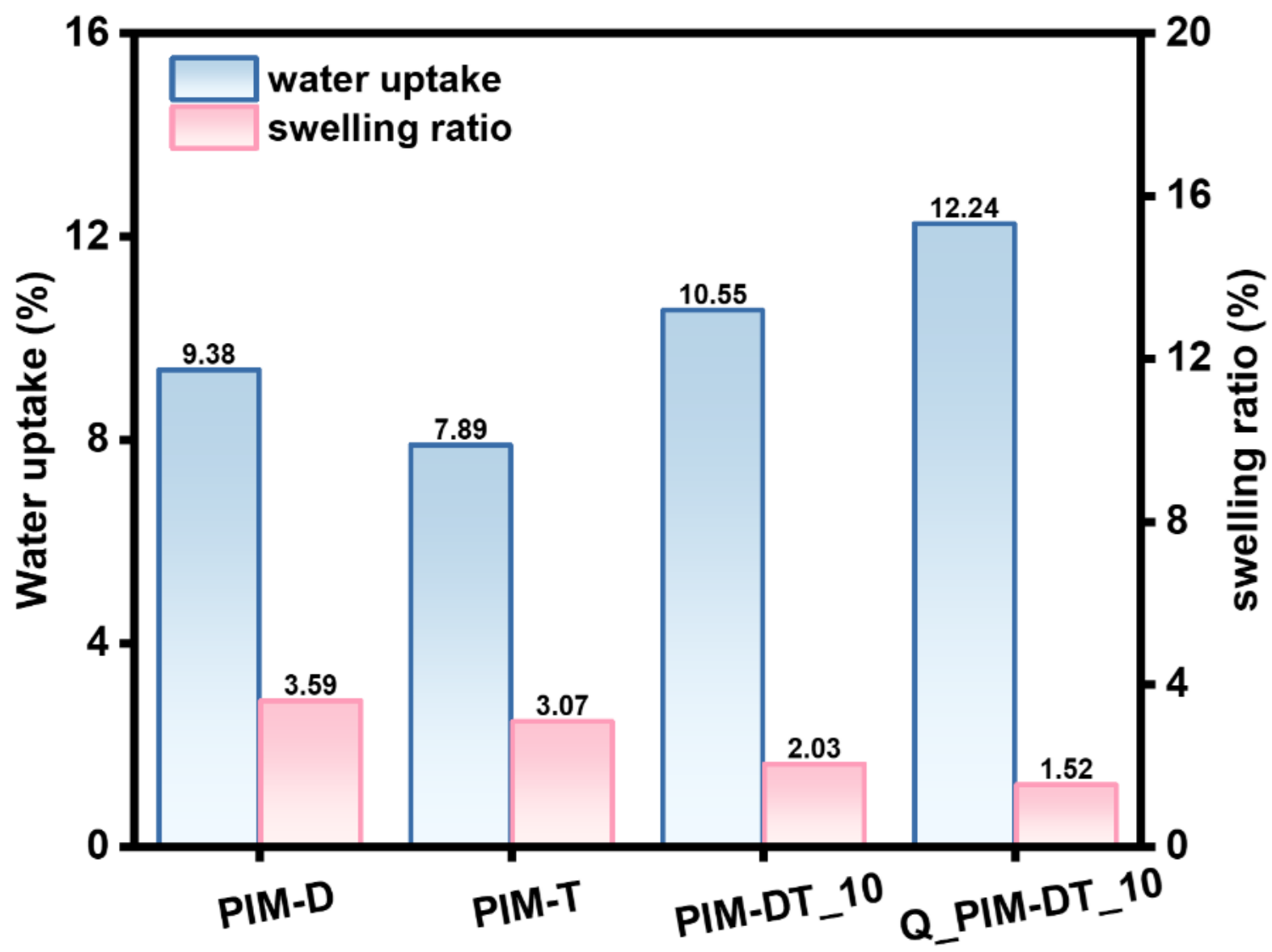

Fig. S24. Water uptake and swelling ratio of PIMs. PIM-D, PIM-T, PIM-DT_10, and Q_PIM-DT_10 membranes.

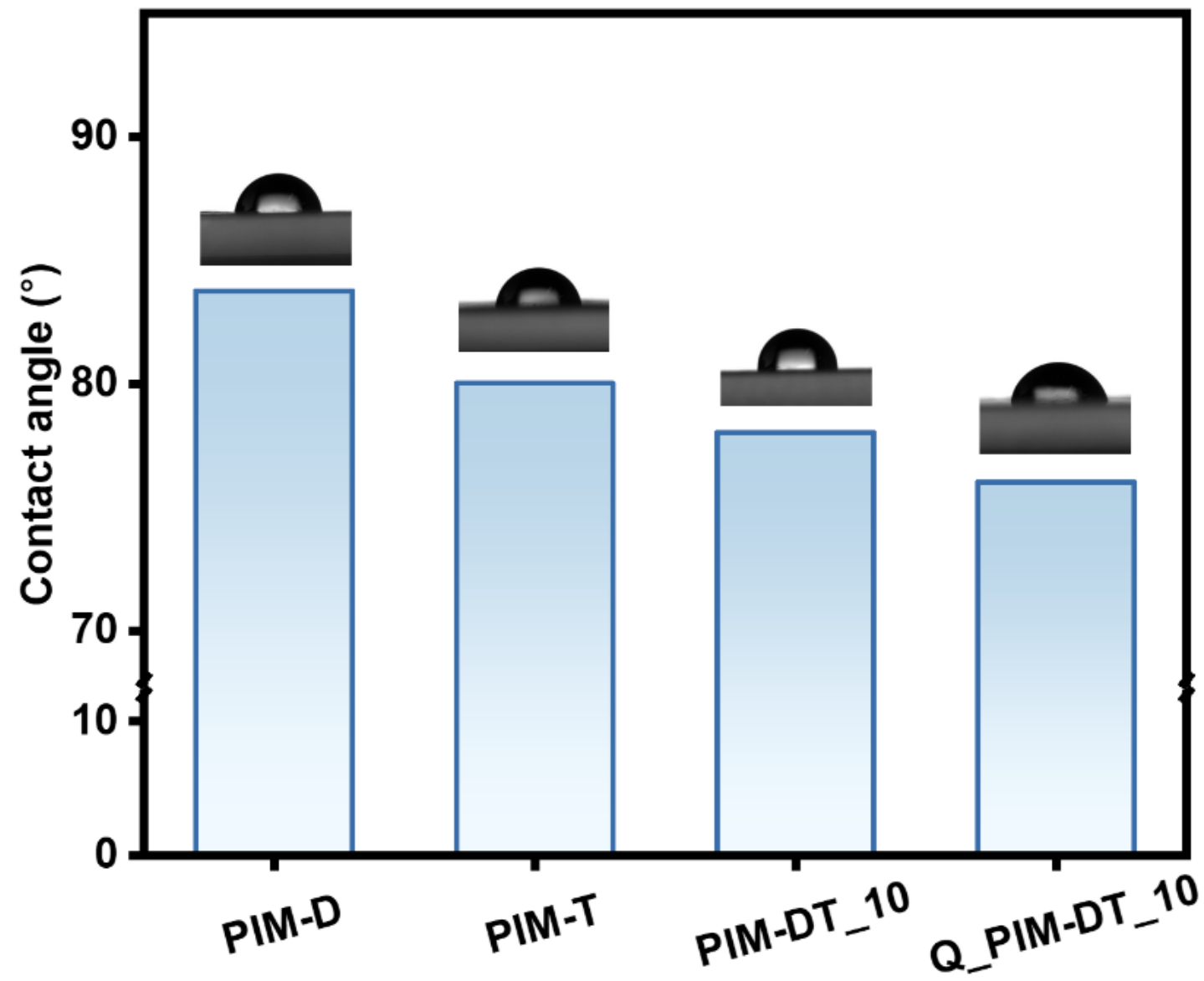

Fig. S25. Water contact angles of PIM-D, PIM-T, PIM-DT_10, and Q_PIM-DT_10 membranes. The water contact angle of PIM-DT membranes varied progressively from 78° for the pristine PIM-DT _10 to 83.7° for PIM-D, 80° for PIM-T, and 76° for Q_ PIM-DT _10, indicating enhanced surface hydrophilicity upon quaternization.

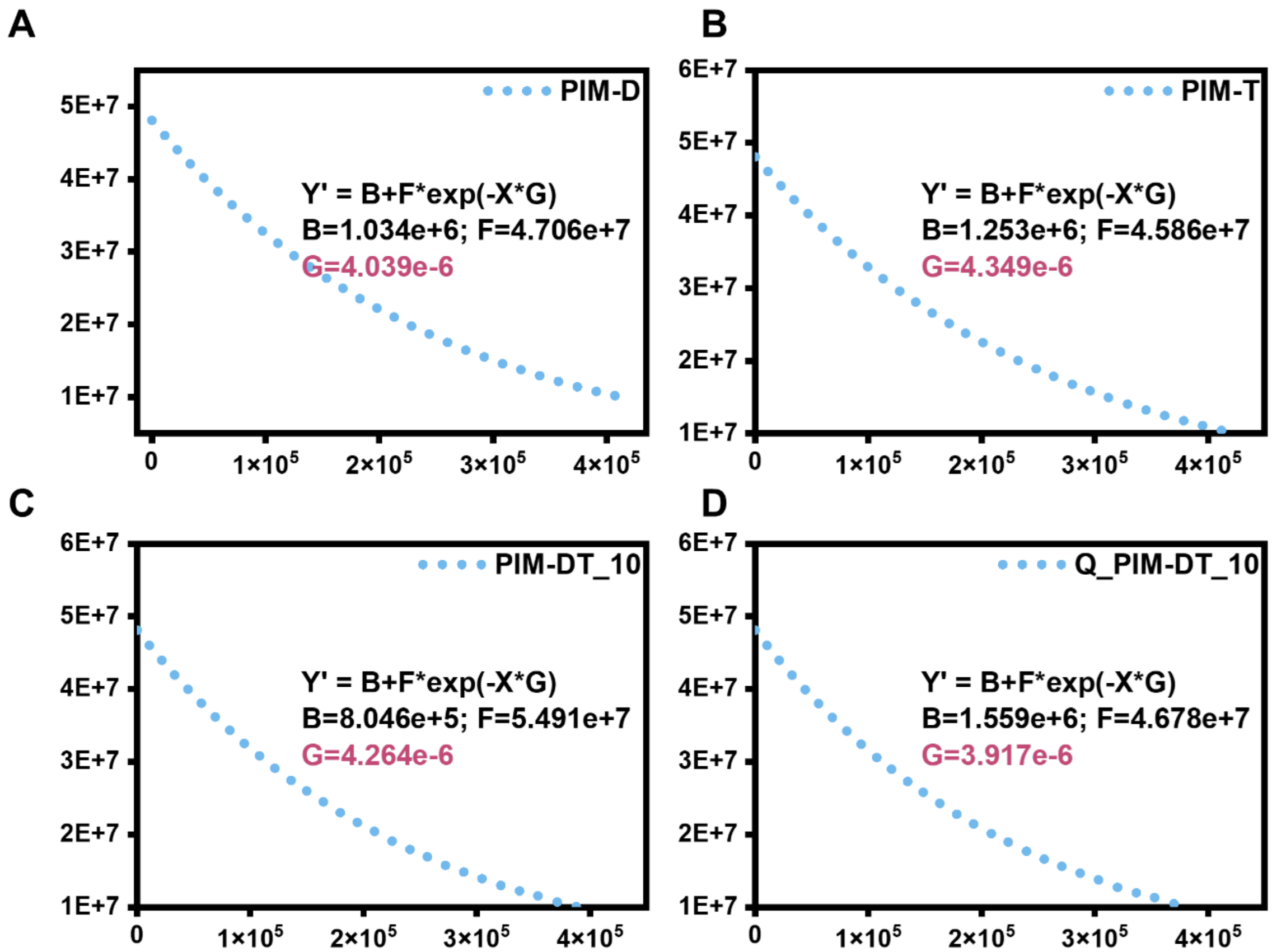

Fig. S26. DOSY ^{1}H NMR data fitting to extract diffusion coefficients of TB-based POP membranes. (**A–D)** Plots of DOSY ^{1}H NMR signal intensity as a function of magnetic gradient strength for **(A)** PIM-D**, (B)** PIM-T**, (C)** PIM-DT_10**,** and **(D)** Q_PIM-DT_10**.** Solid-line fitting follows an exponential decay model, and the fitted G value ($cm^2{\cdot}s^{-1}$) is taken as the apparent diffusion coefficient.

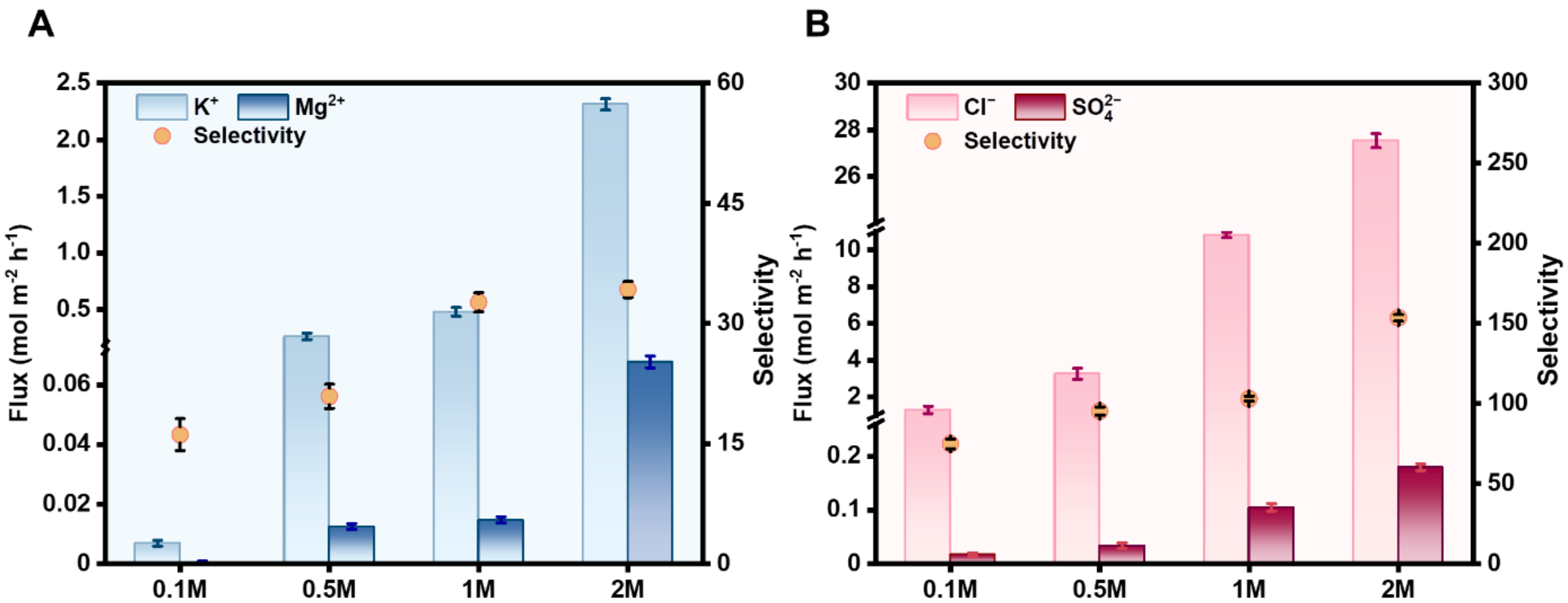

Fig. S27. Concentration-dependent ion flux and selectivity of Q_PIM-DT_10 under mixed-salt gradients at 5 mA·cm^{-2}. (**A**) K^+ and Mg^{2+} fluxes and the corresponding K^+/Mg^{2+} selectivity measured at total mixed-salt concentrations of 0.1, 0.5, 1.0, and 2.0 M. (**B**) Cl^- and SO_4^{2-} fluxes and the corresponding Cl^-/SO_4^{2-} selectivity measured under the same concentration gradients.

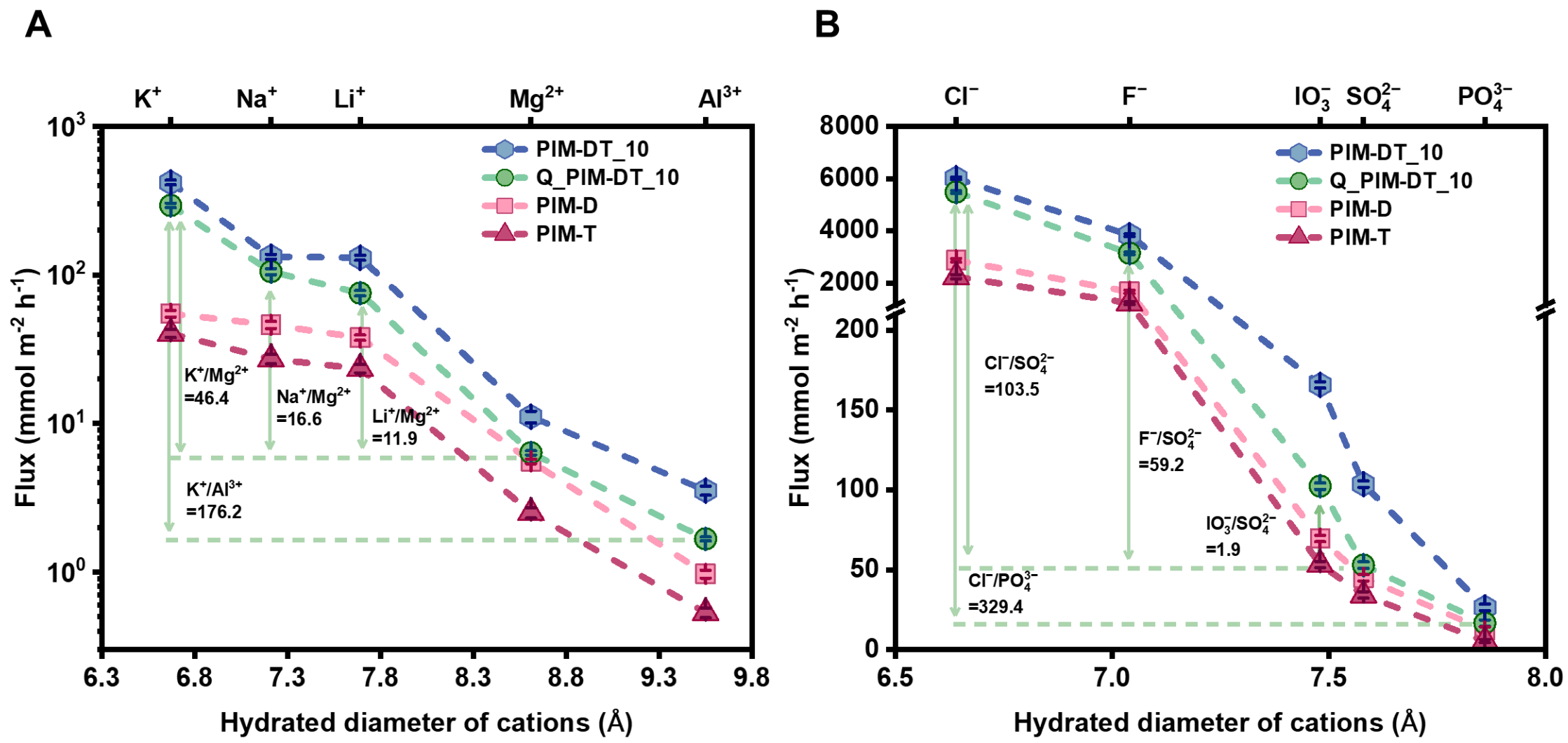

Fig. S28. Hydrated-ion-size-dependent permeation of mixed salts under electrodialysis. (**A**) Cation fluxes (K^+, Na^+, Li^+, Mg^{2+}, and Al^{3+}) through TB-based POP membranes as a function of hydrated cation diameter measured under mixed-salt electrodialysis at 5 mA·cm^{-2} (every single salt: 0.1 M), with the corresponding mono-/multivalent selectivity ratios indicated. (**B**) Anion fluxes with the corresponding mono-/multivalent selectivities (Cl^-, F^-, IO_3^-, SO_4^{2-}, and PO_4^{3-}) as a function of hydrated anion diameter under the same conditions.

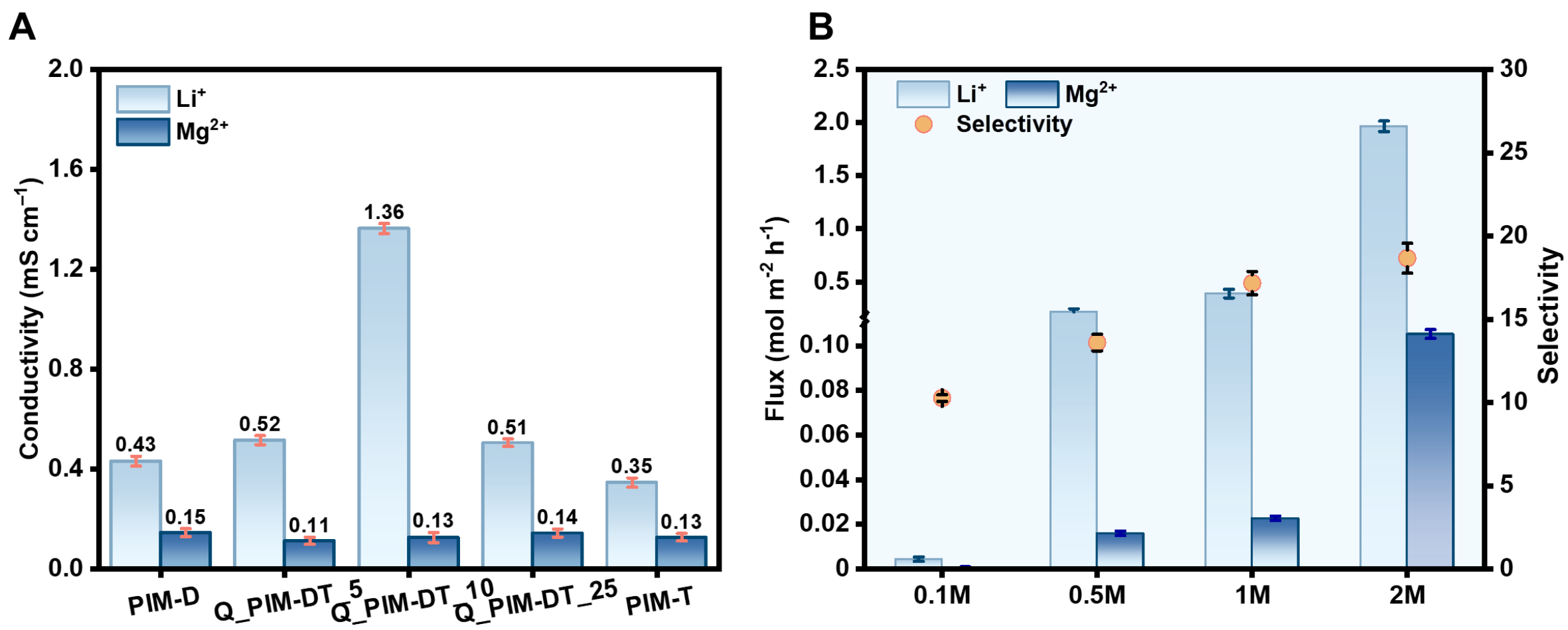

Fig. S29. Electrochemical Li^+/Mg^{2+} separation performance of Q_PIM-DT_10. (**A**) Corresponding Li^+ and Mg^{2+} conductivities of the Q_PIM-DT_10 derived from the I–V measurements. (**B**) Li^+ and Mg^{2+} fluxes and the corresponding Li^+/Mg^{2+} selectivity measured at total mixed-salt concentrations of 0.1, 0.5, 1.0, and 2.0 M at 5 mA·cm^{-2}.

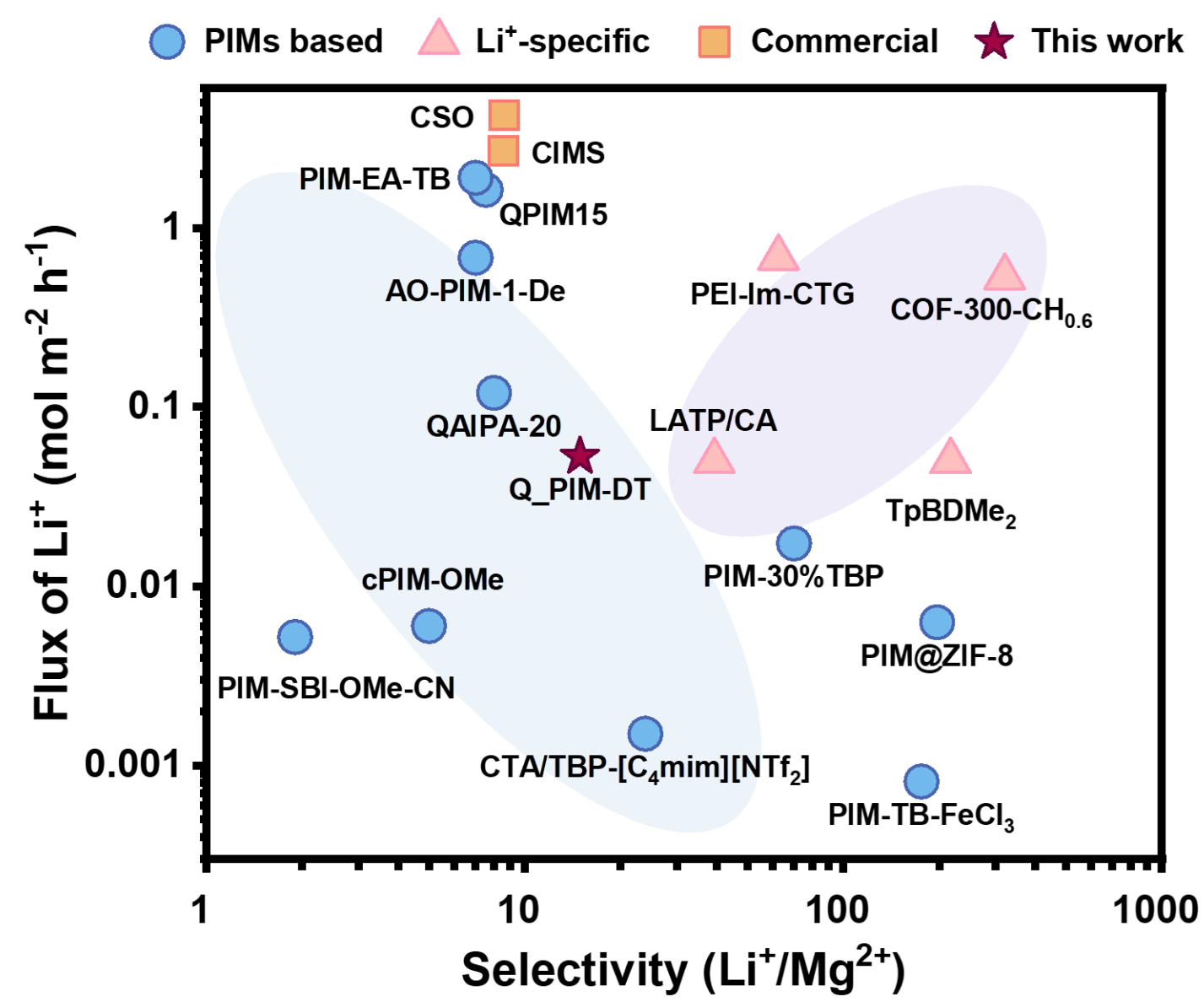

Fig. S30. Performance comparison of Q_PIM-DT with previously reported PIM-based, commercial CEM, and Li^+-specific selective membranes.

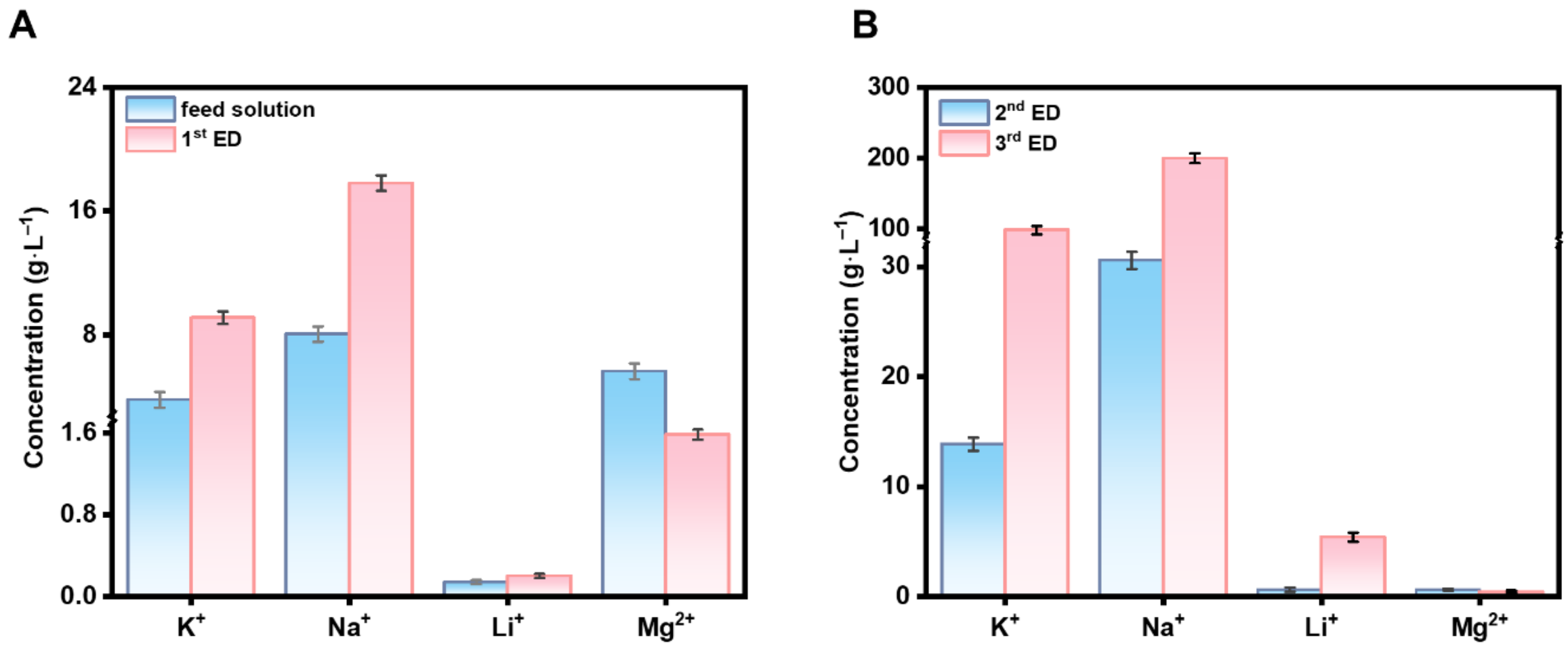

Fig. S31. Evolution of solution composition during the three-stage electrodialysis process. (**A**) Ion concentrations in the initial feed solution and the concentrate after 1 h of the 1st ED stage. (**B**) Ion concentrations in the concentrates collected after 1 h operation of the 2nd and 3rd ED stages.

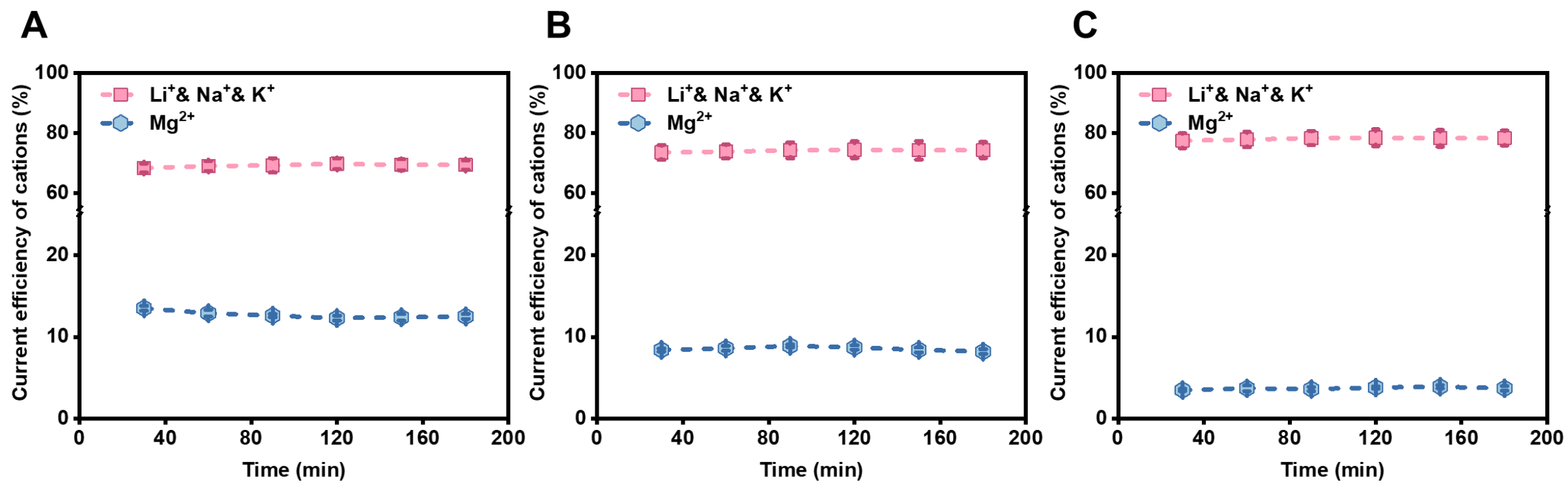

Fig. S32. Current efficiencies of mono- and multivalent cations in the (A) first-stage electrodialysis, (B) second-stage electrodialysis, and (C) third-stage electrodialysis.

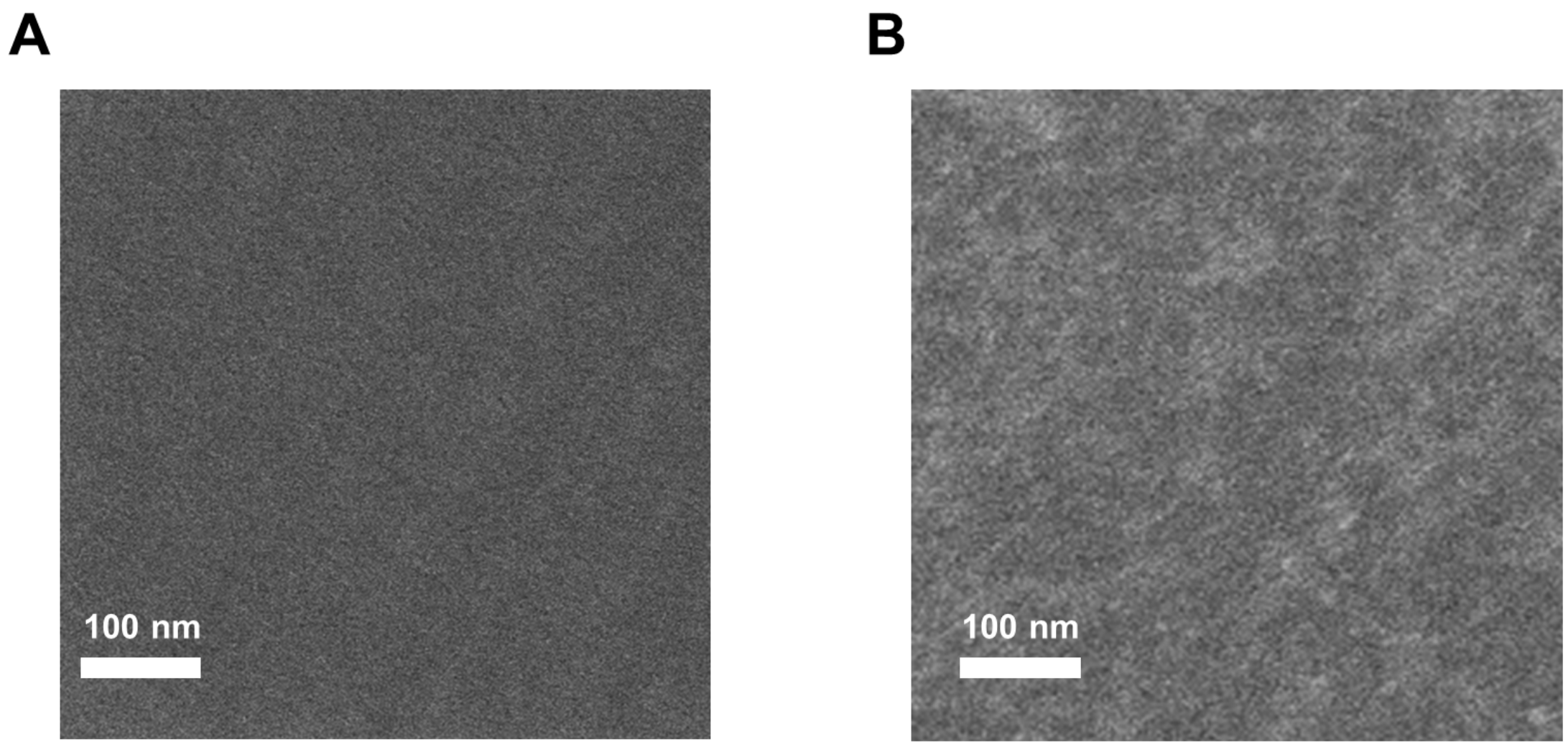

Fig. S33. Surface morphology evolution of the Q_PIM-DT_10 membrane after extended cycling. (**A**) Pristine membrane. (**B**) Membrane after 400 h of operation, showing the retained nanoscale surface texture after long-term use.

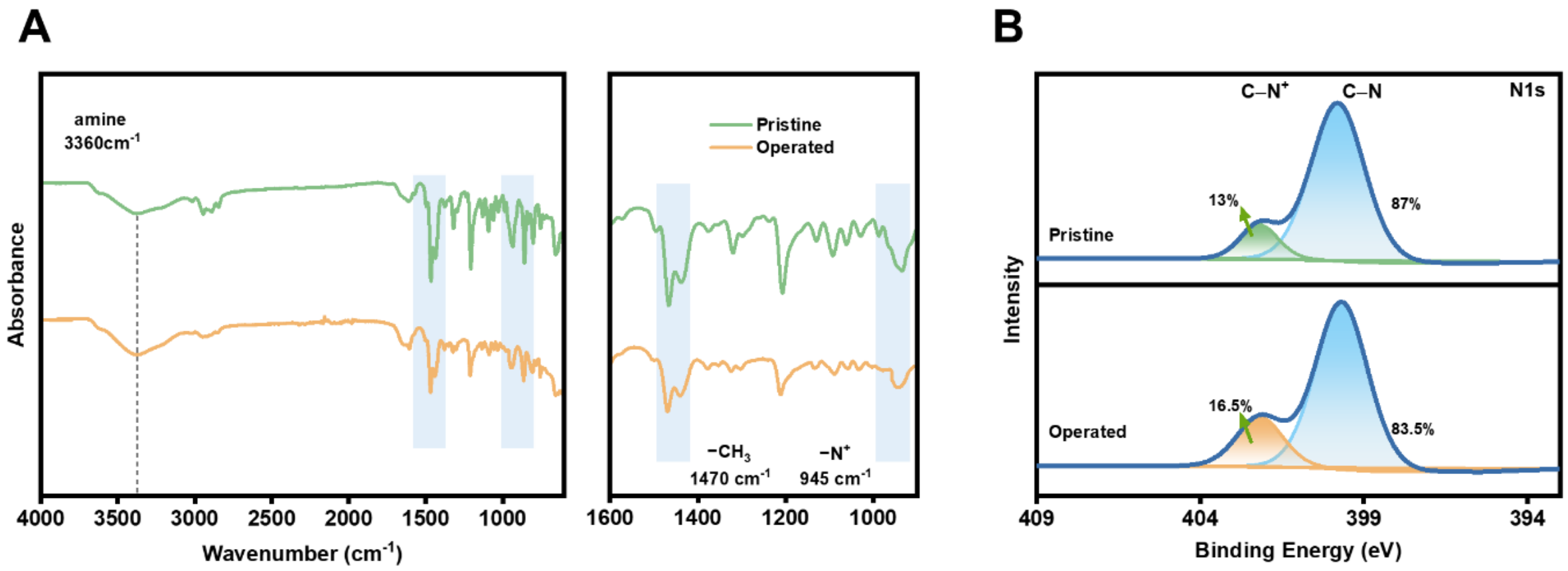

Fig. S34. Chemical-structure stability of Q_PIM-DT_10 after extended cycling. (**A)** FT-IR spectra of pristine and operated membranes after 400 h, with characteristic bands highlighted. (**B)** N 1s core-level XPS spectra before and after operation, showing the retained nitrogen chemical states after long-term cycling.

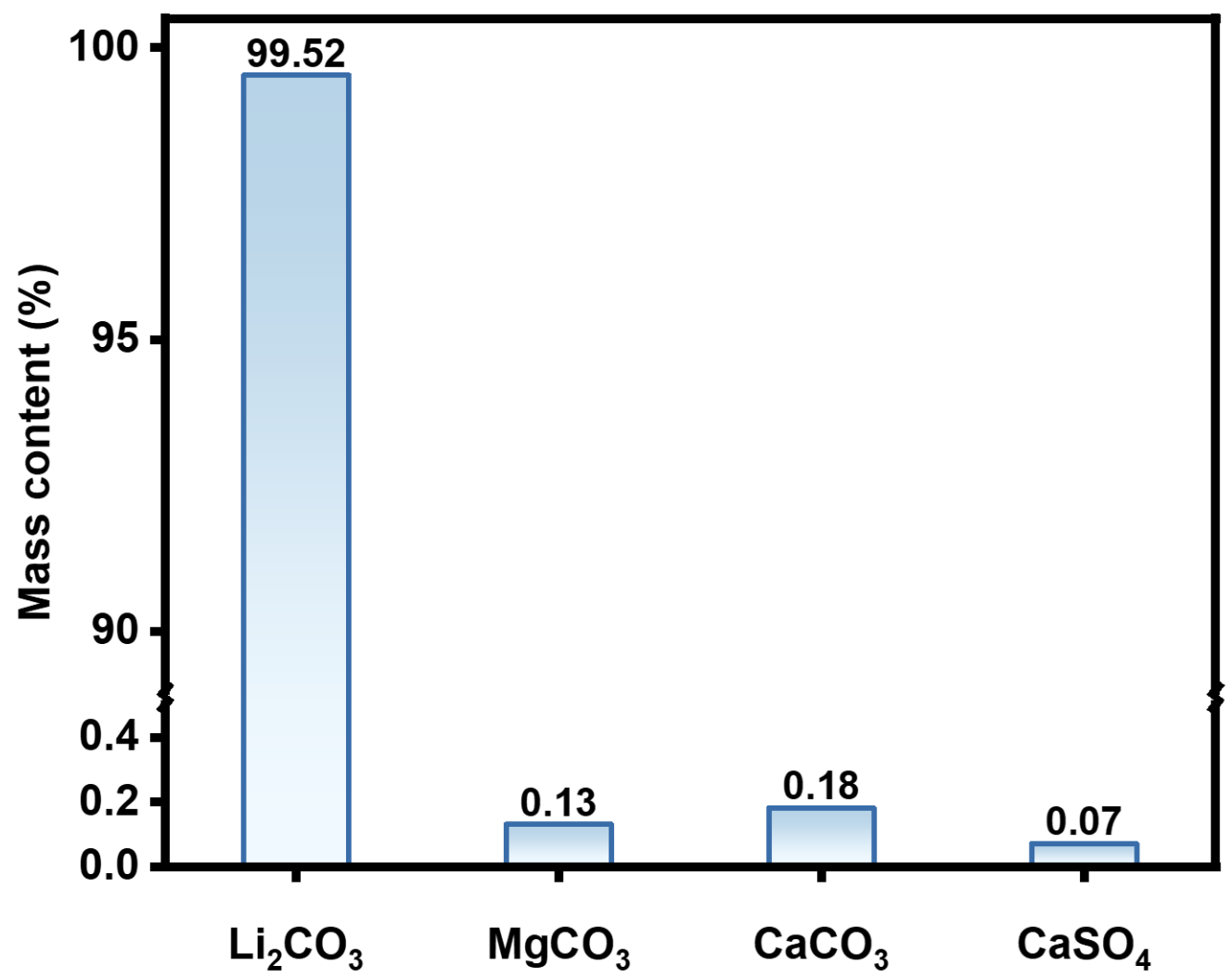

Fig. S35. The mass composition of the final product after three-stage electrodialysis.

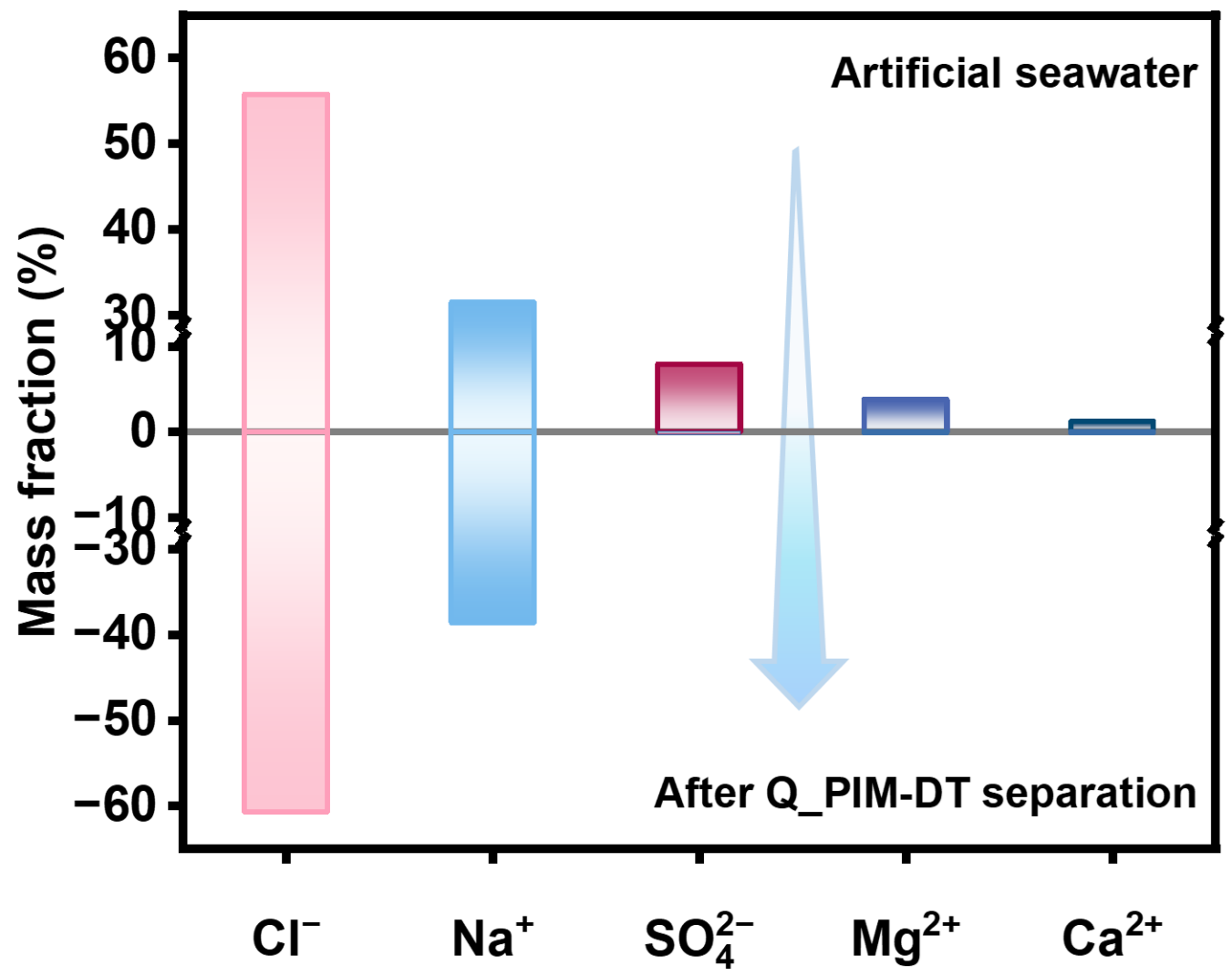

Fig. S36. Ion composition of artificial seawater before and after membrane separation (8-hour test).

Table S1.

Ion concentrations of the simulated solution of East Taijiner Salt Lake (*86*).

**Ions**	**Source salt**	**Mass concentration ($g \cdot L^{-1}$)**
K^+	KCl	3.793
Na^+	NaCl	8.051
Li^+	LiCl	0.141
Ca^{2+}	$CaCl_2$	0.434
Mg^{2+}	$MgCl_2 \cdot 6H_2O$	5.650
Cl^-	KCl, NaCl, LiCl, $CaCl_2$, $MgCl_2 \cdot 6H_2O$	33.82
SO_4^{2-}	Na_2SO_4	22.33

Table S2.

Comparison of the separation performances of Cl^-/SO_4^{2-} and Na^+/Mg^{2+} in this study with those reported in the literature.

Membranes	Material	Selectivity (Cl^-/SO_4^{2-})	Selectivity (Na^+/Mg^{2+})	Ref
PIP@H20+IL1	Polyamide	194.78	11.28	
PEI@H2O+IL2	Polyamide	47.56	279.35	*(54)*
PEI@H2O+IL3	Polyamide	26.58	73.7	
(PIP+SDS) / TMC	Polyamide	182	12.83	*(55)*
PES/CNT/PATFC-2	Polyamide	85.5	12.69	*(56)*
U-iTFC-1%	Polyamide	70.2	30.1	*(57)*
MCPM-2.0	Polyamide	48.2	43.4	*(58)*
G5-TAC2/PIP	Polyamide	65.8	65.8	*(59)*
C-PANF	Polyamide	144	2.6	*(60)*
Asymmetric polyamide nanofilm	Polyamide	74	2.9	*(61)*
N-TFN	Polyamide	46.8	43.5	*(62)*
PAN-Hl	Polyamide	0.76	3.23	*(63)*
G5-PS/PIP	Polyamide	131	7.4	*(64)*
PA-PES/PVP	Polyamide	20.07	2.12	*(32)*
Nanofilms	Polyester	187	13	*(65)*
PIP-NMIP1800	Polyamide	39	15.6	*(66)*
PA0.5-PA1	Polyamide	64.5	50.3	
PA0.25-PA1	Polyamide	43.3	52.7	*(67)*
PA0.75-PA1	Polyamide	50.1	41	
P(MPC-coAEMA)-incorporated PA	Polyamide	47.6	5.3	*(68)*
20.1-i-TFC membrane	Polyamide	34.7	1.1	*(69)*
rGO-10 membrane	GO-based	12.4	0.68	*(70)*
α-M3 membrane	2D membrane	3.39	2.017	*(71)*
TFC-TBDA/PIP	PIMs	78.6	0.7	*(72)*

membrane				
COF-170/PAN	COF	58	35	*(73)*
pDA/TpPa(W/E)-COF	COF	59.4	1.71	*(74)*
pDA/TpPa(W/E)-COF	COF	101.6	1.71	
TpPa–SO3H/TpPa/MPAN	COF	12.9	0.78	*(75)*
p-TpPa-C≡C	COF	70.1	102.4	*(76)*
PA-PIP/Fe-COF/PES	COF	21.9	41.6	*(77)*
ZIF-8@ZNPM	MOF	6.8	1.01	*(78)*
PES-PIP/PPNs-TMC	MOF	9.6	1.67	*(79)*
Al-MOF membrane	MOF	1	4	*(80)*
Q_PIM-D	POPs	36.84	8.278	This work
Q_PIM-T	POPs	35.72	10.836	
Q_PIM-DT_10	POPs	151.79	38.837	
PIM-DT_10	POPs	58.035	11.957	

Table S3.

Comparison of the Li^+/Mg^{2+} separation performance of this study with those reported PIM-based, commercial CEM, and Li^+-specific selective membranes in the literature.

**Membranes**	**Flux of Li^+ ($mol\ m^{-2}\ h^{-1}$)**	**Selectivity (Li^+/Mg^{2+})**	**Ref**
PIM@ZIF-8	0.0063	196.88	*(87)*
PIM-TB-FeCl3	0.000812	176	*(88)*
PIM-EA-TB	1.63	7.53	*(19)*
CTA/TBP-[C_4mim][NTf_2]	0.0015	23.9	*(89)*
PIM-30%TBP	0.0173	70.0	*(90)*
QPIM15	1.9	7.0	*(91)*
QAIPA-20	0.12	8.0	*(92)*
AO-PIM-1-De	0.68	7	*(47)*
PIM-SBI-OMe-CN	0.0052	1.9	*(47)*
cPIM-OMe	0.006	5	*(47)*
CSO	4.26	8.6	*(93)*
CIMS	2.69	8.57	*(93)*
COF-300-$CH_{0.6}$	0.2	321	*(94)*
$TpBDMe_2$	0.05	217	*(95)*
AIP-PA	0.05	78	*(96)*
QAIPA-15	0.013	20	*(97)*
Q_PIM-DT	0.053	14.9	This work

Table S4.
Chemical composition of artificial seawater pre- and post-membrane separation.

Ions	Ion concentration (mol L^{-1})	Ion mass fraction (%)	
		Artificial seawater	After Q_PIM-DT separation
Na^{+}	0.466	31.42	38.63
Mg^{2+}	0.053	3.78	0.13
Ca^{2+}	0.010	1.18	0.12
Cl^{-}	0.536	55.73	60.75
SO_4^{2-}	0.028	7.89	0.37

Description of supplementary data

Dataset S1. Raw data for the CO_2 adsorption isotherms, corresponding pore-size distribution, and XPS survey spectra shown in Figure. 1.

Dataset S2. Raw data corresponding to the SAXS profiles, temperature-dependent Cl^- conductivity, ^1H DOSY NMR spectra, and calculated water diffusion coefficients presented in Figure. 3.

Dataset S3. Raw data for the ion separation performance metrics shown in Figure. 4, including ion flux, selectivity, and permselectivity.

Dataset S4. Raw data corresponding to the potential of mean force (PMF), radial distribution function (RDF), and mean squared displacement (MSD) simulation results presented in Figure. 5.

Dataset S5. Raw data corresponding to the Mg/Li ratio evolution during multistage electrodialysis, long-term operational stability, and the XRD patterns of the obtained products presented in Figure. 6.

Dataset S6. Raw data for mechanical properties shown in Figure. S4.

Dataset S7. Raw data underlying the thermogravimetric (TGA) curves shown in Figure. S5.

Dataset S8. Raw data for the N_2 sorption isotherms shown in Figure. S6.

Dataset S9. Raw data for the FTIR spectra shown in Figure. S7.

Dataset S10. Raw data for the N 1s core-level XPS spectra shown in Figure. S8.

Dataset S11. Raw data for the Ion exchange capacity (IEC) shown in Figure. S9.

Dataset S12. Raw data for the Zeta Potentials shown in Figure. S10.

Dataset S13. Raw data for the cation ion transport characteristics shown in Figure. S15.

Dataset S14. Raw data for the anion ion transport characteristics shown in Figure. S16.

Dataset S15. Raw data for the temperature-dependent ionic conductivity of different ions shown in Figure. S17.

Dataset S16. Raw data for the ion-size-dependent transport and activation energetics shown in Figure. S18.

Dataset S17. Raw data for the Simulated pore size distribution shown in Figure. S19.

Dataset S18. Raw data for the dual-salt electrodialysis experiments shown in Figure. S21.

Dataset S19. Raw data for the dual-salt diffusion dialysis experiments shown in Figure. S22.

Dataset S20. Raw data for the water uptake and swelling ratio shown in Figure. S24.

Dataset S21. Raw data for the water contact angles shown in Figure. S25.

Dataset S22. Raw data for the DOSY ^{1}H NMR analysis and fitting used to extract diffusion coefficients shown in Figure. S26.

Dataset S23. Raw data for the concentration-dependent ion flux and selectivity shown in Figure. S27.

Dataset S24. Raw data for the mixed-salt electrodialysis experiments shown in Figure. S28.

Dataset S25. Raw data for the Electrochemical Li^{+}/Mg^{2+} separation performance shown in Figure. S29.

Dataset S26. Raw data for the evolution of solution composition during the three-stage electrodialysis process shown in Figure. S31.

Dataset S27. Raw data for the Current efficiencies shown in Figure. S32.

Dataset S28. Raw data for the chemical-structure stability after extended cycling shown in Figure. S34.

Dataset S29. Raw data for the mass composition of the final product after three-stage electrodialysis shown in Figure. S35.

Dataset S30. Raw data for the ion composition of artificial seawater before and after membrane separation shown in Figure. S36.

File S1. Raw files for all molecular simulations reported in this study.

REFERENCES

1. P. Zuo, C. Ye, Z. Jiao, J. Luo, J. Fang, U. S. Schubert, N. B. McKeown, T. L. Liu, Z. Yang, T. Xu, Near-frictionless ion transport within triazine framework membranes. *Nature* **617**, 299–305 (2023).

2. W. Xu, Y. Wang, Y. Wu, F. Xu, L. Dai, K. Qu, J. Wang, J. Wu, L. Lei, S. Li, Z. Xu, Sub-2-nm channels within covalent triazine framework enable fast proton-selective transport in flow battery membrane. *Adv. Funct. Mater.* **33**, 2300138 (2023).

3. J.-N. Chang, K. Yang, L.-N. Wang, J. Wang, Y.-Q. Lan, C. Wang, Plasmonic ion diode membrane (PIDM) for enhanced nanofluidic ion transport. *Angew. Chem. Int. Ed.* **64**, e202502591 (2025).

4. Y. Huang, C. Liao, Q. Song, Z. Yuan, X. Li, Chemically cross-linked polybenzimidazole membranes with ion-conductive sub-nanometer channels for zinc-iron flow batteries. *Angew. Chem. Int. Ed.* **64**, e202511744 (2025).

5. K. Liu, R. Epsztein, S. Lin, L. Liu, J. Qu, M. Sun, Electrostatic-driven dehydration of ions in nanoporous membranes. *Sci. Adv.* **11**, eadv0174 (2025).

6. C. Hu, H. W. Kang, S. W. Jung, M. L. Liu, Y. J. Lee, J. H. Park, N. Y. Kang, M. G. Kim, S. J. Yoo, C. H. Park, Y. M. Lee, High free volume polyelectrolytes for anion exchange membrane water electrolyzers with a current density of 13.39 A cm^{-2} and a durability of 1000 h. *Adv. Sci.* **11**, e2306988 (2024).

7. R. Wang, S. Lin, Membrane design principles for ion-selective electrodialysis: An analysis for Li/Mg separation. *Environ. Sci. Technol.* **58**, 3552–3563 (2024).

8. G. Xu, J. Pan, X. Zou, Z. Jin, J. Zhang, P. Fang, Q. Zhang, Z. Sun, F. Yan, High-performance poly(biphenyl piperidinium) type anion exchange membranes with interconnected ion transfer channels: Cooperativity of dual cations and fluorinated side chains. *Adv. Funct. Mater.* **33**, 2302364 (2023).

9. D. Liu, Y. Xie, Z. Zhao, J. Li, J. Pang, Z. Jiang, Structural optimization and performance trade-off strategies for semi-crystalline sulfonated poly (arylene ether ketone) membranes in high-concentration direct methanol fuel cells. *J. Energy Chem.* **85**, 67–75 (2023).

10. M. Atwa, S. Xu, X. Li, S. Dull, Z. Wang, H. Yu, R. Tang, T. Goh, Y. Jung, M. Kiani, M. Lid, H. Nishihara, F. B. Prinz, V. Birss, Controlled distribution of both Pt nanoparticles and ionomer in self-supported nanoporous carbon scaffolds significantly enhances oxygen reduction kinetics and stability. *J. Mater. Chem. A* **13**, 33587–33600 (2025).

11. A. Wang, C. Breakwell, F. Foglia, R. Tan, L. Lovell, X. Wei, T. Wong, N. Meng, H. Li, A. Seel, M. Sarter, K. Smith, A. Alvarez-Fernandez, M. Furedi, S. Guldin, M. M. Britton, N. B. McKeown, K. E. Jelfs, Q. Song, Selective ion transport through hydrated micropores in polymer membranes. *Nature* **635**, 353–358 (2024).

12. Y. Ma, C. Hu, G. Yi, Z. Jiang, X. Su, Q. Liu, J. Y. Lee, S. Y. Lee, Y. M. Lee, Q. Zhang, Durable multiblock poly (biphenyl alkylene) anion exchange membranes with microphase separation for hydrogen energy conversion. *Angew. Chem. Int. Ed.* **62**, e202311509 (2023).

13. Y. J. Shin, M. Kim, H. Kim, Y. Y. Kim, N. H. Kwon, A. Machida, Y. Nakahira, S. Kim, K. Sakaki, W. B. Lee, E. S. Cho, Surface-engineered asymmetric covalent organic framework membranes with polymers for improved ion transport. *ACS Nano* **19**, 34150–34162 (2025).

14. Y. Liu, J. Song, Z. Liu, J. Chen, D. Wang, H. Zhi, J. Tang, Y. Zhang, N. Li, W. Zhou, M. An, H. Liu, G. Xue, Anti-swelling polyelectrolyte hydrogel with submillimeter lateral confinement for osmotic energy conversion. *Nano Micro Lett.* **17**, 81 (2024).

15. R.-J. Mo, S. Chen, L.-Q. Huang, X.-L. Ding, S. Rafique, X.-H. Xia, Z.-Q. Li, Regulating ion affinity and dehydration of metal-organic framework sub-nanochannels for high-precision ion separation. *Nat. Commun.* **15**, 2145 (2024).

16. W. Meng, S. Chen, M. Wu, F. Gao, Y. Hou, X. Zhan, W. Hu, L. Liang, Q. Zhang, Dehydration-enhanced ion recognition of triazine covalent organic frameworks for high-resolution Li^+/Mg^{2+} Separation. *Angew. Chem. Int. Ed.* **64**, e202422423 (2025).

17. T. Xu, B. Wu, L. Hou, Y. Zhu, F. Sheng, Z. Zhao, Y. Dong, J. Liu, B. Ye, X. Li, L. Ge, H. Wang, T. Xu, Highly ion-permselective porous organic cage membranes with hierarchical channels. *J. Am. Chem. Soc.* **144**, 10220–10229 (2022).

18. R. Tan, H. He, A. Wang, T. Wong, Y. Yang, S. Iguodala, C. Ye, D. Liu, Z. Fan, M. Furedi, G. He, S. Guldin, D. J. L. Brett, N. B. McKeown, Q. Song, Interfacial engineering of polymer membranes with intrinsic microporosity for dendrite-free zinc metal batteries. *Angew. Chem. Int. Ed.* **63**, e202409322 (2024).

19. Z. Li, H. Li, C. Jin, J. Li, J. Bao, X. Zhang, N. Zhang, G. He, C. Chen, Y. Song, Confined in situ synthesis of uniformly distributed mixed matrix membranes via solvent evaporation for efficient CO_2 capture. *Adv. Funct. Mater.* **35**, e2420713 (2025).

20. T. H. Lee, M. Balcik, W.-N. Wu, I. Pinnau, Z. P. Smith, Dual-phase microporous polymer nanofilms by interfacial polymerization for ultrafast molecular separation. *Sci. Adv.* **10**, eadp6666 (2024).

21. S. Guo, C. Fang, J. Li, X. Wang, W. Feng, H. Guo, M. Xie, Y. Zhuang, Y. M. Lee, L. Zhu, Microporous polymer membranes: Molecular stents enhanced solvent-accessibility for organic solvent transport. *Adv. Sci.* **12**, e16748 (2025).

22. R. Tan, A. Wang, R. Malpass-Evans, R. Williams, E. W. Zhao, T. Liu, C. Ye, X. Zhou, B. P. Darwich, Z. Fan, L. Turcani, E. Jackson, L. Chen, S. Y. Chong, T. Li, K. E. Jelfs, A. I. Cooper, N. P. Brandon, C. P. Grey, N. B. McKeown, Q. Song, Hydrophilic microporous membranes for selective ion separation and flow-battery energy storage. *Nat. Mater.* **19**, 195–202 (2020).

23. Y. Wang, N. Alaslai, B. Ghanem, X. Ma, X. Hu, M. Balcik, Q. Liu, M. A. Abdulhamid, Y. Han, M. Eddaoudi, I. Pinnau, Hydroxyl-functionalized polymers of intrinsic microporosity and dual-functionalized blends for high-performance membrane-based gas separations. *Adv. Mater.* **36**, e202406076 (2024).

24. M. J. Baran, M. N. Braten, S. Sahu, A. Baskin, S. M. Meckler, L. Li, L. Maserati, M. E. Carrington, Y.-M. Chiang, D. Prendergast, B. A. Helms, Design rules for membranes from

polymers of intrinsic microporosity for crossover-free aqueous electrochemical devices. *Joule* **3**, 2968–2985 (2019).

25. E. Yang, M. Kim, Y. Liang, J. Byun, H. Kim, K. C. Mamani, I. S. Kim, H. Choi, Facile fabrication of robust and tight PIMs-based TFC hollow fiber membranes with a semi-interpenetrating polymer network via liquid phase cross-linking for organic solvent nanofiltration. *J. Membr. Sci.* **713**, 123338 (2025).

26. L. Sun, W. Xu, H. Zhang, J. Chu, M. Wang, K. Song, W. Wu, J. Li, Y. Wang, I. Pinnau, X. Ma, In-situ formation of three-dimensional network intrinsic microporous ladder polymer membranes with ultra-high gas separation performance and anti-trade-off effect. *Angew. Chem. Int. Ed.* **64**, e202420742 (2025).

27. X. Yang, Z. Ullah, J. F. Stoddart, C. T. Yavuz, Porous organic cages. *Chem. Rev.* **123**, 4602–4634 (2023).

28. G.-H. He, L. Li, H. Xu, Y. Ji, P. Wang, A polymer membrane with integrated microphase separation and intrinsic microporosity for aqueous organic redox flow batteries. *Joule* **9**, 101976 (2025).

29. Z. Zhang, L. Wen, L. Jiang, Nanofluidics for osmotic energy conversion. *Nat. Rev. Mater.* **6**, 622–639 (2021).

30. R. Wang, R. He, T. He, M. Elimelech, S. Lin, Performance metrics for nanofiltration-based selective separation for resource extraction and recovery. *Nat. Water* **1**, 291–300 (2023).

31. J. S. Chen, J. Wang, J. H. Zhang, Z. Y. Guo, P. P. Zhang, X. F. Guo, J. Liu, Z. Y. Ji, Electronanofiltration membranes with a bilayer charged structure enable high Li^+/Mg^{2+} selectivity. *ACS Appl. Mater. Interfaces* **16**, 6632–6643 (2024).

32. R. Wang, M. Sheng, X. T. Liu, Q. Y. Wang, Z. Wang, L. Wang, Electrosorption theoretically outperforms nanofiltration and electrodialysis for direct lithium extraction from brines. *Environ. Sci. Technol.* **60**, 1577–1595 (2026).

33. Y. Wu, C. Lu, C. Hu, J. Qu, Combined effect of electrostatic interaction and steric sieving during ion transport in nanochannels. *Environ. Sci. Technol.* **59**, 11377–11388 (2025).

34. M. Wang, P. Zhang, X. Liang, J. Zhao, Y. Liu, Y. Cao, H. Wang, Y. Chen, Z. Zhang, F. Pan, Z. Zhang, Z. Jiang, Ultrafast seawater desalination with covalent organic framework membranes. *Nat. Sustain.* **5**, 518–526 (2022).

35. H. Sun, N. Gan, Y. Lin, B. Wu, Y. Qiu, J. Su, Z. Zhou, F. Zou, J. Yu, H. Matsuyama, Ultramicroporous Tröger's base framework membranes with ionized sub-nanochannels for efficient acid/alkali recovery. *Adv. Sci.* **12**, e2414280 (2025).

36. B. Zhang, H. Zhang, Y. Wang, S. Fang, Adsorption behavior and mechanism of amine/quaternary ammonium lignin on tungsten. *Int. J. Biol. Macromol.* **216**, 882–890 (2022).

37. D. Elizebath, B. Vedhanarayanan, A. Dhiman, R. K. Mishra, C. N. Ramachandran, T.-W. Lin, V. K. Praveen, Spontaneous curvature induction in an artificial bilayer membrane. *Angew. Chem. Int. Ed.* **63**, e202403900 (2024).

38. B. A. Corzo, H. Hernández-Martínez, E. J. Aldeco-Pérez, J. Cárdenas, V. Lara, L. I. Olvera, Functionalized imidazolium ether-free polymer backbones with ion transport channels and catalytic activity. *ACS Mater. Au* **5**, 508–521 (2025).

39. H. Amiri, K. L. Shepard, C. Nuckolls, R. Hernández Sánchez, Single-walled carbon nanotubes: Mimics of biological ion channels. *Nano Lett.* **17**, 1204–1211 (2017).

40. A. Wang, R. Tan, D. Z. Liu, J. Lu, X. Wei, A. Alvarez-Fernandez, C. Ye, C. Breakwell, S. Guldin, A. R. Kucernak, K. E. Jelfs, N. P. Brandon, N. B. McKeown, Q. Song, Ion-selective microporous polymer membranes with hydrogen-bond and salt-bridge networks for aqueous organic redox flow batteries. *Adv. Mater.* **35**, e2210098 (2023).

41. G. Suresh, Y. Scindia, A. Pandey, A. Goswami, Self-diffusion coefficient of water in Nafion-117 membrane with different monovalent counterions: A radiotracer study. *J. Membr. Sci.* **250**, 39–45 (2005).

42. L. M. Thieu, L. Zhu, A. G. Korovich, M. A. Hickner, L. A. Madsen, Multiscale tortuous diffusion in anion and cation exchange membranes. *Macromolecules* **52**, 24–35 (2018).

43. C. Liu, X. Zou, Y. Lv, X. Liu, C. Ma, K. Li, Y. Liu, Y. Chai, L. Liao, J. He, Controllable van der Waals gaps by water adsorption. *Nat. Nanotechnol.* **19**, 448–454 (2024).

44. L. Jiang, L. Chen, C. Cao, K. Chen, C. Ji, Y. Zhang, F. Gan, Coupling CO_2 capture process with electrochemically enhanced membrane distillation system for lithium-ion battery recovery: Reagent-Saving and environmental footprint reducing. *Water Res.* **272**, 122976 (2025).

45. H. Cho, J. Kim, C.-S. Han, Multi-ion transport in reverse electrodialysis: A validated model for design and optimization. *Desalination* **586**, 117746 (2024).

46. J. W. Colson, A. R. Woll, A. Mukherjee, M. P. Levendorf, E. L. Spitler, V. B. Shields, M. G. Spencer, J. Park, W. R. Dichtel, Oriented 2D covalent organic framework thin films on single-layer graphene. *Science* **332**, 228–231 (2011).

47. D. Yang, Y. Yang, T. Wong, S. Iguodala, A. Wang, L. Lovell, F. Foglia, P. Fouquet, C. Breakwell, Z. Fan, Y. Wang, M. M. Britton, D. R. Williams, N. Shah, T. Xu, N. B. McKeown, M.-M. Titirici, K. E. Jelfs, Q. Song, Solution-processable polymer membranes with hydrophilic subnanometre pores for sustainable lithium extraction. *Nat. Water* **3**, 319–333 (2025).

48. Q.-W. Meng, J. Li, Z. Xing, W. Xian, Z. Lai, Z. Dai, S. Wang, L. Zhang, H. Yin, S. Ma, Q. Sun, Regulation of ion binding sites in covalent organic framework membranes for enhanced selectivity under high ionic competition. *ACS Nano* **19**, 12080–12089 (2025).

49. Y. Li, R. Jia, R. Tan, Z. Hong, J. Gu, P. Cui, S. Zhang, H. Shao, J. Ran, C.-F. Fu, Ionization-engineered two-dimensional confined channels in GO membranes for highly efficient OH^- separation. *AIChE J.* **70**, e18298 (2023).

50. J. Yan, H. Wang, R. Fu, R. Fu, R. Li, B. Chen, C. Jiang, L. Ge, Z. Liu, Y. Wang, T. Xu, Ion exchange membranes for acid recovery: Diffusion dialysis (DD) or selective electrodialysis (SED). *Desalination* **531**, 115690 (2022).

51. W. Ji, N. U. Afsar, B. Wu, F. Sheng, M. A. Shehzad, L. Ge, T. Xu, In-situ crosslinked SPPO/PVA composite membranes for alkali recovery via diffusion dialysis. *J. Membr. Sci.* **590**, 117267 (2019).

52. R. Mondal, S. Pal, U. Chatterjee, Alkylated imidazole moieties in a cross-linked anion exchange membrane facilitate acid recovery with high purity. *ACS Appl. Polym. Mater.* **3**, 1544–1554 (2021).

53. M. Shi, L. A. Diaz, J. R. Klaehn, A. D. Wilson, T. E. Lister, Li_2CO_3 Recovery through a carbon-negative electrodialysis of lithium-ion battery leachates. *ACS Sustain. Chem. Eng.* **10**, 11773–11781 (2022).

54. F. Soyekwo, C. Liu, H. Wen, S. Raza, Polyamide–based membranes with nanoscale homogeneity and asymmetric structure for ultrafast ion separation. *J. Membr. Sci.* **685**, 121921 (2023).

55. Y. Liang, Y. Zhu, C. Liu, K.-R. Lee, W.-S. Hung, Z. Wang, Y. Li, M. Elimelech, J. Jin, S. Lin, Polyamide nanofiltration membrane with highly uniform sub-nanometre pores for sub-1 Å precision separation. *Nat. Commun.* **11**, 2015 (2020).

56. G. Gong, P. Wang, Z. Zhou, Y. Hu, New insights into the role of an interlayer for the fabrication of highly selective and permeable thin-film composite nanofiltration membrane. *ACS Appl. Mater. Interfaces* **11**, 7349–7356 (2019).

57. C. Ji, C.-W. Lin, S. Zhang, Y. Guo, Z. Yang, W. Hu, S. Xue, Q. J. Niu, R. B. Kaner, Ultrapermeable nanofiltration membranes with tunable selectivity fabricated with polyaniline nanofibers. *J. Mater. Chem. A* **10**, 4392–4401 (2022).

58. J. Zhao, X. You, G. Wang, J. Yuan, Y. Li, C. Yang, S. Zhang, X. Wang, R. Zhang, H. Wu, Z. Jiang, Mix-charged polyamide membranes via molecular hybridization for selective ionic nanofiltration. *J. Membr. Sci.* **644**, 120051 (2022).

59. Z. L. Qiu, W. H. Yu, Y. J. Shen, B. K. Zhu, L. F. Fang, Janus charged polyamide nanofilm with ultra-high separation selectivity for mono−/divalent ions. *Chem. Eng. J.* **416**, 129023 (2021).

60. Y. Zhang, H. Wang, J. Guo, X. Cheng, G. Han, C. H. Lau, H. Lin, S. M. Liu, J. Ma, L. Shao, Ice-confined synthesis of highly ionized 3D-quasilayered polyamide nanofiltration membranes. *Science* **382**, 202–206 (2023).

61. B. Yuan, S. Zhao, P. Hu, J. Cui, Q. J. Niu, Asymmetric polyamide nanofilms with highly ordered nanovoids for water purification. *Nat. Commun.* **11**, 6102 (2020).

62. S. Han, J. Zhu, A. A. Uliana, D. Li, Y. Zhang, L. Zhang, Y. Wang, T. He, M. Elimelech, Microporous organic nanotube assisted design of high performance nanofiltration membranes. *Nat. Commun.* **13**, 7954 (2022).

63. Y. Zhang, R. Dong, U. R. Gabinet, R. Poling-Skutvik, N. K. Kim, C. Lee, O. Q. Imran, X. Feng, C. O. Osuji, Rapid fabrication by lyotropic self-assembly of thin nanofiltration membranes with uniform 1 nanometer pores. *ACS Nano* **15**, 8192–8203 (2021).

64. Z.-L. Qiu, L.-F. Fang, Y.-J. Shen, W.-H. Yu, B.-K. Zhu, C. Hélix-Nielsen, W. Zhang, Ionic dendrimer based polyamide membranes for ion separation. *ACS Nano* **15**, 7522–7535 (2021).

65. G. Lu, S. Lu, J. Sun, M. W. Boey, W. Shang, J. Wu, A. K. An, Nano-confined supramolecular assembly of ultrathin crystalline polymer membranes for high-performance nanofiltration. *Adv. Funct. Mater.* **34**, 2309913 (2024).

66. Y. Chen, W. Wang, X. Liu, X. Duan, Y. Ren, Nanocapsules induce in situ grafting of polyelectrolytes and structural rearrangement of polyamide layers. *Chem. Eng. Sci.* **308**, 121437 (2025).

67. Q. Zhou, M. Chen, J. Lu, B. Sheng, J. Chen, Q. Zhang, X. Han, Wide-temperature solid polymer electrolytes: Li^+ coordination structure, ionic transport and interphases. *Mater. Horiz.* **12**, 3201–3233 (2025).

68. Y. Lin, X. Yao, Q. Shen, T. Ueda, Y. Kawabata, J. Segawa, K. Guan, T. Istirokhatun, Q. Song, T. Yoshioka, H. Matsuyama, Zwitterionic copolymer-regulated interfacial polymerization for highly permselective nanofiltration membrane. *Nano Lett.* **21**, 6525–6532 (2021).

69. W. L. Li, W. T. Lin, C. Y. Zhu, P. Fu, D. Zhou, X. J. Huang, Z. K. Xu, L. S. Wan, High-performance thin film composite nanofiltration membranes with MoS_2 nanosheet interlayer. *J. Membr. Sci.* **685**, 121956 (2023).

70. Z. Liu, Z. Ma, B. Qian, A. Y. H. Chan, X. Wang, Y. Liu, J. H. Xin, A facile and scalable method of fabrication of large-area ultrathin graphene oxide nanofiltration membrane. *ACS Nano* **15**, 15294–15305 (2021).

71. H. Qian, P. Peng, H. Fan, Z. Yang, L. Yang, Y. Zhou, D. Tan, F. Yang, M. Willatzen, G. Amaratunga, Z. Wang, D. Wei, Horizontal transport in $Ti_3C_2T_x$ MXene for highly efficient osmotic energy conversion from saline-alkali environments. *Angew. Chem. Int. Ed.* **63**, e202414984 (2024).

72. T.-H. Chen, T. Su, L. Xia, L.-Y. Gong, B.-N. Dong, L.-F. Liu, Corolliform morphology thin film composite polyamide membrane constructed via Tröger's base PIM for enhancing nanofiltration separation performances. *Desalination* **548**, 116234 (2023).

73. X.-F. Hu, T. Jiang, H. Fan, Y.-F. Guan, J.-J. Chen, H.-Q. Yu, M. Elimelech, Dual-regulated covalent organic framework membranes with near-theoretical pore sizes for angstrom-scale ion separations. *Sci. Adv.* **11**, eady3587 (2025).

74. F. Yang, J. Guo, C. Han, J. Huang, Z. Zhou, S.-P. Sun, Y. Zhang, L. Shao, Turing covalent organic framework membranes via heterogeneous nucleation synthesis for organic solvent nanofiltration. *Sci. Adv.* **10**, eadr9260 (2024).

75. J. Shen, J. Yuan, B. Shi, X. You, R. Ding, T. Zhang, Y. Zhang, Y. Deng, J. Guan, M. Long, Y. Zheng, R. Zhang, H. Wu, Z. Jiang, Homointerface covalent organic framework membranes for efficient desalination. *J. Mater. Chem. A* **9**, 23178–23187 (2021).

76. M. Wang, Z.-H. Wu, G.-Z. Zhou, X.-Q. Wu, S. Li, T.-Y. Ma, Y. Ren, G. Hai, B. Wang, D.-S. Li, Partitioning COF membrane channels into ultramicroporous and π-electron-rich compartments for multicomponent ion separations. *Angew. Chem. Int. Ed.* **64**, e202514179 (2025).

77. Y.-X. Fang, Y.-F. Lin, Z.-L. Xu, D. Pandaya, S.-J. Xu, Interfacial polymerization modulated by COFs interlayer to fabricate efficient mono/divalent ions separation nanofiltration membrane. *Desalination* **588**, 117977 (2024).

78. Y.-L. Ji, B.-X. Gu, S.-J. Xie, M.-J. Yin, W.-J. Qian, Q. Zhao, W.-S. Hung, K.-R. Lee, Y. Zhou, Q.-F. An, C.-J. Gao, Superfast water transport zwitterionic polymeric nanofluidic membrane reinforced by metal-organic frameworks. *Adv. Mater.* **33**, e2102292 (2021).

79. Y. Chen, T. Zhang, D. Chai, H. Ye, S. Tang, P. Wang, H. Sun, B. Ge, Q. J. Niu, Enhancing the $NaCl/Na_2SO_4$ separation selectivity and chlorine resistance of nanofiltration membranes by incorporating novel designed starch nanoparticles. *Appl. Surf. Sci.* **604**, 154417 (2022).

80. M. Jian, R. Qiu, Y. Xia, J. Lu, Y. Chen, Q. Gu, R. Liu, C. Hu, J. Qu, H. Wang, X. Zhang, Ultrathin water-stable metal-organic framework membranes for ion separation. *Sci. Adv.* **6**, eaay3998 (2020).

81. P. Ramirez, J. Cervera, S. Nasir, M. Ali, W. Ensinger, S. Mafe, Electrochemical impedance spectroscopy of membranes with nanofluidic conical pores. *J. Colloid Interface Sci.* **655**, 876–885 (2024).

82. S. Wang, Y. Huang, Y. Qiang, M. Wu, S. Liang, J. Wang, C. Fang, L. Zhu, Nanoconfined electrostatic interaction for efficient anion sieving in graphene oxide membranes. *Water Res.* **270**, 122855 (2025).

83. D. Wu, Z. Xing, Q. Guo, Z. Lai, J. Yi, Q.-W. Meng, S. Wang, Z. Dai, S. Ma, Q. Sun, Engineering bipolar covalent organic framework membranes for selective acid extraction. *Angew. Chem. Int. Ed.* **64**, e202503945 (2025).

84. B. Wu, Y. Lin, N. Gan, Y. Qiu, H. Sun, J. Su, P. Huang, X. Lin, J. Yu, T. Yoshioka, H. Matsuyama, Bioinspired membrane with Angstrom-scale channels: A versatile nanofluidic platform for efficient single-ion sieving. *AIChE J.* **72**, e70098 (2026).

85. H. Zhang, W. Xu, W. Song, K. Peng, L. Sun, C. Yang, X. Zhang, H. Zhang, B. Ye, X. Liang, Z. Yang, L. Wu, X. Ge, T. Xu, High-performance spiro-branched polymeric membranes for sustainability applications. *Nat. Sustain.* **7**, 910–919 (2024).

86. S.-Y. Sun, L.-J. Cai, X.-Y. Nie, X. Song, J.-G. Yu, Separation of magnesium and lithium from brine using a Desal nanofiltration membrane. *J. Water Process Eng.* **7**, 210–217 (2015).

87. H. Kazemzadeh, J. Karimi-Sabet, J. T. Darian, A. Adhami, Evaluation of polymer inclusion membrane efficiency in selective separation of lithium ion from aqueous solution. *Sep. Purif. Technol.* **251**, 117298 (2020).

88. C. Zhang, Y. Mu, S. Zhao, W. Zhang, Y. Wang, Lithium extraction from synthetic brine with high Mg^{2+}/Li^{+} ratio using the polymer inclusion membrane. *Desalination* **496**, 114710 (2020).

89. L. Xu, X. Zeng, Q. He, T. Deng, C. Zhang, W. Zhang, Stable ionic liquid-based polymer inclusion membranes for lithium and magnesium separation. *Sep. Purif. Technol.* **288**, 120626 (2022).

90. W. Shen, D. Wang, Y. Tian, F. Zhou, Y. Lin, Z. Zhang, J. Xu, C. Yang, Highly permeable and selective polymer inclusion membrane for Li^{+} recovery and underlying enhanced mechanism. *J. Membr. Sci.* **699**, 122671 (2024).

91. J. Ying, Z. Cui, L. Tu, T. Liu, S. Lu, Q. Shen, M. Guo, Y. Wang, Z. Yang, M. Zeng, Z. Yu, X. Cao, Y. Lin, Polymers with intrinsic microporosity engineered via acid-base pairs for highly selective lithium-ion transport channels. *J. Membr. Sci.* **741**, 125059 (2026).

92. C. Bandl, W. Kern, M. Ranz, B. Grabner, Functionalization of PTFE substrates with quaternary ammonium groups—An approach towards anion conducting properties. *Appl. Surf. Sci.* **661**, 160084 (2024).

93. W. Wang, G. Hong, Y. Zhang, X. Yang, N. Hu, J. Zhang, P. Sorokin, L. Shao, Designing an energy-efficient multi-stage selective electrodialysis process based on high-performance materials for lithium extraction. *J. Membr. Sci.* **675**, 121534 (2023).

94. W. Meng, S. Chen, Z. Guo, F. Gao, J. Wang, J. Lu, Y. Hou, Q. He, X. Zhan, M. Qiu, Q. Zhang, Three-dimensional cationic covalent organic framework membranes for rapid and selective lithium extraction from saline water. *Nat. Water* **3**, 191–200 (2025).

95. F. Sheng, B. Wu, X. Li, T. Xu, M. A. Shehzad, X. Wang, L. Ge, H. Wang, T. Xu, Efficient ion sieving in covalent organic framework membranes with sub-2-nanometer channels. *Adv. Mater.* **33**, e2104404 (2021).

96. X.-Y. Xie, H.-Q. Huo, W.-H. Zhang, Y. Zhao, Y.-L. Ji, B. Van der Bruggen, C.-J. Gao, Facile surface modification of cation exchange membranes via Debus-Radziszewski in-situ cross-linking for lithium extraction from salt-lakes. *Chem. Eng. J.* **487**, 150617 (2024).

97. D. Seo, J. Lee, S. R. Kong, G. Sim, Y. Park, Selective lithium extraction from salt-lake brine using LATP-incorporated cellulose membranes in electrically driven systems. *J. Membr. Sci.* **718**, 123676 (2025).
